# Combining genome-wide and transcriptome-wide analyses reveal the evolutionary conservation and functional diversity of aquaporins in cotton

**DOI:** 10.1186/s12864-019-5928-2

**Published:** 2019-07-01

**Authors:** Weixi Li, Dayong Zhang, Guozhong Zhu, Xinyue Mi, Wangzhen Guo

**Affiliations:** State Key Laboratory of Crop Genetics & Germplasm Enhancement, Engineering Research Center of Hybrid Cotton Development Ministry of Education, Nanjing Agricultural University, Nanjing, 210095 Jiangsu Province People’s Republic of China

**Keywords:** *Gossypium*, Aquaporin, Comparative genomics, Evolutionary conservation, Functional diversity

## Abstract

**Background:**

Aquaporins (AQPs) are integral membrane proteins from a larger family of major intrinsic proteins (MIPs) and function in a huge variety of processes such as water transport, plant growth and stress response. The availability of the whole-genome data of different cotton species allows us to study systematic evolution and function of cotton AQPs on a genome-wide level.

**Results:**

Here, a total of 53, 58, 113 and 111 AQP genes were identified in *G. arboreum*, *G. raimondii*, *G. hirsutum* and *G. barbadense*, respectively. A comprehensive analysis of cotton AQPs, involved in exon/intron structure, functional domains, phylogenetic relationships and gene duplications, divided these AQPs into five subfamilies (PIP, NIP, SIP, TIP and XIP). Comparative genome analysis among 30 species from algae to angiosperm as well as common tandem duplication events in 24 well-studied plants further revealed the evolutionary conservation of AQP family in the organism kingdom. Combining transcriptome analysis and Quantitative Real-time PCR (qRT-PCR) verification, most AQPs exhibited tissue-specific expression patterns both in *G. raimondii* and *G. hirsutum*. Meanwhile, a bias of time to peak expression of several AQPs was also detected after treating *G. davidsonii* and *G. hirsutum* with 200 mM NaCl. It is interesting that both *PIP1;4 h/i/j* and *PIP2;2a/e* showed the highly conserved tandem structure, but differentially contributed to tissue development and stress response in different cotton species.

**Conclusions:**

These results demonstrated that cotton AQPs were structural conservation while experienced the functional differentiation during the process of evolution and domestication. This study will further broaden our insights into the evolution and functional elucidation of AQP gene family in cotton.

**Electronic supplementary material:**

The online version of this article (10.1186/s12864-019-5928-2) contains supplementary material, which is available to authorized users.

## Background

Major intrinsic proteins (MIPs) belong to a large superfamily of transmembrane protein channels that present in almost all species including plants [1]. MIPs facilitate special small neutral solutes transport across all kinds of membranes, such as urea [2], CO_2_ [3], H_2_O_2_ [4], ammonia [5], metalloids and ions [6]. The MIP superfamily includes three subfamilies: (1) classical water-selective AQP (CAQP) [7], (2) aquaglyceroporin (AQGP) [8] and (3) super-aquaporin (SAQP) [9, 10]. In general, AQP consists of six transmembrane α helices and five loops (loop A to E) that loops B and E contain highly conserved NPA motifs [11]. Plants have lost AQGP after CAQPs of algal ancestors diversified into PIP (Plasma membrane Intrinsic Protein), TIP (Tonoplast Intrinsic Protein), SIP (Small Intrinsic Protein), XIP (X Intrinsic Protein), HIP (Hybrid Intrinsic Protein), LIP (Large Intrinsic Protein) [12–18] and NIP (NOD-26 like Intrinsic Protein) [19, 20].

Gene duplication is understood to be an important source of evolution and diversity of species. Besides, many researches also provide direct evidence of the importance of duplicate genes in plant adaptation to variable abiotic and biotic environmental factors [21, 22]. Gene duplication includes whole-genome duplication (WGD) and single gene duplication that contains five types, tandem (TD), proximal (PD), retrotransposed (RD), DNA-transposed (DD) and dispersed duplication (DSD) [23, 24]. WGD, or polyploidization, doubles the chromosomes initially and results in a sudden increase in genome size. Paleopolyploidization is prevalent in genome evolution of land plants lineage but not for animals and fungi [25–28]. In addition to WGD, single gene duplication has long been regarded as a universal phenomenon in plant genomes [23, 29]. WGD, single gene duplication and horizontal gene transfers (HGT) may enrich the AQP genetic diversity in natural selections and environmental adaptations [30, 31].

The availability of the genome sequences in different species makes it possible for mining AQPs via integrating bioinformatics methods and next generation sequencing (NGS) data. Totally, 35 members of the AQP family have been identified in *Arabidopsis thaliana* [15], 31 in *Zea mays* [14], 33 in *Oryza sativa* [32], 28 in *Vitis vinifera* [33], 38 in *Sorghum bicolor* [34], 28 in *Brachypodium distachyon* [34], 40 in *Hordeum vulgare* [35], 47 in *Solanum lycopersicum* [36], 66 in *Glycine max* [37], 19 in *Selaginella moellendorffii* [38], 55 in *Populus trichocarpa* [39], 23 in *Physcomitrella patens* [12], 51 in *Linum usitatissimum* [40] and 59 in *Brassica rapa* [41] and so on. Furthermore, the structural and phylogenetic characterization of aquaporin family were also reported [42–44].

Cotton (*Gossypium* spp.) is the most important textile fiber crop and the second-most important oil crop. The most widely cultivated cotton, *Gossypium hirsutum* L. (AADD, AD_1_) and *G. barbadense* L. (AADD, AD_2_), are two tetraploid species, which were originated from chromosome doubling and interspecific hybridization between two closest relatives, an A-genome species, *G. arboreum* (A_2_) and a D-genome species, *G. raimondii* (D_5_) about 1–2 million years ago (MYA) [45, 46]. *G. davidsonii* (D_3_), a D-genome diploid cotton species, occurs in the Cape Region of Baja California Sur, Mexico [47]. Previous studies showed *G. davidsonii* had superior stress tolerance and low levels of genetic variability (amplified fragment length polymorphisms [48] and allozymes [49]), which may be caused by high levels of inbreeding. Recently, the competing of genome sequencing of the four cotton species with different sources, including *G. hirsutum* acc. TM-1 [50–52], *G. barbadense* acc. 3–79 and cv. Hai7124 [51, 52], *G. arboreum* [53] and *G. raimondii* [54, 55], has laid the foundation of research on the AQP family in cotton.

Before the whole genome sequence data were released, Park et al. (2010) reported the 71 AQPs in *G. hirsutum*, including 28 PIPs, 23 TIPs, 12 NIPs, 7 SIPs and 1 XIP, respectively [56]. In this present study, via the released whole genome sequences of different cotton species, we systematically surveyed the structural and functional characterization of cotton AQPs and defined the corresponding relationships. Then, we constructed a phylogenetic tree of the AQP gene family in *G. raimondii*, *A. thaliana* and *O. sativa*, and analyzed intra- and inter-genomic duplication events of these three species. We also analyzed the distribution and homology of AQP family in 30 species from algae to angiosperm and the tandem duplication events of AQP genes in 24 well-studied species. Finally, the expression patterns of AQP genes in different tissues and in response to salt stress were analyzed in *G. raimondii* or *G. davidsonii* and *G. hirsutum*, respectively. The results provide a foundation for further comprehension on the distribution, structure, evolution and functional differentiation of the AQP gene family in cotton and other angiosperms.

## Results

### Genome-wide identification of the AQP gene family in cotton

To identify aquaporins in cotton, a genome-wide mining was carried out using both BLASTp searches with 35 AQP genes from *Arabidopsis* as queries and HMMER [57] searches with MIP domain (PF00230) as the model in the protein database of four cotton species, *G. raimondii*, *G. arboreum*, *G. hirsutum* acc. TM-1 with three sources, *G. barbadense* acc. 3–79 and cv. Hai7124, respectively. After summarizing and comparing the results from different species/accessions of genome databases, a total of 335 AQP genes were identified, including 53, 58, 113 and 111 in *G. arboreum*, *G. raimondii*, *G. hirsutum* and *G. barbadense*, respectively (Additional file 1: Table S1). The nomenclature of *GrAQPs* in *G. raimondii* was defined according to the closest orthologs in *A. thaliana*. Among 35 AQP genes in *A. thaliana*, 10 AQPs (*AtNIP2;1*, *AtNIP3;1*, *AtTIP1;2*, *AtTIP2;2*, *AtTIP3;1*, *AtPIP1;1*, *AtPIP1;3*, *AtPIP1;5*, *AtPIP2;3* and *AtPIP2;6*) had not corresponding orthologs in *G. raimondii*; and 55 *GrAQPs* were designated according to the rest of 25 *AtAQP* orthologs. Other three *GrAQPs*, *GrXIP1;1*, *GrXIP2;1* and *GrXIP2;2*, had not found orthologs in *A. thaliana*. Most *GrAQPs* had only one gene in *G. raimondii*, such as *GrNIP1;1*, *GrNIP4;1* and other 14 *GrAQPs* (Additional file 1: Table S1). Besides, *GrNIP7;1*, *GrPIP2;5* and *GrTIP2;3* had two paralogous genes; *GrNIP1;2* and *GrTIP1;1* had three paralogs; *GrPIP2;2*, *GrPIP2;4*, *GrPIP2;7*, *GrSIP1;1* and *GrTIP1;3* had four; and *GrPIP1;4* had 10 in *G. raimondii,* respectively. Different paralogs were tagged a-j according to their order of the homologous chromosomes. In addition, the corresponding orthologs in *G. arboreum*, *G. hirsutum* and *G. barbadense* were named as *GaAQP*, *GhAQP*, and *GbAQP* with the same number, respectively.

**Fig. 1:**
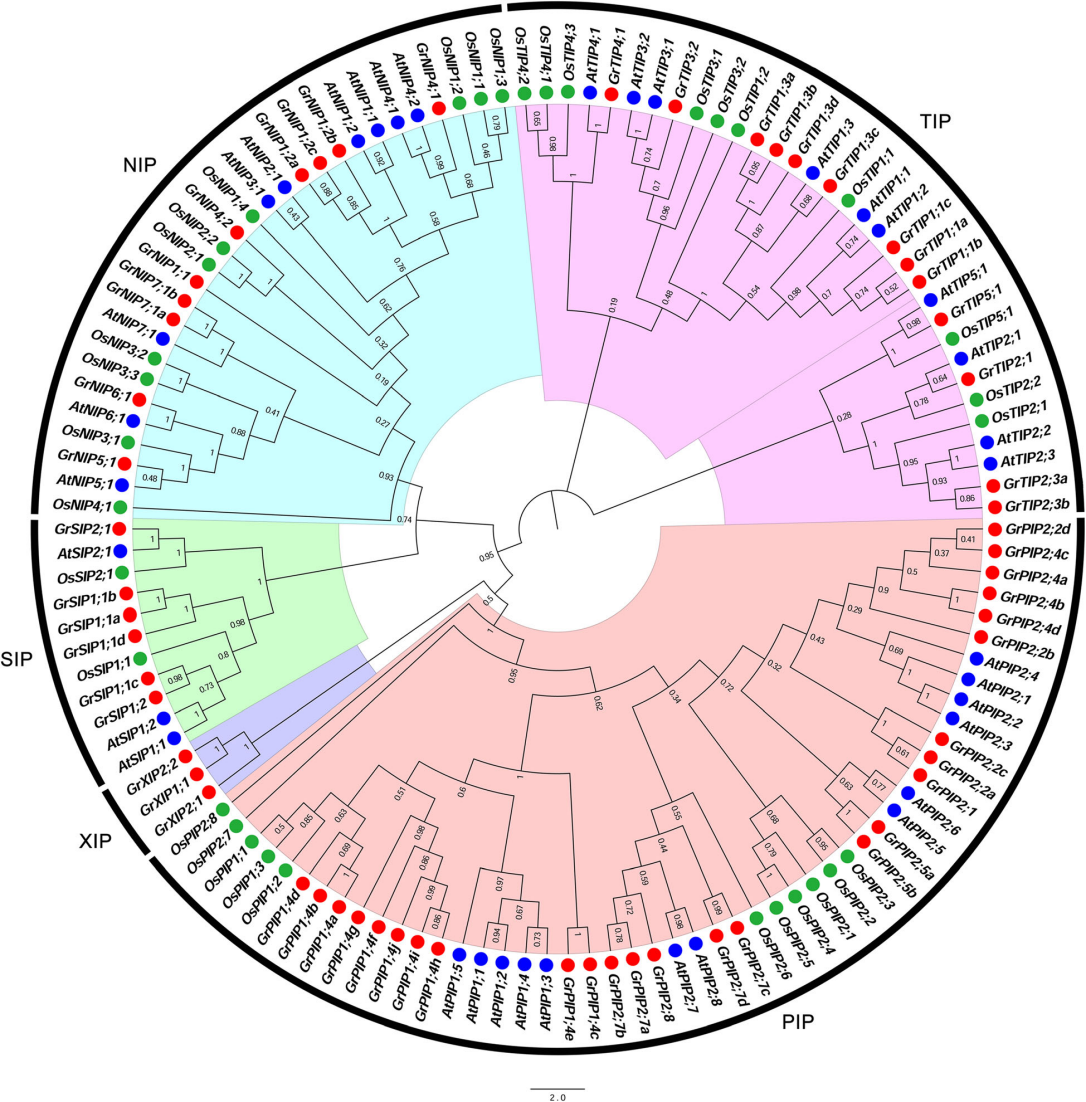
Phylogenetic relationships of AQPs from *G. raimondii* (Gr), *A. thaliana* (At) and *O. sativa* (Os). The rooted NJ tree was constructed using MEGA 7, and the bootstrap test was performed with 1000 replicates

To investigate the phylogenetic relationship of the AQP family in cotton, a total of 126 AQPs, 58 GrAQPs from *G. raimondii*, 35 AtAQPs from *A. thaliana* and 33 OsAQPs from *O. sativa*, were used to construct a Neighbour-Joining (N-J) phylogenetic tree with the MEGA 7.0 software [58]. As shown in Fig. 1, all AQPs were clustered into five subfamilies (PIP, NIP, SIP, TIP and XIP), and each group contained at least one member from the three species except group XIP was only from *G. raimondii*.

To reveal *GrAQP* segmental duplication events, three whole intra-genomic duplication data files of *G. raimondii*, *A. thaliana* and *O. sativa*, and two inter-genomic duplication data file between *G. raimondii* and two other species were downloaded from the PGDD database [59]. We detected that all AQP duplication events in the three species (Additional file 2: Figure S1 and Additional file 3: Table S2). In detail, 24 pairs of *GrAQPs* were segmental duplications within the *G. raimondii* genome, which involved 16 *GrAQPs*. All duplication pairs had Ka/Ks values less than 1, ranging from 0.023 to 0.685 (Additional file 3: Table S2), suggesting that the AQP gene family in *G. raimondii* had been subjected to purifying selection during the long-term evolutionary process.

AQP duplication patterns were further analyzed between *G. raimondii*, *A. thaliana* and *O. sativa*. Among 55 *GrAQPs* from 25 *AtAQP* orthologs, seven pairs of duplication events were identified between *G. raimondii* and *A. thaliana*, including *GrPIP1;4a*/*AtPIP1;5*, *GrPIP1;4d*/*AtPIP1;1* (*AtPIP1;2, AtPIP1;3* and *AtPIP1;4*), *GrSIP1;1c*/*AtSIP1;2* and *GrSIP1;2*/*AtSIP1;1* (Additional file 3: Table S2). However, no duplication was observed between *G. raimondii* and *O. sativa*, indicating the less conservation of AQPs between *Gossypium* and *O. sativa*.

### Structural characterization of AQPs unravels the evolutionary conservation in cotton

Taking *GrAQPs* as an example, we analyzed their exon/intron structures and transmembrane domains. The gene structures of 58 *GrAQPs* were analyzed by GSDS 2.041 [60], and displayed in Additional file 4: Figure S2. The number of introns of 58 *GrAQPs* varied from 0 to 4. In detail, 23 *GrAQPs* genes had three introns (21 *GrPIPs* and 2 *GrNIPs*); 10 *GrNIPs* had four introns; 19 *GrAQPs* had two introns (including 4 *GrPIPs*, 3 *GrSIPs*, 2 *GrXIPs* and 10 *GrTIPs*); 4 *GrAQPs* (3 *GrTIP1s* and *GrPIP2;4b*) and 4 *GrAQPs* (3 *GrSIP1s* and *GrXIP2;1*) had 1 or no intron, respectively. We found that most *GrPIPs* had 3 introns, most *GrNIPs* had 4 introns, and most *GrTIPs* had 2 introns, indicating the conserved distribution of introns in each subfamily.

**Fig. 2:**
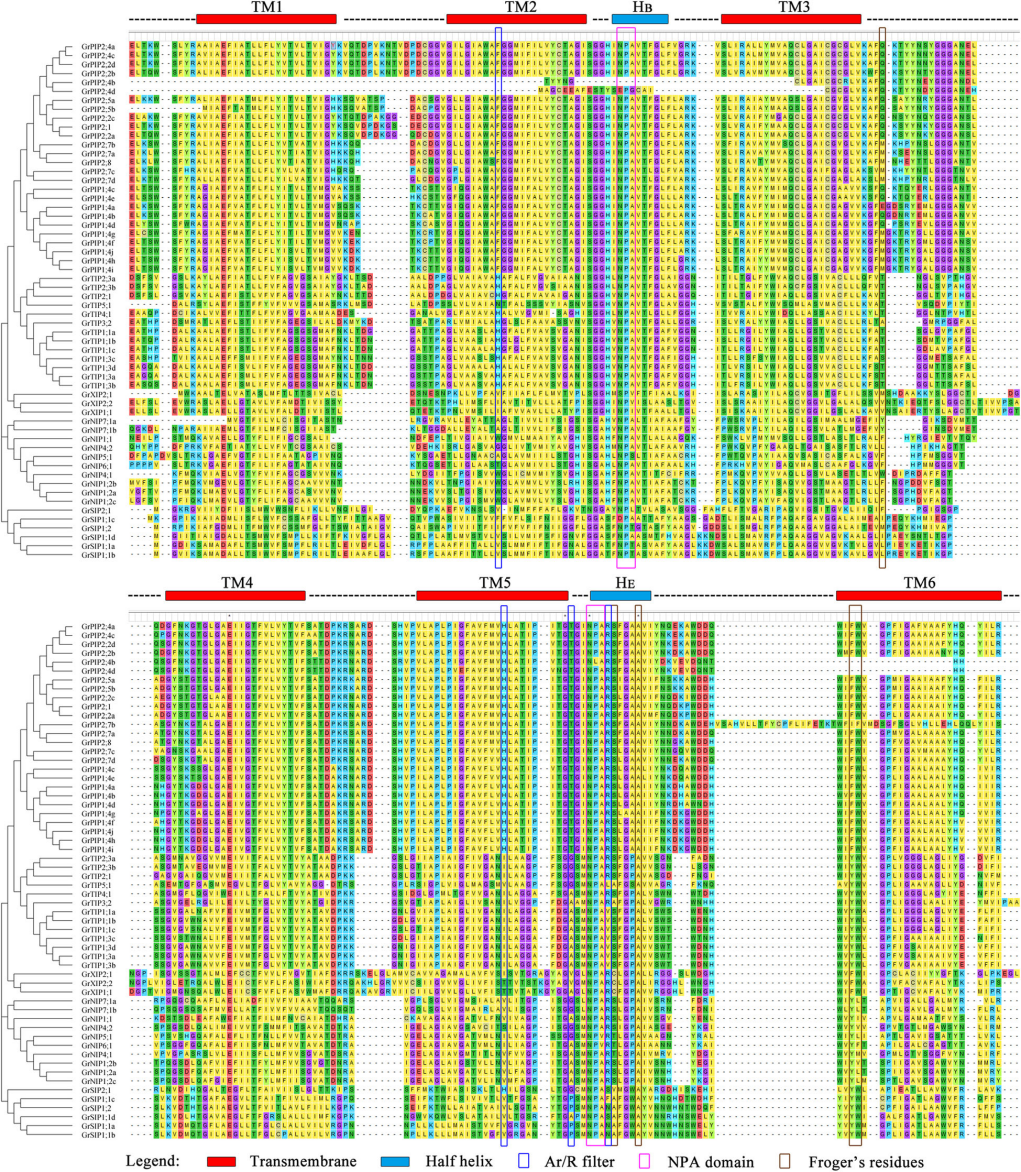
Protein sequence alignment of GrAQPs. Conserved transmembrane domains (TM1–6) and NPA motifs, ar/R selectivity filters, and Froger’s residues were identified in five AQP subfamilies (PIP, NIP, TIP, SIP and XIP) in *G. raimondii*

Protein domain analysis showed that each putative AQP protein contains a MIP domain (Additional file 4: Figure S2). Multiple alignment of all 58 GrAQPs showed the MIP domain structures in detail (Fig. 2). These GrAQPs displayed differences in the Asn-Pro-Ala (NPA) motif and residues at ar/R selectivity filters and Froger’s positions (Fig. 2 and Additional file 5: Table S3). Most AQPs contained two conserved NPA motifs, except for GrPIP2;4d, GrNIP6;1, GrSIP2;1, GrSIP1;1c, GrSIP1;2, GrSIP1;1a, GrSIP1;1b and three GrXIPs which were found to harbor a single NPA motif. Majority of members from PIP and TIP subfamilies showed the typical NPA motif except for GrPIP2;4d with Asparagine to Glutamic acid and Alanine to Glycine substitution in the first NPA motif and GrPIP2;4b with Proline to Leucine in the second NPA motif, respectively. In the NIP subfamily, the first NPA motif was found to be conserved in most members except for GrNIP5;1 with Alanine to Serine, while the second NPA motif showed Alanine to Valine substitution in GrNIP5;1 and GrNIP6;1. In SIP and XIP subfamilies, the first NPA motif showed substitution except for GrSIP1;1d, while the second NPA motif was conserved.

All PIP subfamily members showed a conserved ar/R filter residues with Phenylalanine in H2 (except for GrPIP2;7c and GrPIP2;7d with Valine), Histidine at H5, Threonine at LE1 and Arginine at LE2 (Fig. 2 and Additional file 5: Table S3). In the TIP subfamily, H2 and H5 positions of ar/R filter contained Histidine and Isoleucine, respectively, except for GrTIP5;1, where were Asparagine and Valine residue, respectively (Fig. 2 and Additional file 5: Table S3). LE1 and LE2 positions were specific for each group of GrTIPs. All GrTIP1s were characterized by Alanine (LE1) and Valine (LE2). GrTIP2s were characterized by Glycine (LE1) and Arginine (LE2). GrTIP3s and GrTIP4s were characterized by Alanine (LE1) and Arginine (LE2). In the NIP subfamily, GrNIP1s and GrNIP4;1 were characterized by Tryptophan (H2), Valine (H5), Alanine (LE1) and Arginine (LE2) whereas GrNIP4;2 were comprised of Glycine (H2), Serine (H5), Glycine (LE1) and Arginine (LE2). GrNIP5;1, GrNIP6;1 and GrNIP7s were comprised of Alanine/Threonine (H2), Isoleucine/Valine (H5), Glycine/Alanine (LE1) and Arginine (LE2). The SIP family members showed Valine/Isoleucine/Phenylalanine (LE1), Histidine/Valine (LE2), Proline/Glycine/Alanine (H2), Phenylalanine/Serine/Asparagine (H5) residues whereas the XIP subfamily members showed Isoleucine/Valine (H2), Isoleucine/Threonine (H5), Valine/Arginine (LE1), Arginine (LE2) (Fig. 2 and Additional file 5: Table S3). In summary, GrAQPs in the same subfamily generally presented similar protein structures.

### Comparative genomics among 30 species reveals the evolutionary conservation of AQP family in plants

**Fig. 3:**
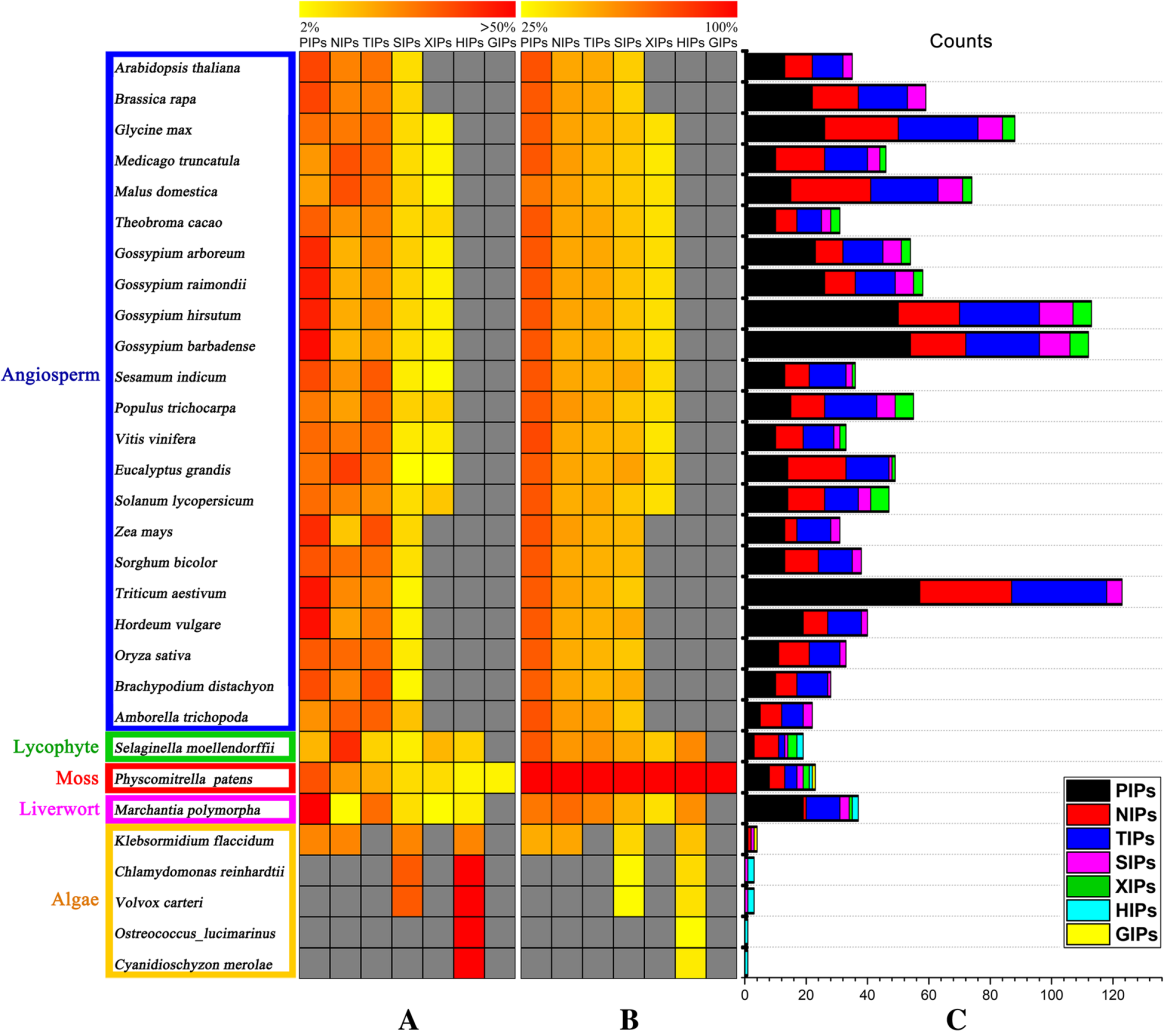
Percentage and similarity heat map of predicted AQPs in different species. **a**, **b** and **c**, the GENESIS simulation environment was used to estimate the percentage of AQP subfamilies (**a**) and the similarity among AQP subfamilies (**b**). The predicted AQPs were grouped into seven taxa (**c**). Consistent with previously reported results, candidate AQP genes were selected by BLASTP software searches that satisfied the criteria of E-value < 10^− 10^ and query coverage > 50%. Colored squares indicated the percentage from 2% (yellow) to > 50% (red) (**a**) and the sequence similarity from 25% (yellow) to 100% (red) (**b**). Clades on the left with different colors indicated: angiosperm (blue), lycophyte (green), moss (red), liverwort (pink), and algae (orange). Gray squares indicated that no proteins were found that satisfied the selection criteria (**a** and **b**)

To identify potential orthologs of aquaporins, we performed a bioinformatics analysis of predicted aquaporin genes across 30 species, involved in algae, liverwort, moss, lycophyte and angiosperm (Fig. 3). We found that there was an overall increase in the number of AQP gene families from algae to angiosperm using BLASTp with a selection criterion of E-value < 10^− 10^ and query coverage > 50% (Fig. 3), in consistent with the reported researches [34]. All AQPs were divided into seven subfamilies (PIP, NIP, TIP, SIP, XIP, HIP and GIP) in green plants (Fig. 3 and Additional file 6: Table S4). Among those 30 species, we found that *Physcomitrella patens* contained all seven subfamilies; the dicots had five subfamilies (PIP, TIP, NIP, SIP, and XIP); the monocots only had four (PIP, TIP, NIP and SIP). Whereas the number of subfamilies decreased during the evolution of land plants, the number of MIP isoforms in each species increased. Furthermore, the percentage heat map showed the highest proportion was PIPs in most angiosperm species and the second were NIPs and TIPs (Fig. 3a). The number of aquaporins in allotetraploid cotton was similar to allohexaploid wheat, but much higher than those model species, *Arabidopsis* and rice (Fig. 3c). Besides, we chose 23 AQPs in *Physcomitrella patens* as queries to calculate the average sequence similarity of AQP subfamily among 30 species (Fig. 3b and Additional file 7: Table S5). The similarity heat map demonstrated the sequences of PIPs were most highly conserved in most species except algae, then were NIPs and TIPs (Fig. 3b). So, the PIP subfamily may play a significant role in the long-term natural selection of plants by virtue of the highest quantitative distribution and most conserved sequence characteristic.

**Fig. 4:**
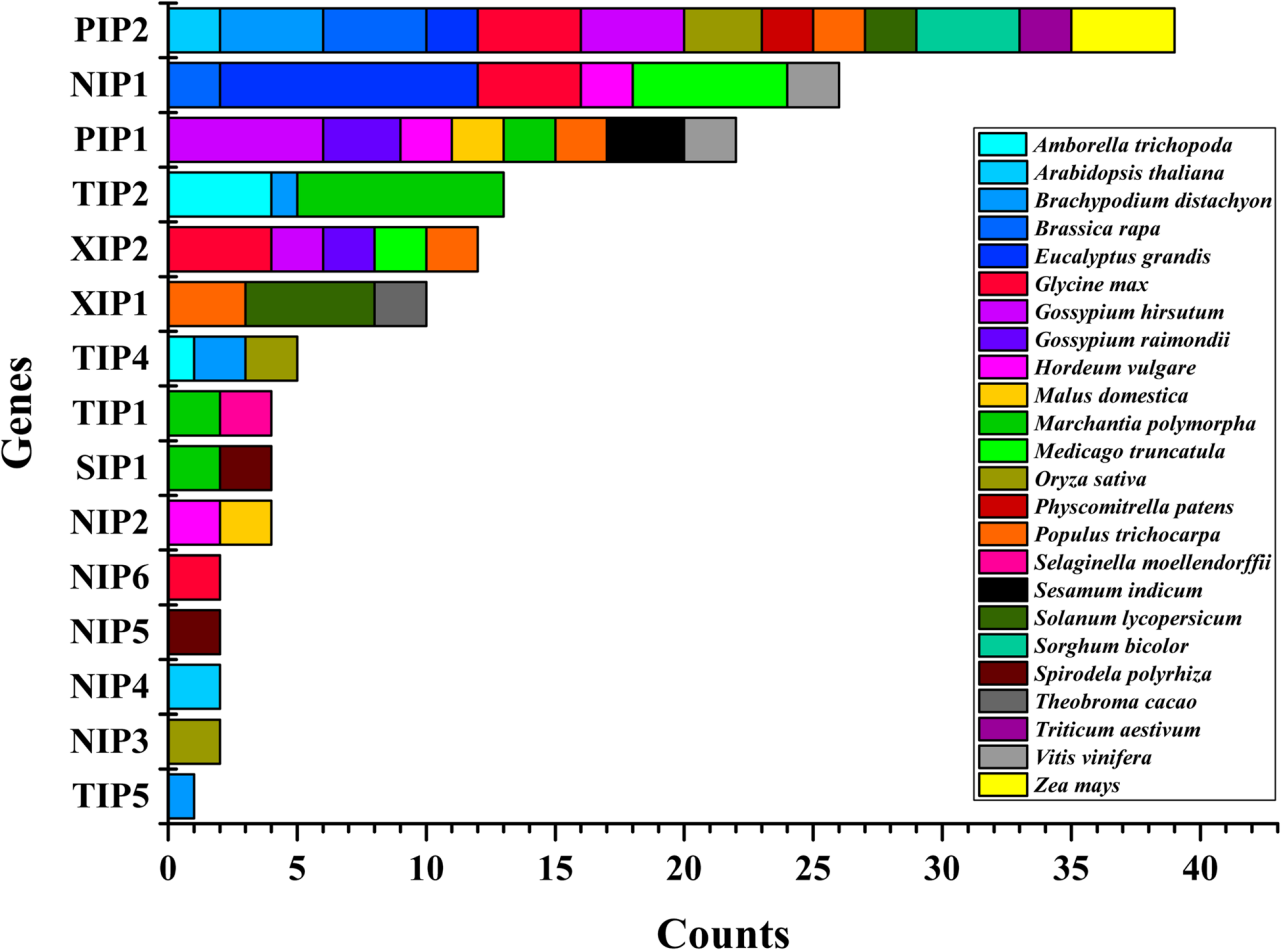
The amount of predicted AQP tandem repeat genes in different species. To reveal AQP tandem repeat events, the whole genome data files of 24 well-studied species were downloaded from the Phytozome database. 148 candidate AQP tandem repeat genes were selected by the criteria that genes separated by five or fewer genes within 100 Kb regions, and the similarity > 70% between two genes. Different colored squares indicated different species on the right. PIP1 and PIP2 represented PIP1a-f and PIP2a-d, respectively; NIP1-NIP6 represented NIP1a-j, NIP2a-b, NIP3a-b, NIP4a-b, NIP5a-b and NIP6a-b, respectively; TIP1, TIP2, TIP4 and TIP5 represented TIP1a-b, TIP2a-h, TIP4a-b and TIP5, respectively; SIP1 represented SIP1a-b; XIP1 and XIP2 represented XIP1a-e and XIP2a-d, respectively

According to the standard of tandem duplication events that genes separated by five or fewer genes within 100 Kb regions, and the similarity > 70% between two genes, we selected 148 candidate AQP tandem repeat genes across 24 well-studied species. The nomenclature of these 148 candidate AQPs was defined according to the PANTHER Database (http://www.pantherdb.org/). Different paralogs were tagged a-j. In total, the origin of these AQP genes were 39 in PIP2 group, 26 in NIP1, 22 in PIP1, 13 in TIP2, 12 in XIP2, ten in XIP1, five in TIP4, four each in TIP1, SIP1 and NIP2, two each in NIP3 to NIP 6 and one in TIP5 (Fig. 4 and Additional file 8: Table S6). Among them, PIP1s and PIP2s stem from 13 and eight representative species, respectively. Collectively, the PIP tandem repeat genes were distributed in 19 species except for *Amborella trichopoda*, *Medicago truncatula*, *Selaginella moellendorffii*, *Spirodela polyrhiza* and *Theobroma cacao*. We further used the 148 predicted protein sequences from 24 species for phylogenetic analysis. The rooted NJ tree showed all of the AQP tandem repeat genes were clustered into five subfamilies (PIP, NIP, SIP, TIP and XIP), and each group contained at least two species (Additional file 9: Figure S3). Additionally, we found that a group of AQP tandem repeat orthologs were distributed both in cotton (*GrPIP1a-c*, *GhPIP1a-c* and *GhPIP1d-e*) and sesame (*SiPIP1a-c*). While, another pair of AQP tandem repeat orthologs, *GhPIP2a*/*GhPIP2b* and *GhPIP2c*/*GhPIP2d* in *Gossypium hirsutum* were clustered with *PtPIP2a* and *PtPIP2b* in *Populus trichocarpa*.

### Comparative transcriptomics between *G. raimondii*, *G. davidsonii* and *G. hirsutum* reveals functional diversity of AQP family in cotton

**Fig. 5:**
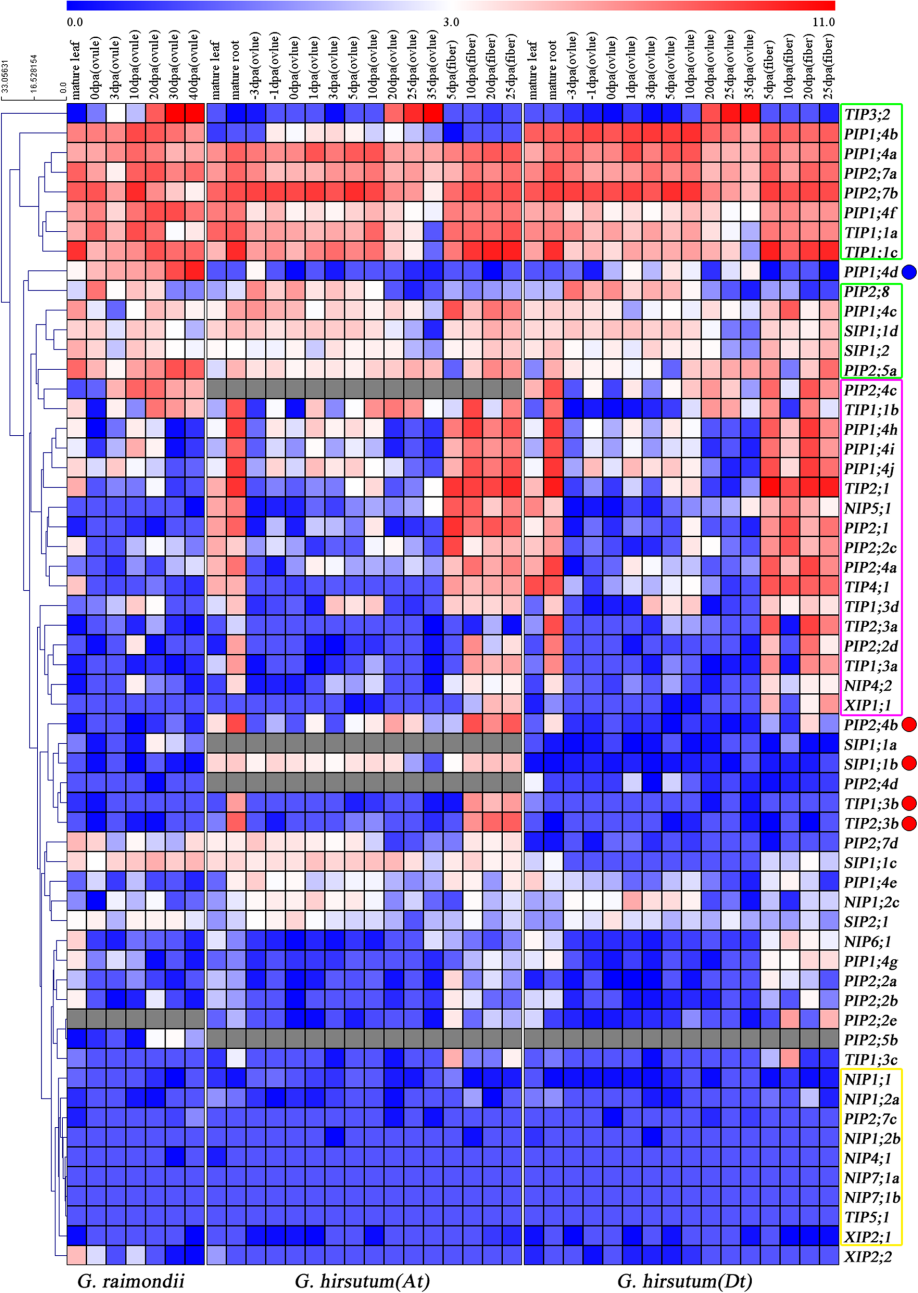
Expression patterns of AQP genes in *G. raimondii* and *G. hirsutum*. Seven tissues and organs were involved in mature leaf, 0, 3, 10, 20, 30 and 40 DPA ovules in *G. raimondii*. Sixteen tissues and organs were involved in root, leaf, − 3, − 1, 0, 1, 3, 5, 10, 20, 25 or 35 DPA ovules, 5, 10, 20 and 25 DPA fibers in *G. hirsutum* acc. TM-1. The color represented AQPs expression levels normalized by Log_2_ (FPKM+ 1). Colored squares indicated expression levels from 0 (blue) to 11 (red). Gray squares indicated that no gene was found in that species. The clustered tree of hierarchical clustering model was showed on the left

In order to understand the putative functions of AQP genes, we analyzed the expression profiles of all identified 58 and 113 AQPs by using RNA-seq data of *G. raimondii* and *G. hirsutum* acc. TM-1 respectively, including 18 different tissues and organs (Fig. 5). Eleven AQP genes in the green box were highly expressed in all tissues and *TIP3;2* was specially highly expressed in 20–40 DPA ovules in both *G. raimondii* and *G. hirsutum*. Four AQP genes marked with red dot were only highly expressed in *G. hirsutum*. Meanwhile, *GrPIP1;4d* was only highly expressed in *G. raimondii*. However, several AQP genes in the yellow box were lowly expressed or not detectable in all tested tissues in the two cotton species. In addition, 17 *GhAQPs* in the pink box were highly expressed in the root and fiber of *G. hirsutum*.

**Fig. 6:**
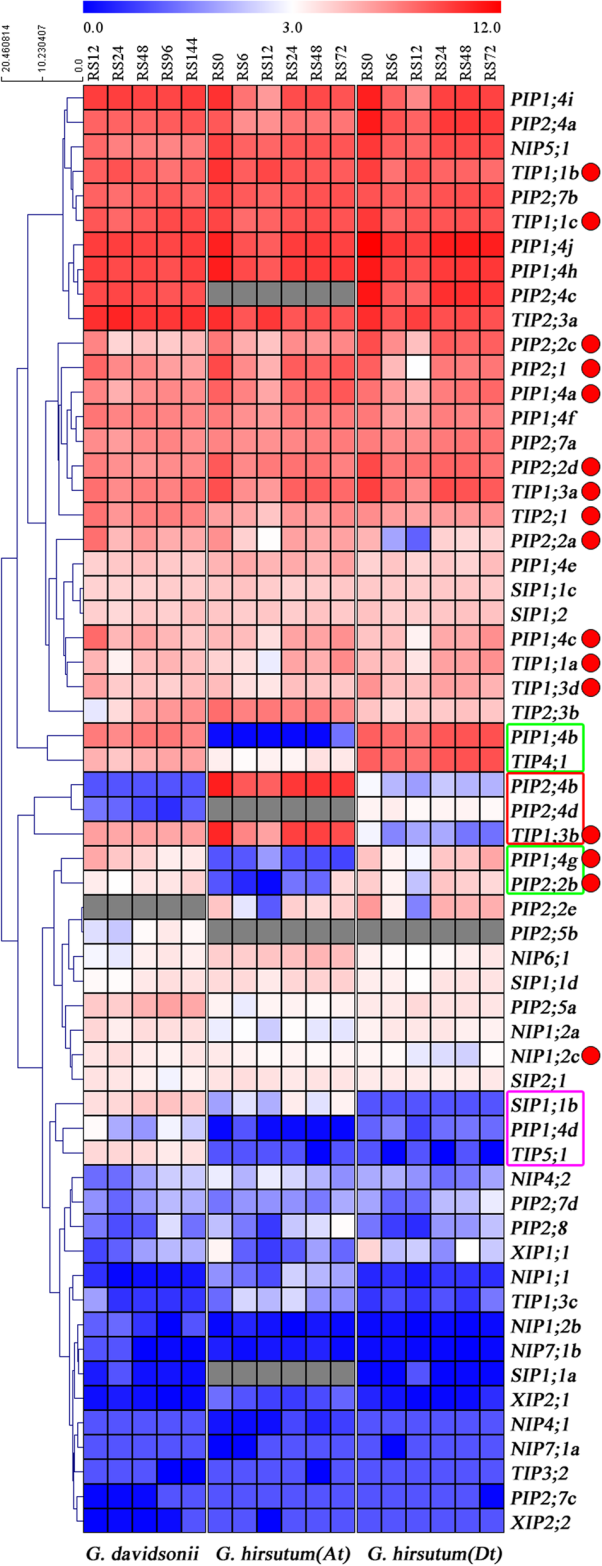
Expression patterns of AQP genes under salt stresses in *G. davidsonii* and *G. hirsutum*. A heat map of expression was generated using transcriptome data of roots under salt stress and control conditions at 12 h (RS12), 24 h (RS24), 48 h (RS48), 96 h (RS96) and 144 h (RS144) in *G. davidsonii*. Six time points of salt stress were 0 h (CK-0 h), 6 h (NaCl-6 h), 12 h (NaCl-12 h), 24 h (NaCl-24 h), 48 h (NaCl-48 h) and 72 h (NaCl-72 h) in *G. hirsutum* acc. TM-1. The color represented AQPs expression levels normalized by Log_2_ (FPKM+ 1). Colored squares indicated expression levels from 0 (blue) to 11 or 12 (red). Gray squares indicated that no gene was found in that species. The clustered tree of hierarchical clustering model was showed on the left

To investigate the potential functions of AQP genes in response to stress, we detected the gene expression profiles under salt stress conditions by comparing transcriptome data between *G. davidsonii*, a diploid D genome wild salinity-tolerant cotton species and *G. hirsutum* acc. TM-1 (Fig. 6). We found that ten *AQP* genes had different expression profiles in the two cotton species. Four genes in the green box were highly expressed in *G. davidsonii* and D-subgenome of *G. hirsutum* but lowly expressed in A-subgenome of *G. hirsutum*. Three AQP genes in the red box showed the opposite expression profiles between these two cotton species. Three genes in the pink box were specially expressed in *G. davidsonii* compared with *G. hirsutum*. These results indicated that cotton AQP gene family may play an important role in response to salt stress, and cotton orthologous genes from different cotton species existed functional differentiation.

**Fig. 7:**
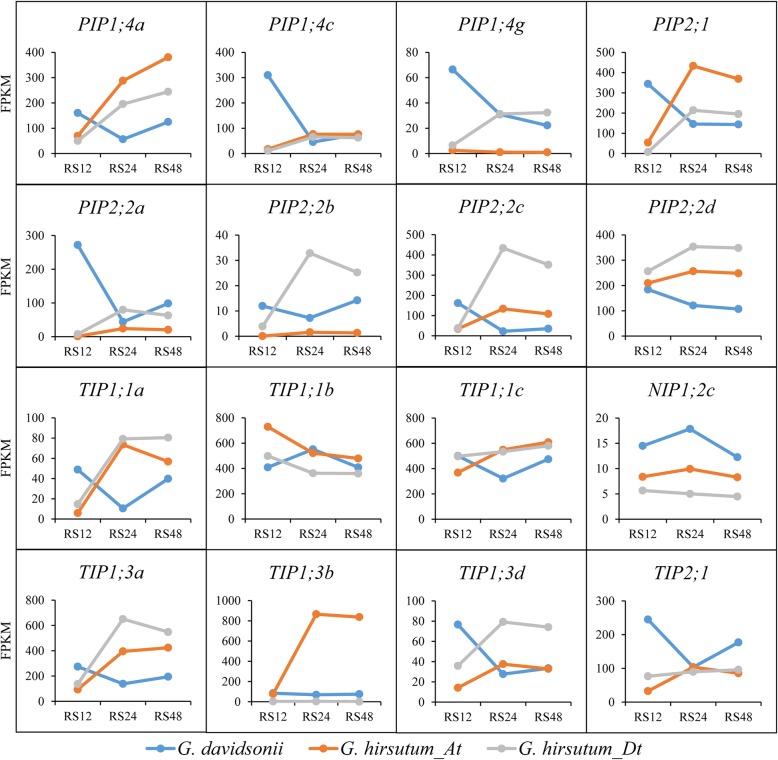
Expression patterns of AQP genes under salt stress in roots of *G. davidsonii* and *G. hirsutum*. Line charts of sixteen commonly induced orthologs were drawn using the transcriptome data of salt stress in both *G. davidsonii* and *G. hirsutum*. Three time points after salt treatment were 12 h (R12), 24 h (R24) and 48 h (R48) and R represented the root

Under salt stress, 26 and 55 AQP genes were induced in the root of *G. davidsonii* and *G. hirsutum*, respectively (Fig. 6). Among them, the expression profiles of 18 orthologs in both *G. davidsonii* (named as *Gd*) and *G. hirsutum* were further analyzed. Except for *NIP4;2* and *TIP1;3c* at a low expression level (FPKM < 5), the remaining 16 orthologs marked with red dot were selected to compare the expression between these two cotton species under salt stress (Fig. 7). The expression of *GhAQPs* increased rapidly under salt stress from 12 h to 24 h and reached a high level at 24 h except for *GhTIP1;1b_At(Dt)*, suggesting that these genes might be involved in stress responses. However, the expression of *GdAQPs* decreased under salt stress from 12 h to 24 h and fell to a low level at 24 h, then the expression level of *GdAQPs* increased gradually with the extension of salt treatment time except for *GdTIP1;1b* and *GdNIP1;2c*. Interestingly, the expression of most *GhAQPs* was increasing at relatively early stages (12 h and 24 h), but slightly decreasing from 24 h to 48 h after salt treatment. However, most *GdAQPs* displayed decreasing expression profiles from 12 h to 24 h but gradually increasing expressions after salt treatment 24 h, indicating the differentially induced response in *G. davidsonii* and *G. hirsutum*.

To verify the expression profiles derived from the transcriptome data for *GdAQPs* and *GhAQPs* under salt stress, we selected eight AQP genes for qRT-PCR analysis (Additional file 10: Figure S4). The expression patterns of *PIP2;2c*, *PIP2;5a*, *TIP2;1* and *SIP1;2* were down-regulated under salt treatment in both two cotton species. However, several AQP genes (*PIP1;4c*, *NIP1;2c*, *TIP1;1c* and *PIP2;4a*) showed differential expression level in different cotton species under salt stress. The expression levels of *PIP1;4c* and *NIP1;2c* at 48 h were up-regulated in *G. davidsonii* while down-regulated in *G. hirsutum*; *TIP1;1c* was down-regulated at 48 h in *G. davidsonii* but up-regulated in *G. hirsutum*. In addition, *PIP2;4a* displayed the increased expression in *G. hirsutum* but was not affected in *G. davidsonii* under salt stress. The qRT-PCR results further confirmed the difference of induced response between *G. hirsutum* and *G. davidsonii* under salt stress. Taken together, AQP genes play important roles in response to salt stress and some may experience functional differentiation during the process of evolution and domestication.

## Discussion

Aquaporins play the important roles in plant growth and developmental process. The number of AQPs varied widely among different species. In recent decades, genome-wide identification of AQP gene family has been reported in many plant species. For instance, there are 35, 33, 66, 28, 47, 31 and 59 AQPs in *Arabidopsis* [15], rice [32], soybean [37], grape [33], tomato [36], maize [14] and Chinese cabbage [41], respectively. Due to the limitations of both EST databases and an available genomic sequence in the past years, the identification of AQP gene family had been reported in *Gossypium hirsutum* [56], however, the number of reported AQPs was incomplete. In this study, we finished the genome-wide identification of AQP genes and systematically investigated the characterization in four sequenced cotton species. We also summarized and compared the results from different genome databases with the same accession, especially in tandem replication events. For instance, the difference of AQPs among the three genomes of *G. hirsutum* acc. TM-1 released from different organizations was mainly concentrated in several duplicate genes (*GhPIP1;4 h/i/j* and *GhPIP2;2a/e*). We further analyzed these genes by extracting and aligning their DNA sequences to verify their existence in *G. hirsutum* (Additional file 11: Figure S5 and Additional file 12: Figure S6). This difference might be caused by incomplete gene annotations, and we selected an overlap of them as candidate *GhAQPs* for further analysis. Totally, 53, 58, 113 and 111 AQP genes in *G. arboreum*, *G. raimondii*, *G. hirsutum* and *G. barbadense* were identified, respectively (Additional file 1: Table S1). We found that the number of *GaAQPs* or *GrAQPs* were similar to *B. rapa*, and there was a classical polyploidization phenomenon that the number of AQPs in *G. hirsutum* or *G. barbadense* was twice that of the two diploid cotton species. Partial undetected orthologs in the four cotton species were possibly due to the incomplete genomic sequence or the gene deletion during tetraploidization process of cotton.

### The evolutionary characteristics of aquaporin family in plants

AQPs were detected in all 30 species from algae to angiosperm, and the size of AQP gene families in 30 species was expanding during the long-term evolutionary selection (Fig. 3c). There was a significant expansion of the AQP families in cotton compared with the AQPs of other higher plants, which may result from genome duplications and genome size. Furthermore, polyploidization of angiosperms contributed many quantities of AQPs, such as the high proportion in cotton and wheat (Fig. 3a). We found that there were about the same amount of AQPs in allohexaploid wheat and allotetraploid cotton. That may explain why wheat is relatively salt-sensitive crop plant in comparison to cotton which have the ability to complete their life cycle in a salt ion-rich environment [61].

Because of their polyploidization, plants have developed multiple AQPs [1, 62]. However, only CAQPs are diversified in higher plants [63]. For example, 35 AQPs were detected in *Arabidopsis thaliana*, and further subdivided into four groups: 13 PIPs, 10 TIPs, 9 NIPs, and 3 SIPs (Additional file 6: Table S4). Another subfamily of CAQPs, XIP, was identified in poplar trees, cotton, soybean, apple, cacao, sesame, grape, tomato and lucerne but absent in *Arabidopsis*, turnip, rice, wheat, barley, maize, sorghum and *Brachypodium distachyon* [14, 15, 32–37, 39, 41, 64] (Fig. 3). Thus, XIPs were detected in the dicots but absent in the monocots and the cruciferae. XIPs had been reported to function in the transport of permeant substrates including glycerol across the plasma membrane in specific plant tissues [65]. Lately, HIP (similar to PIP and TIP) and LIP (similar to SIP), two new subfamilies also originating from CAQP, have been discovered in algae but not in higher plants [17, 18]. Coincidentally, GIP (an AQGP) has been found in *Physcomitrella patens* and another closely related primitive moss, functioning as a glycerol channel [12].

It is special for plant AQPs to separate into seven subfamilies (PIP, TIP, NIP, SIP, XIP, HIP and LIP) according to their primary sequences, so plant AQPs may be comparable with animal AQPs in function. For instance, the extra functions of NIPs compensate for the absence of AQGP in plants [19, 20]. Similarly, the absence of SAQP might be recovered by SIP, XIP or HIP. Taken together, phylogenetic verification and functional evolution consistent with primary sequence changes of plant AQPs will give novel insights into understanding structure-function relationships of AQPs.

### The structural conservation of AQPs in plants

Phylogenetic analysis of AQP proteins in *Arabidopsis* showed that the 35 members of the AQP family were divided into four structurally homologous subfamilies: PIP, TIP, NIP, and SIP [15]. These four subfamilies can be further classified into eight groups based on comparisons of the narrow selectivity filter regions (the aromatic/Arg [ar/R] filter) [66]. Here, we also clustered 58 AQPs in *G. raimondii* as nine groups according to their ar/R selectivity filter. The PIP subfamily consisting of 26 members in *G. raimondii* were grouped into PIP Group. The TIP subfamily was the second largest in AQP family of *G. raimondii*, consisting of 13 full-length genes, and showed the most diversity within putative pore regions with three different ar/R subgroups: TIP Group I (GrTIP1s), TIP Group IIa (GrTIP2s) and TIP Group IIb (GrTIP3;2 and GrTIP4;1), and TIP Group III (a single member GrTIP5;1). Six GrNIPs (GrNIP1s, GrNIP4;1 and GrNIP4;2) were designated NIP Group I, whereas four (GrNIP5;1, GrNIP6;1, GrNIP7;1a and GrNIP7;1b) were designated NIP Group II. The SIP subfamily was divided into SIP Group I (GrSIP1s) and SIP Group II (GrSIP2;1), respectively. The recently reported XIP subfamily was distinguished as XIP Group (Additional file 5: Table S3). By comparing with *Arabidopsis*, the various AQPs of *G. raimondii* were also separated into eight distinct ar/R groups except XIPs. Some of them adhered to the classical aquaporin structures, and others were completely divergent and would likely have functions distinct from CAQPs and AQGPs.

In general, the selectivity of the permeant substrates of AQPs is determined by the hydrophobicity and size of the narrow selectivity filter regions [67–69]. Most PIP family members in cotton contained hydrophilic ar/R selectivity filter (F/H/T/R) (Fig. 2 and Additional file 5: Table S3), which was also observed in PIP family of AQPs from other plant species such as *A. thaliana*, *B. rapa*, *G. max*, *L. usitatissimum*, *P. vulgaris* and *R. communis* [15, 37, 41, 70–72]. PIPs play a crucial role in water transport promoting water absorption of roots and leaves [73]. Except for water transport, PIPs are also known to boost the diffusion of CO_2_ to affect photosynthesis in mesophyll tissue of *A. thaliana* and *N. tabacum* [74, 75]. Our expression analysis also showed the abundant expression of *PIPs* (*PIP1;4a, PIP1;4f, PIP2;7a* and *PIP2;7b*) in cotton roots, leaves as well as fibers (Fig. 5), suggesting a similar role of PIPs in water transport and CO_2_ diffusion in cotton. Among GrTIPs, GrTIP1s were found to have residues (H/I/A/V) forming more hydrophobic ar/R filter compared to GrTIP2s and GrTIP3;2/GrTIP4;1 which contained ar/R filter with H/I/G/R and H/I/A/R residues, respectively. The residues in ar/R selectivity filter of GrTIPs were similar to TIPs from other plant species. Previous experiments have proved that TIPs were mainly located in the vacuolar membrane and acted as functional small solutes transporters such as NH_4_^+^, H_2_O_2_ and urea [2, 4, 76]. Among the NIPs, GrNIP1s and GrNIP4;1 were found to be more hydrophobic (WVAR) compared to GrNIP7s (AVGR) and GrNIP6;1 (TIAR). In plants, the ability to absorb silicon depends on the existence of NIPs containing the GSGR selectivity filter [77, 78]. GrNIP5;1 and GrNIP4;2 belong to the NIP Group II and Group I, respectively. They differed in the ar/R selectivity filter of the H2 and H5 positions. The amino acids of the GrNIP4;2 ar/R filter consisted of glycine, serine, glycine, and arginine (GSGR), compared with alanine, isoleucine, glycine, and arginine (AIGR) in GrNIP5;1 (Fig. 2 and Additional file 5: Table S3). Different studies reported the variations at H5 position in the ar/R selective filter of XIP family [12, 39]. In plants, the ar/R selectivity filters of XIPs are more hydrophobic, which is attributed to the enhancement of hydrophobicity with Valine/Isoleucine at H5 position. Here, Threonine/Isoleucine occupied H5 position of the three GrXIPs. The hydrophobicity of XIPs contributes to the transport of small neutral molecules such as glycerol, urea, and boric acid in plants [65].

### The functional diversity of AQPs in plants

Aquaporins (AQPs) function in a huge variety of processes in the whole plant life. To date, there are many expression and functional studies of AQP genes in cotton. Based on existing results, PIPs and TIPs are involved in fiber elongation, leaf and root development and in response to drought and cold stress [79–83]. Interestingly, some reported AQP genes are conserved in structure but diverse in function. For example, *GhTIP1;3d_At* and *GhTIP1;1c_Dt*, two members of cotton TIP1 type subfamily, shared higher sequence similarity both at nucleotide and amino acid levels. But they exhibited completely different expression patterns in different tissues and developmental stages. *GhTIP1;3d_At* was preferentially expressed in 5 to 15 DPA fiber [83], whereas *GhTIP1;1c_Dt* mainly accumulated in roots and hypocotyls [81]. Furthermore, the expression of *GhTIP1;1c_Dt* varied with the root development, showing high expression levels in young roots and then gradually declined to low levels in mature roots [81]. Such different expression patterns indicated that these two genes might participate in different physiological processes. Through integrating the bioinformatics analysis with biological experiment validation, key AQPs responsible for plant development and stress response could be further explored in cotton.

More recent duplication events give rise to closer isoforms in a single species. It might be a way to control specific expression according to developmental and environmental conditions [84]. For instance, five cotton paralogs, *GhPIP2;7a_At/Dt*, *GhPIP2;7b_At/Dt* and *GhPIP2;7d_Dt*, showed different expression patterns in cotton. *GhPIP2;7a_At/Dt* and *GhPIP2;7b_At/Dt* were the primary aquaporin genes in fibers. They regulated their activities by selectively forming hetero-oligomers to meet the demands for rapid fiber elongation [79]. However, *GhPIP2;7d_Dt* mainly accumulated in cotyledons and leaves, and responded to drought stress [80]. These results suggested that cotton paralogous genes probably experienced the functional differentiation during the evolutionary process.

It is widely considered that having two and more genes may increase expression level due to a gene dosage effect of the duplicated genes [21]. A recent study also reported that frequent gene duplications were significant to the evolution of a species based on the genome-wide analysis of different organisms [85]. Due to its large size, the AQP gene family in plants is well suited to test such a phylogenetic hypothesis. Hence, we found that a group of AQP tandem repeat genes were distributed both in cotton (*GrPIP1a-c*, *GhPIP1a-c* and *GhPIP1d-e*) and sesame (*SiPIP1a-c*). Wu et al. (2016) [64] reported that there were a series of tandem duplication genes, *SiPIP1;5*, *SiPIP1;6* and *SiPIP1;7* (*SiPIP1a-c*), with the high sequence similarity in sesame. Under the *Ralstonia solanacearum* infection, the expression of *SiPIP1;7* was up-regulated while *SiPIP1;5* and *SiPIP1;6* had no significant changes [64]. In our study, *GhPIP1;4h_At/GhPIP1;4i_At/GhPIP1;4j_At* and *GhPIP1;4h_Dt/GhPIP1;4i_Dt/GhPIP1;4j_Dt* (*GhPIP1a-c* and *GhPIP1d-e*) were identified and predominantly expressed in roots and fibers in *G. hirsutum*. This group of homologous tandem repeat genes appeared functional differentiation with highly similar sequence structure at amino acid level in cotton and sesame. While, another pair of AQP tandem repeat genes, *GhPIP2;2a_At/GhPIP2;2e_At* and *GhPIP2;2a_Dt/GhPIP2;2e_Dt* (*GhPIP2a*/*GhPIP2b* and *GhPIP2c*/*GhPIP2d*) in *G. hirsutum* were clustered with *PtPIP2;5* and *PtPIP2;6* (*PtPIP2a* and *PtPIP2b*) in *Populus trichocarpa*, which were reported to be highly expressed in roots of poplar trees [39]. Conversely, the expression of *GhPIP2;2a_At/GhPIP2;2e_At* and *GhPIP2;2a_Dt/GhPIP2;2e_Dt* in our research were low expressed in roots of *G. hirsutum*. Taken together, the AQP tandem duplication events showed the structural conservation but the functional diversity both in intra-species or inter-species.

## Conclusions

Herein, 53, 58, 113 and 111 AQP genes were identified in *G. arboreum*, *G. raimondii*, *G. hirsutum* and *G. barbadense*, respectively. The analysis of exon/intron structure, functional domains, phylogenetic relationships and gene duplications showed the conserved evolution of cotton AQPs. Structural conservation was further verified using 30 AQP families and 148 AQP tandem repeat genes across different species from algae to angiosperm. Most AQPs exhibited tissue-specific expression both in *G. raimondii* and *G. hirsutum*, and a bias of time to peak expression between *G. davidsonii* and *G. hirsutum* under salt stress. These novel results revealed the structural conservation and functional diversity of AQPs in different cotton species. This is a comprehensive analysis of AQP gene family in cotton, which will provide an overall and useful reference for elucidating the genetic mechanism and breeding utilization of AQP genes in the future.

## Methods

### Identification and annotation of AQPs in different cotton species

The sequences of four sequenced cotton species, *G. raimondii* [55], *G. arboreum* [53], *G. hirsutum* acc. TM-1 from three different organizations [50–52], and *G. barbadense* acc. 3–79 and cv. Hai7124 [51, 52], were all downloaded from the CottonGen database [86]. There were three genomic databases of *G. hirsutum* acc. TM-1 with different sources, that from Zhang et al. (2015) named as NAU, Wang et al. (2019) as HAU and Hu et al. (2019) as ZJU, respectively. The 35 AQP genes of *Arabidopsis* were employed as queries to identify putative orthologs in four cotton species using BLASTp with the e-value <1e^− 10^. The results from different sources of genome databases were summarized and compared to determine the final number of AQPs. The candidate AQPs were annotated using criteria established in Johanson et al. [15] and according to the closest orthologs in *A. thaliana*. Briefly, the AQP family was defined as the identity of amino acid sequences exhibiting > 40% to other previously identified AQP sequences. Moreover, a protein subfamily was defined as the identity of sequences showing > 60% while the identity exhibiting < 40% were described as a new AQP protein family. The MIP domain (PF00230) for aquaporins was downloaded from Pfam [87], and it was employed to identify all possible AQP genes in four cotton species using HMMER [57] (v3.1b2) with the e-value <1e^− 10^. Each candidate AQP gene was further confirmed by SMART [88] and InterPro [89]. The theoretical pI (isoelectric point), MW (molecular weight) and GRAVY (Grand average of hydropathicity) of the AQPs were investigated using Expasy [90].

### Evolutionary bioinformatics analysis of AQPs

Except for four cotton species, the other genome sequences were obtained for *Klebsormidium flaccidum* from the 1000 Plants database (http://http://www.onekp.com), *Cyanidioschyzon merolae* from the NCBI Genome database (https://www.ncbi.nlm.nih.gov/genome/), *Sesamum indicum* from the Sinbase (http://www.sesamegenome.org/index.php), and other 23 species from the Phytozome database (https://phytozome.jgi.doe.gov/pz/portal.html), respectively. Candidate protein sequences, consistent with reported researches, were selected by using the software BLASTP with a selection criterion of E-value < 10^− 10^ and query coverage > 50%. The AQP tandem repeat genes from 24 well-studied plant species were selected according to the standard that genes separated by five or fewer genes within 100 Kb regions and the similarity > 70% between two genes.

### Phylogenetic and synteny analysis of cotton AQPs

The phylogenetic tree was constructed using the Muscle alignment and the Neighbor-Joining (NJ) method with 1000 bootstrap replicates of MEGA 7.0 software [58]. The whole intra- and inter-genomic duplication files of *G. raimondii*, *A. thaliana* and *O. sativa* were downloaded from the PGDD [59], and the visualization was carried out using the CIRCOS tool [91]. The ratios (Ka/Ks) of the nonsynonymous substitution rate (Ka) and the synonymous substitution rate (Ks) were used to assess the selection pressure for duplication genes.

### Gene structure and conserved motif analysis

The exon/intron structures of *GrAQPs* and *GhAQPs* were drawn using GSDS 2.041 [60], according to inputted gene GFF files. The HMMER web server (2015) [92] was employed to identify conserved domains of GrAQPs. In addition, NPA motifs and transmembrane domains were presented alone using multiple alignment software MEGA version 7.0 [58].

### RNA-seq data collection and analysis

To analyze the expression patterns of AQP genes, the published transcriptome data for *G. hirsutum* acc. TM-1 were downloaded from the NCBI SRA database under accession code PRJNA248163. We used RNA-seq data of *G. raimondii*, including mature leaf, 0, 3 DPA ovules (Accessions: SRP009820) and 10–40 DPA ovules (Accession: SRP017168), and RNA-seq data from *G. davidsonii* (Accessions: SRP061663), which were collected from roots at 12, 24, 48, 96, and 144 h after salt stress treatment (200 mM NaCl), with corresponding controls under normal condition. The corresponding RNA samples were stored for further analysis.

For obtaining RNA-seq data after salt stress treatment from *G. hirsutum* acc. TM-1, seedlings of *G. hirsutum* acc. TM-1 were grown in a controlled environment chamber under the condition: 16 h light/8 h dark cycle at 28 °C. At the two true leaves and one heart shaped leaf stage, the seedlings were subjected to salt stress treatment. The cotton seedlings were transferred to nutrient solutions supplemented with 200 mM NaCl. Each treatment included 24 cotton seedlings and repeated three times. Roots were collected from the seedlings at 0, 6, 12, 24, 48 and 72 h post-treatment. Well-watered plants served as controls. For each biological replicate, the roots were collected from three individual seedlings. The cotton root samples were immediately frozen in liquid nitrogen and stored at − 70 °C for RNA isolation and expression analysis. RNA sequencing was performed on an Illumina HiSeq 2000 system. The RNA-seq data have been deposited in NCBI database under BioProject accession PRJNA532694.

The expression levels of AQP genes were calculated using Log_2_ (FPKM+ 1) (FPKM, fragments per kilobase of transcript per million fragments mapped). Expression patterns were visualized by Mev4.9.0 (https://sourceforge.net/projects/mev-tm4/), and clustered by the hierarchical clustering model.

### RNA isolation and qRT-PCR analysis

RNA was extracted from cotton roots using a BioFlux kit (TransGen Biotec Co., Ltd.). First-strand cDNA was generated using TransScript One-Step gDNA Removal and cDNA Synthesis SuperMix (TransGen Biotec Co., Ltd.) according to the manufacturer’s instructions. The qRT-PCR assay was performed in a 7500 Real-Time PCR System (Applied Biosystems) using First Start Universal SYBR Green Master (Roche). The cotton *Histone3* gene (Accession No: AF024716) was used as an internal control, and the relative expression levels of the genes were calculated using the comparative threshold cycle method. The qRT-PCR procedures were set as follows: (1) 95 °C, 10 min; (2) 40 cycles of 95 °C for 15 s, 60 °C for 30 s and 72 °C for 30 s; (3) a melting curve analysis from 65 to 95 °C (1 s hold per 0.2 °C increase) to check the specificity of the amplified product. The relative expression level was calculated with the 2^−△△CT^ method [93]. The qRT-PCR primers were listed in Additional file 13: Table S7.

All generated data in this study were repeated at least three times on three biological replicates. The relative expression levels of stressed samples were compared to those of the controls (well-watered). The statistical significance was determined by the parametric one-way ANOVA test.

## Additional files

Additional file 1: **Table S1.** Characterization of AQP family genes identified in *Gossypium*. (XLSX 24 kb)

Additional file 2: **Figure S1.** Distribution of duplications of AQP genes in *G. raimondii*, *A. thaliana* and *O. sativa*. The outer ring represented chromosomes with different colors in different species and the inner links represented intra- and inter-genomic duplications among these three species. At: *A. thaliana*; Os: *O. sativa*; Gr: *G. raimondii*. (TIFF 370 kb)

Additional file 3: **Table S2.** Intra- or inter-genome duplications of AQP genes in *G. raimondii*, *A. thaliana* and *O. sativa*. (DOCX 18 kb)

Additional file 4: **Figure S2.** Gene structures and protein domains of *GrAQPs*. The phylogenetic relationship was showed on the left (**A**). The exon/intron distribution of *GrAQP* genes was showed in the middle (**B**). Exons and introns were represented by yellow boxes and lines, respectively. Based on their protein sequences, the MIP domain were detected by HMMSCAN in all *GrAQPs* (**C**). (TIFF 4596 kb)

Additional file 5: **Table S3.** Conserved motifs, selectivity filter and amino acid residues of AQPs in *G. raimondii*. (DOCX 20 kb)

Additional file 6: **Table S4.** Amount of AQP isoforms in 30 species from algae to angiosperm based on E-value < e^− 10^ and query coverage > 50%. (DOCX 18 kb)

Additional file 7: **Table S5.** Similarity analysis for the evolution of AQP isoforms in 30 species from algae to angiosperm based on E-value < e^− 10^ and query coverage > 50%. (DOCX 17 kb)

Additional file 8: **Table S6.** Overview of AQP tandem repeat genes in green plants. (DOCX 26 kb)

Additional file 9: **Figure S3.** Phylogenetic relationships of AQP tandem repeat genes from 24 well-studied species. The rooted NJ tree was constructed using MEGA 7, and the bootstrap test was performed with 1000 replicates. Am: *A. trichopoda*; At: *A. thaliana*; Bd: *B. distachyon*; Br: *B. rapa*; Eg: *E. grandis*; Gh: *G. hirsutum*; Gm: *G. max*; Gr: *G. raimondii*; Hv: *H. vulgare*; Md: *M. domestica*; Mp: *M. polymorpha*; Mt.: *M. truncatula*; Os: *O. sativa*; Pp: *P. patens*; Pt: *P. trichocarpa*; Sb: *S. bicolor*; Si: *S. indicum*; Sl: *S. lycopersicum*; Sm: *S. moellendorffii*; Sp: *S. polyrhiza*; Ta: *T. aestivum*; Tc: *T. cacao*; Vv: *V. vinifera*; Zm: *Z. mays*. (TIFF 9311 kb)

Additional file 10: **Figure S4.** Column charts of qRT-PCR results of the AQP genes in roots of *G. davidsonii* and *G. hirsutum* L. acc. TM-1 at 48 h post-treatment with salt stress. At the two true leaves and one heart shaped leaf stage, cotton seedlings were treated with 200 mM NaCl. The cotton *Histone3* gene was used as an internal control. Values presented are means of three independent experiments, with error bars indicating standard deviations. (TIFF 448 kb)

Additional file 11: **Figure S5.** Sequences alignment of *GhPIP1;4h_At*, *GhPIP1;4i_At* and *GhPIP1;4j_At* in three genome databases of *G. hirsutum* acc. TM-1. The DNA sequences of *GhPIP1;4h_At*, *GhPIP1;4i_At* and *GhPIP1;4j_At* were extracted from three released genome databases of *G. hirsutum* acc. TM-1, that from Zhang et al. (2015) named as NAU, Wang et al. (2019) as HAU and Hu et al. (2019) as ZJU, respectively (**B**). The location of genes on chromosomes was shown on the left (**A**) and the corresponding gene IDs were listed on the right with unknown or undivided genes marked in red (**C**). (PDF 71 kb)

Additional file 12: **Figure S6.** Sequences alignment of *GhPIP2;2a_At*, *GhPIP2;2e_At*, *GhPIP2;2a_Dt* and *GhPIP2;2e_Dt* in three genome databases of *G. hirsutum* acc. TM-1. The DNA sequences of *GhPIP2;2a_At*, *GhPIP2;2e_At*, *GhPIP2;2a_Dt* and *GhPIP2;2e_Dt* were extracted from three released genome databases of *G. hirsutum* acc. TM-1, that from Zhang et al. (2015) named as NAU, Wang et al. (2019) as HAU and Hu et al. (2019) as ZJU, respectively (**B**). The location of genes on chromosomes was shown on the left (**A**) and the corresponding gene IDs were listed on the right with unknown or undivided genes marked in red (**C**). (PDF 71 kb)

Additional file 13: **Table S7.** Primers information for quantitative real-time PCR analysis. (DOCX 14 kb)

## Data Availability

The genomic database of four cotton species, *G. raimondii*, *G. arboreum*, *G. hirsutum* acc. TM-1 with three different sources, and *G. barbadense* acc. 3–79 and cv. Hai7124, were all downloaded from the CottonGen database (https://www.cottongen.org/data/download). The other genome sequence data were obtained for *Klebsormidium flaccidum* from the 1000 Plants database (http://http://www.onekp.com), *Cyanidioschyzon merolae* from the NCBI Genome database (https://www.ncbi.nlm.nih.gov/genome/), *Sesamum indicum* from the Sinbase (http://www.sesamegenome.org/index.php) and other 23 species from the Phytozome database (https://phytozome.jgi.doe.gov/pz/portal.html), respectively. RNA-seq data in this study have been deposited at the National Center of Biotechnology Information (NCBI, http://www.ncbi.nlm.nih.gov/) under the accessions PRJNA248163, PRJNA532694, SRP009820, SRP017168 and SRP061663.

## Abbreviations

AQGP: Aquaglyceroporin
AQP: Aquaporin
BLAST: Basic local alignment search tool
CAQP: Classical aquaporin
DPA: Days post anthesis
FPKM: Fragments per kilobase per million reads
GIP: GlpF-like intrinsic protein
HIP: Hybrid intrinsic protein
LIP: Large intrinsic protein
MIP: Major intrinsic protein
NIP: Nodulin26-like intrinsic protein
PIP: Plasma membrane intrinsic protein
qRT-PCR: Quantitative real-time PCR
SAQP: Superaquaporin
SIP: Small basic intrinsic protein
TIP: Tonoplast intrinsic protein
XIP: Uncategorized X intrinsic protein

## References

[1] Adams KL, Wendel JF (2005). Polyploidy and genome evolution in plants. Curr Opin Plant Biol.

[2] Liu LH, Ludewig U, Gassert B, Frommer WB, von Wiren N (2003). Urea transport by nitrogen-regulated tonoplast intrinsic proteins in *Arabidopsis*. Plant Physiol.

[3] Uehlein N, Lovisolo C, Siefritz F, Kaldenhoff R (2003). The tobacco aquaporin NtAQP1 is a membrane CO_2_ pore with physiological functions. Nature.

[4] Bienert GP, Moller AL, Kristiansen KA, Schulz A, Moller IM, Schjoerring JK (2007). Specific aquaporins facilitate the diffusion of hydrogen peroxide across membranes. J Biol Chem.

[5] Jahn TP, Moller AL, Zeuthen T, Holm LM, Klaerke DA, Mohsin B (2004). Aquaporin homologues in plants and mammals transport ammonia. FEBS Lett.

[6] Mukhopadhyay R, Bhattacharjee H, Rosen BP (2014). Aquaglyceroporins: generalized metalloid channels. BBA-General Subjects..

[7] Hara-Chikuma M, Verkman AS (2006). Physiological roles of glycerol-transporting aquaporins: the aquaglyceroporins. Cell Mol Life Sci CMLS.

[8] Rojek A, Praetorius J, Frokiaer J, Nielsen S, Fenton RA (2008). A current view of the mammalian aquaglyceroporins. Annu Rev Physiol.

[9] Benga G (2012). On the definition, nomenclature and classification of water channel proteins (aquaporins and relatives). Mol Asp Med.

[10] Yakata K, Hiroaki Y, Ishibashi K, Sohara E, Sasaki S, Mitsuoka K (2007). Aquaporin-11 containing a divergent NPA motif has normal water channel activity. BBA-Biomembranes.

[11] Gupta AB, Verma RK, Agarwal V, Vajpai M, Bansal V, Sankararamakrishnan R (2012). MIPModDB: a central resource for the superfamily of major intrinsic proteins. Nucleic Acids Res.

[12] Danielson JA, Johanson U (2008). Unexpected complexity of the aquaporin gene family in the moss *Physcomitrella patens*. BMC Plant Biol.

[13] Gustavsson S, Lebrun AS, Norden K, Chaumont F, Johanson U (2005). A novel plant major intrinsic protein in *Physcomitrella patens* most similar to bacterial glycerol channels. Plant Physiol.

[14] Chaumont F, Barrieu F, Wojcik E, Chrispeels MJ, Jung R (2001). Aquaporins constitute a large and highly divergent protein family in maize. Plant Physiol.

[15] Johanson U, Karlsson M, Johansson I, Gustavsson S, Sjovall S, Fraysse L (2001). The complete set of genes encoding major intrinsic proteins in *Arabidopsis* provides a framework for a new nomenclature for major intrinsic proteins in plants. Plant Physiol.

[16] Kaldenhoff R, Fischer M (2006). Functional aquaporin diversity in plants. BBA-Biomembranes.

[17] Danielson JA, Johanson U (2010). Phylogeny of major intrinsic proteins. Adv Exp Med Biol.

[18] Khabudaev KV, Petrova DP, Grachev MA, Likhoshway YV (2014). A new subfamily LIP of the major intrinsic proteins. BMC Genomics.

[19] Pommerrenig B, Diehn TA, Bienert GP (2015). Metalloido-porins: essentiality of Nodulin 26-like intrinsic proteins in metalloid transport. Plant Sci.

[20] Takano J, Wada M, Ludewig U, Schaaf G, von Wiren N, Fujiwara T (2006). The *Arabidopsis* major intrinsic protein NIP5;1 is essential for efficient boron uptake and plant development under boron limitation. Plant Cell.

[21] Flagel LE, Wendel JF (2009). Gene duplication and evolutionary novelty in plants. New Phytol.

[22] Panchy N, Lehti-Shiu M, Shiu SH (2016). Evolution of gene duplication in plants. Plant Physiol.

[23] Freeling M (2009). Bias in plant gene content following different sorts of duplication: tandem, whole-genome, segmental, or by transposition. Annu Rev Plant Biol.

[24] Hahn MW (2009). Distinguishing among evolutionary models for the maintenance of gene duplicates. J Hered.

[25] Moghe GD, Shiu SH (2014). The causes and molecular consequences of polyploidy in flowering plants. Ann N Y Acad Sci.

[26] Michael TP, VanBuren R (2015). Progress, challenges and the future of crop genomes. Curr Opin Plant Biol.

[27] Wendel JF (2015). The wondrous cycles of polyploidy in plants. Am J Bot.

[28] Salman-Minkov A, Sabath N, Mayrose I (2016). Whole-genome duplication as a key factor in crop domestication. Nat Plants.

[29] Wang Y, Wang X, Tang H, Tan X, Ficklin SP, Feltus FA (2011). Modes of gene duplication contribute differently to genetic novelty and redundancy, but show parallels across divergent angiosperms. PLoS One.

[30] Konno N, Hyodo S, Yamaguchi Y, Matsuda K, Uchiyama M (2010). Vasotocin/V2-type receptor/aquaporin axis exists in African lungfish kidney but is functional only in terrestrial condition. Endocrinology.

[31] Martos-Sitcha JA, Campinho MA, Mancera JM, Martinez-Rodriguez G, Fuentes J (2015). Vasotocin and isotocin regulate aquaporin 1 function in the sea bream. J Exp Biol.

[32] Sakurai J, Ishikawa F, Yamaguchi T, Uemura M, Maeshima M (2005). Identification of 33 rice aquaporin genes and analysis of their expression and function. Plant Cell Physiol.

[33] Fouquet R, Léon C, Ollat N, Barrieu F (2008). Identification of grapevine aquaporins and expression analysis in developing berries. Plant Cell Rep.

[34] Azad AK, Ahmed J, Alum MA, Hasan MM, Ishikawa T, Sawa Y (2016). Genome-wide characterization of major intrinsic proteins in four grass plants and their non-aqua transport selectivity profiles with comparative perspective. PLoS One.

[35] Hove RM, Ziemann M, Bhave M (2015). Identification and expression analysis of the barley (*Hordeum vulgare L.*) aquaporin gene family. PLoS One.

[36] Reuscher S, Akiyama M, Mori C, Aoki K, Shibata D, Shiratake K (2013). Genome-wide identification and expression analysis of aquaporins in tomato. PLoS One.

[37] Zhang DY, Ali Z, Wang CB, Xu L, Yi JX, Xu ZL (2013). Genome-wide sequence characterization and expression analysis of major intrinsic proteins in soybean (*Glycine max L.*). PLoS One.

[38] Anderberg HI, Kjellbom P, Johanson U (2012). Annotation of *Selaginella moellendorffii* major intrinsic proteins and the evolution of the protein family in terrestrial plants. Front Plant Sci.

[39] Gupta AB, Sankararamakrishnan R (2009). Genome-wide analysis of major intrinsic proteins in the tree plant *Populus trichocarpa*: characterization of XIP subfamily of aquaporins from evolutionary perspective. BMC Plant Biol.

[40] Shivaraj SM, Deshmukh RK, Rai R, Belanger R, Agrawal PK, Dash PK (2017). Genome-wide identification, characterization, and expression profile of aquaporin gene family in flax (*Linum usitatissimum*). Sci Rep.

[41] Kayum MA, Park JI, Nath UK, Biswas MK, Kim HT, Nou IS (2017). Genome-wide expression profiling of aquaporin genes confer responses to abiotic and biotic stresses in *Brassica rapa*. BMC Plant Biol.

[42] Ishibashi K, Morishita Y, Tanaka Y (2017). The evolutionary aspects of aquaporin family. Adv Exp Med Biol.

[43] Soto G, Alleva K, Amodeo G, Muschietti J, Ayub ND (2012). New insight into the evolution of aquaporins from flowering plants and vertebrates: orthologous identification and functional transfer is possible. Gene.

[44] Zardoya R, Villalba S (2001). A phylogenetic framework for the aquaporin family in eukaryotes. J Mol Evol.

[45] Wendel JF (1989). New World tetraploid cottons contain Old World cytoplasm. Proc Natl Acad Sci USA.

[46] Chen Z, Nie H, Grover CE, Wang Y, Li P, Wang M (2017). Entire nucleotide sequences of *Gossypium raimondii* and *G. arboreum* mitochondrial genomes revealed A-genome species as cytoplasmic donor of the allotetraploid species. Plant Biol (Stuttgart, Germany).

[47] Phillips LL, Clement D (1967). Variation in the diploid *Gossypium* species of Baja California. Madroño.

[48] Alvarez I, Wendel JF (2006). Cryptic interspecific introgression and genetic differentiation within *Gossypium aridum* (Malvaceae) and its relatives. Evolution.

[49] Wendel JF, Percival AE (1990). Molecular divergence in the Galapagos Islands—Baja California species pair, *Gossypium klotzschianum* and *G. davidsonii* (Malvaceae). Plant Syst Evol.

[50] Zhang T, Hu Y, Jiang W, Fang L, Guan X, Chen J (2015). Sequencing of allotetraploid cotton (*Gossypium hirsutum* L. acc. TM-1) provides a resource for fiber improvement. Nat Biotechnol.

[51] Wang M, Tu L, Yuan D, Zhu D, Shen C, Li J (2019). Reference genome sequences of two cultivated allotetraploid cottons, *Gossypium hirsutum* and *Gossypium barbadense*. Nat Genet.

[52] Hu Y, Chen J, Fang L, Zhang Z, Ma W, Niu Y (2019). *Gossypium barbadense* and *Gossypium hirsutum* genomes provide insights into the origin and evolution of allotetraploid cotton. Nat Genet.

[53] Du X, Huang G, He S, Yang Z, Sun G, Ma X (2018). Resequencing of 243 diploid cotton accessions based on an updated a genome identifies the genetic basis of key agronomic traits. Nat Genet.

[54] Paterson AH, Wendel JF, Gundlach H, Guo H, Jenkins J, Jin D (2012). Repeated polyploidization of *Gossypium* genomes and the evolution of spinnable cotton fibres. Nature.

[55] Wang K, Wang Z, Li F, Ye W, Wang J, Song G (2012). The draft genome of a diploid cotton *Gossypium raimondii*. Nat Genet.

[56] Park W, Scheffler BE, Bauer PJ, Campbell BT (2010). Identification of the family of aquaporin genes and their expression in upland cotton (*Gossypium hirsutum* L.). BMC Plant Biol.

[57] Finn RD, Clements J, Eddy SR (2011). HMMER web server: interactive sequence similarity searching. Nucleic Acids Res.

[58] Kumar S, Stecher G, Tamura K (2016). MEGA7: molecular evolutionary genetics analysis version 7.0 for bigger datasets. Mol Biol Evol.

[59] Lee TH, Tang H, Wang X, Paterson AH (2013). PGDD: a database of gene and genome duplication in plants. Nucleic Acids Res.

[60] Hu B, Jin J, Guo AY, Zhang H, Luo J, Gao G (2015). GSDS 2.0: an upgraded gene feature visualization server. Bioinformatics.

[61] Zorb C, Geilfus CM, Dietz KJ (2018). Salinity and crop yield. Plant Biol.

[62] Meyers LA, Levin DA (2006). On the abundance of polyploids in flowering plants. Evolution.

[63] Li G, Santoni V, Maurel C (2014). Plant aquaporins: roles in plant physiol. BBA-General Subjects.

[64] Wu XY, Cheng CZ, Lv GQ, Wang XY (2016). Identification and characterization of the AQP gene family in sesame. Sci Agr Sin.

[65] Bienert GP, Bienert MD, Jahn TP, Boutry M, Chaumont F (2011). Solanaceae XIPs are plasma membrane aquaporins that facilitate the transport of many uncharged substrates. Plant J.

[66] Wallace IS, Roberts DM (2004). Homology modeling of representative subfamilies of *Arabidopsis* major intrinsic proteins. Classification based on the aromatic/arginine selectivity filter. Plant Physiol.

[67] Tornroth-Horsefield S, Wang Y, Hedfalk K, Johanson U, Karlsson M, Tajkhorshid E (2006). Structural mechanism of plant aquaporin gating. Nature.

[68] Sui H, Han BG, Lee JK, Walian P, Jap BK (2001). Structural basis of water-specific transport through the AQP1 water channel. Nature.

[69] Fu D, Libson A, Miercke LJ, Weitzman C, Nollert P, Krucinski J (2000). Structure of a glycerol-conducting channel and the basis for its selectivity. Science.

[70] Deshmukh RK, Vivancos J, Guerin V, Sonah H, Labbe C, Belzile F (2013). Identification and functional characterization of silicon transporters in soybean using comparative genomics of major intrinsic proteins in *Arabidopsis* and rice. Plant Mol Biol.

[71] Zou Z, Gong J, Huang Q, Mo Y, Yang L, Xie G (2015). Gene structures, evolution, classification and expression profiles of the aquaporin gene family in castor bean (*Ricinus communis* L.). PLoS One.

[72] Ariani A, Gepts P (2015). Genome-wide identification and characterization of aquaporin gene family in common bean (*Phaseolus vulgaris* L.). Mol Gen Genomics.

[73] Maurel C, Verdoucq L, Luu DT, Santoni V (2008). Plant aquaporins: membrane channels with multiple integrated functions. Annu Rev Plant Biol.

[74] Flexas J, Ribas-Carbo M, Hanson DT, Bota J, Otto B, Cifre J (2006). Tobacco aquaporin NtAQP1 is involved in mesophyll conductance to CO_2_ in vivo. Plant J.

[75] Heckwolf M, Pater D, Hanson DT, Kaldenhoff R (2011). The *Arabidopsis thaliana* aquaporin AtPIP1;2 is a physiologically relevant CO_2_ transport facilitator. Plant J.

[76] Holm LM, Jahn TP, Moller AL, Schjoerring JK, Ferri D, Klaerke DA (2005). NH_3_ and NH^4+^ permeability in aquaporin-expressing Xenopus oocytes. Pflug Arch Eur J Phy.

[77] Deshmukh RK, Vivancos J, Ramakrishnan G, Guerin V, Carpentier G, Sonah H (2015). A precise spacing between the NPA domains of aquaporins is essential for silicon permeability in plants. Plant J.

[78] Ma JF, Tamai K, Yamaji N, Mitani N, Konishi S, Katsuhara M (2006). A silicon transporter in rice. Nature.

[79] Li DD, Ruan XM, Zhang J, Wu YJ, Wang XL, Li XB (2013). Cotton plasma membrane intrinsic protein 2s (PIP2s) selectively interact to regulate their water channel activities and are required for fibre development. New Phytol.

[80] Zhang J, Li D, Zou D, Luo F, Wang X, Zheng Y (2013). A cotton gene encoding a plasma membrane aquaporin is involved in seedling development and in response to drought stress. Acta Biochim Biophys Sin.

[81] Li DD, Tai FJ, Zhang ZT, Li Y, Zheng Y, Wu YF (2009). A cotton gene encodes a tonoplast aquaporin that is involved in cell tolerance to cold stress. Gene.

[82] Li DD, Wu YJ, Ruan XM, Li B, Zhu L, Wang H (2009). Expressions of three cotton genes encoding the PIP proteins are regulated in root development and in response to stresses. Plant Cell Rep.

[83] Liu D, Tu L, Wang L, Li Y, Zhu L, Zhang X (2008). Characterization and expression of plasma and tonoplast membrane aquaporins in elongating cotton fibers. Plant Cell Rep.

[84] Wistow GJ, Pisano MM, Chepelinsky AB (1991). Tandem sequence repeats in transmembrane channel proteins. Trends Biochem Sci.

[85] Lynch M, Conery JS (2000). The evolutionary fate and consequences of duplicate genes. Science.

[86] Yu J, Jung S, Cheng CH, Ficklin SP, Lee T, Zheng P (2014). CottonGen: a genomics, genetics and breeding database for cotton research. Nucleic Acids Res.

[87] Finn RD, Bateman A, Clements J, Coggill P, Eberhardt RY, Eddy SR (2014). Pfam: the protein families database. Nucleic Acids Res.

[88] Letunic I, Doerks T, Bork P (2015). SMART: recent updates, new developments and status in 2015. Nucleic Acids Res.

[89] Jones P, Binns D, Chang HY, Fraser M, Li W, McAnulla C (2014). InterProScan 5: genome-scale protein function classification. Bioinformatics.

[90] Artimo P, Jonnalagedda M, Arnold K, Baratin D, Csardi G, de Castro E (2012). ExPASy: SIB bioinformatics resource portal. Nucleic Acids Res.

[91] Krzywinski M, Schein J, Birol I, Connors J, Gascoyne R, Horsman D (2009). Circos: an information aesthetic for comparative genomics. Genome Res.

[92] Finn RD, Clements J, Arndt W, Miller BL, Wheeler TJ, Schreiber F (2015). HMMER web server: 2015 update. Nucleic Acids Res.

[93] Livak KJ, Schmittgen TD (2001). Analysis of relative gene expression data using real-time quantitative PCR and the 2(−Delta Delta C(T)) method. Methods.

