# Supplementary figures and images for: Combining genome-wide and transcriptome-wide analyses reveal the evolutionary conservation and functional diversity of aquaporins in cotton
Source: BMC Genomics. 2019 Jul 1;20:538. doi: 10.1186/s12864-019-5928-2 (PMC6604486; doi:10.1186/s12864-019-5928-2)

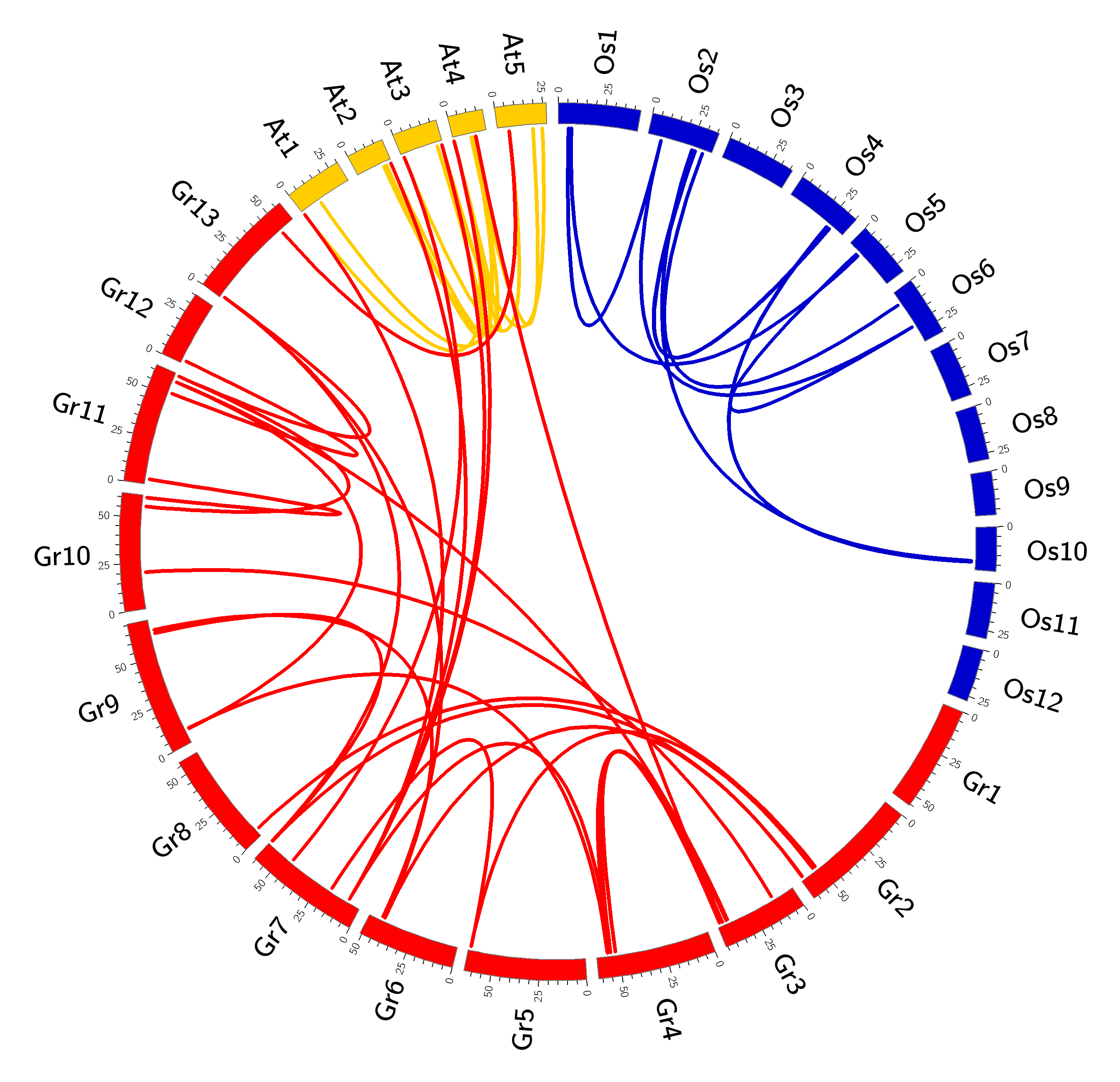

Supplement: Supplementary file 2 — Figure S1. Distribution of duplications of AQP genes in G. raimondii, A. thaliana and O. sativa. The outer ring represented chromosomes with different colors in different species and the inner links represented intra- and inter-genomic duplications among these three species. At: A. thaliana; Os: O. sativa; Gr: G. raimondii. (TIFF 370 kb) [file 12864_2019_5928_MOESM2_ESM.tiff]

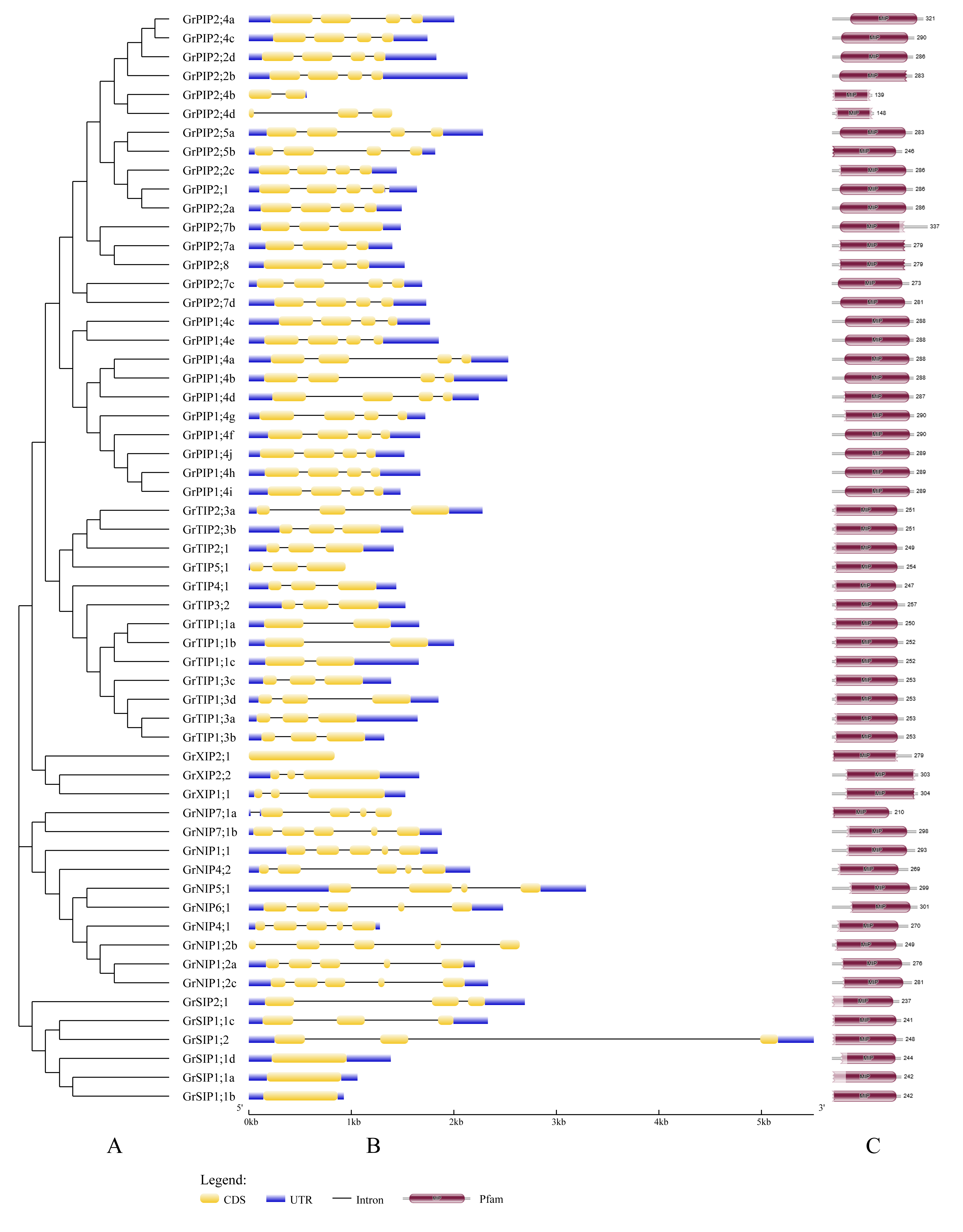

Supplement: Supplementary file 4 — Figure S2. Gene structures and protein domains of GrAQPs. The phylogenetic relationship was showed on the left (A). The exon/intron distribution of GrAQP genes was showed in the middle (B). Exons and introns were represented by yellow boxes and lines, respectively. Based on their protein sequences, the MIP domain were detected by HMMSCAN in all GrAQPs (C). (TIFF 4596 kb) [file 12864_2019_5928_MOESM4_ESM.tiff]

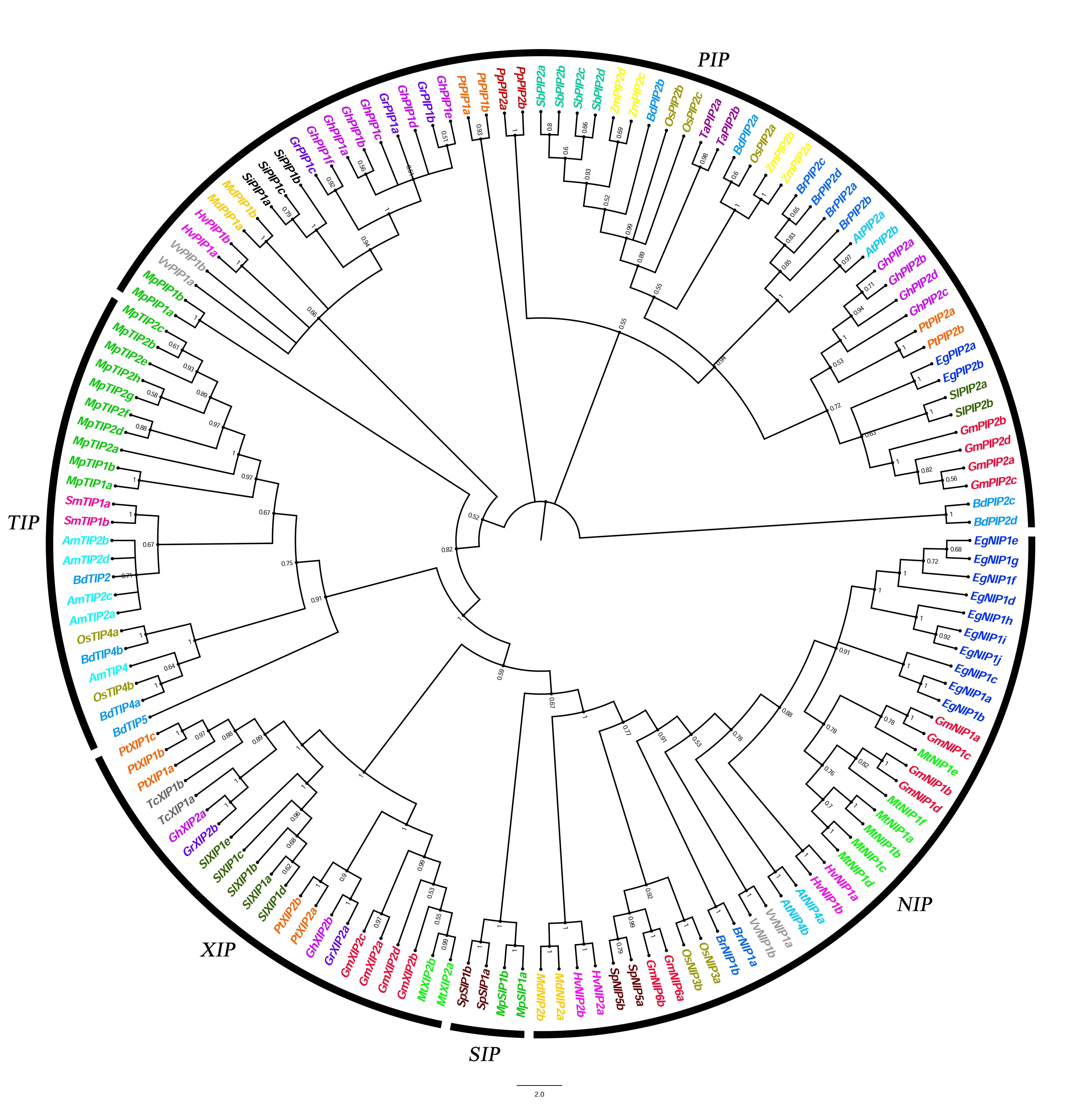

Supplement: Supplementary file 9 — Figure S3. Phylogenetic relationships of AQP tandem repeat genes from 24 well-studied species. The rooted NJ tree was constructed using MEGA 7, and the bootstrap test was performed with 1000 replicates. Am: A. trichopoda; At: A. thaliana; Bd: B. distachyon; Br: B. rapa; Eg: E. grandis; Gh: G. hirsutum; Gm: G. max; Gr: G. raimondii; Hv: H. vulgare; Md: M. domestica; Mp: M. polymorpha; Mt.: M. truncatula; Os: O. sativa; Pp: P. patens; Pt: P. trichocarpa; Sb: S. bicolor; Si: S. indicum; Sl: S. lycopersicum; Sm: S. moellendorffii; Sp: S. polyrhiza; Ta: T. aestivum; Tc: T. cacao; Vv: V. vinifera; Zm: Z. mays. (TIFF 9311 kb) [file 12864_2019_5928_MOESM9_ESM.tiff]

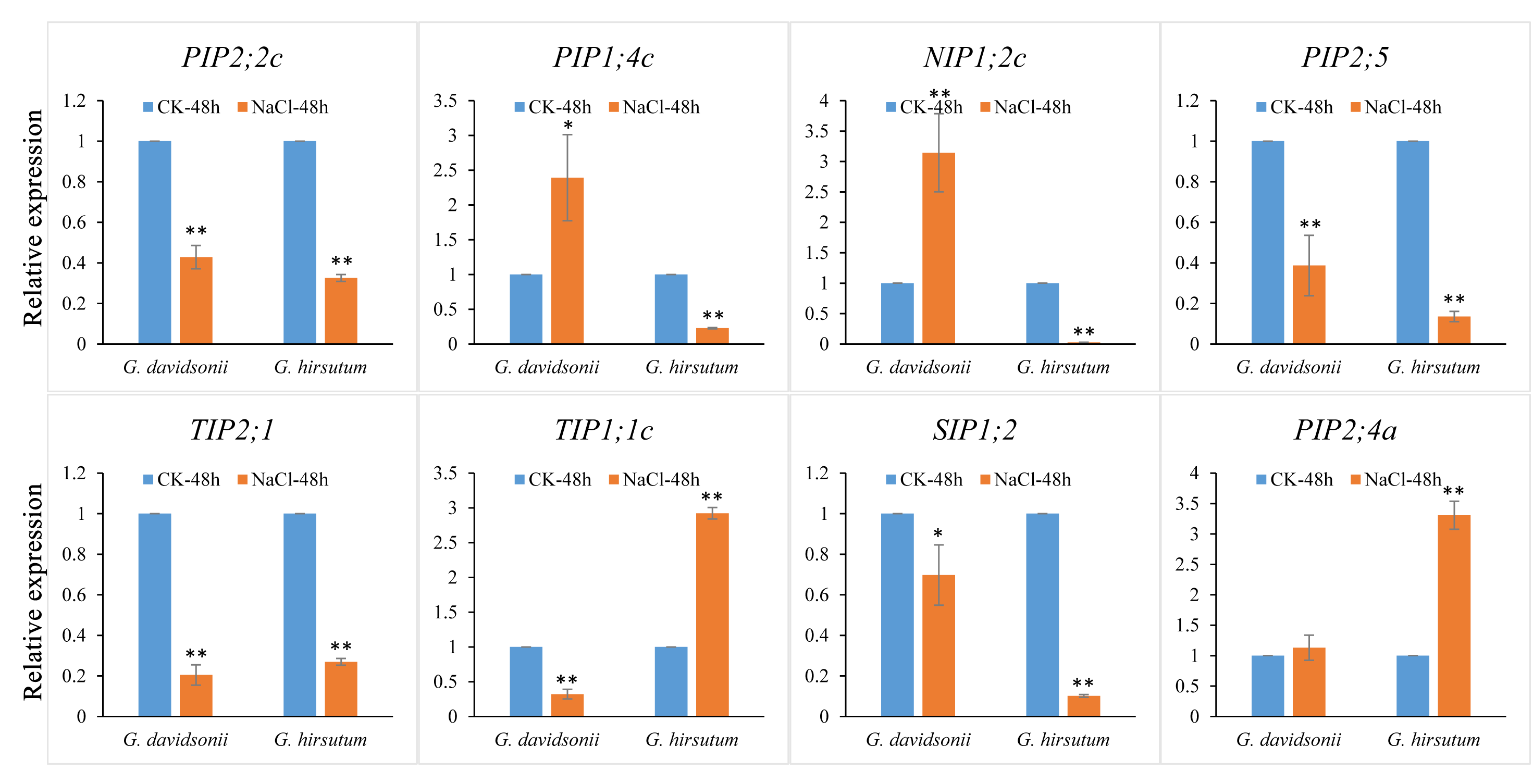

Supplement: Supplementary file 10 — Figure S4. Column charts of qRT-PCR results of the AQP genes in roots of G. davidsonii and G. hirsutum L. acc. TM-1 at 48 h post-treatment with salt stress. At the two true leaves and one heart shaped leaf stage, cotton seedlings were treated with 200 mM NaCl. The cotton Histone3 gene was used as an internal control. Values presented are means of three independent experiments, with error bars indicating standard deviations. (TIFF 448 kb) [file 12864_2019_5928_MOESM10_ESM.tiff]
