# Supplementary material for: Combining genome-wide and transcriptome-wide analyses reveal the evolutionary conservation and functional diversity of aquaporins in cotton
Source: BMC Genomics. 2019 Jul 1;20:538. doi: 10.1186/s12864-019-5928-2 (PMC6604486; doi:10.1186/s12864-019-5928-2)
Supplement: Supplementary file 3 — Table S2. Intra- or inter-genome duplications of AQP genes in G. raimondii, A. thaliana and O. sativa. (DOCX 18 kb) [file 12864_2019_5928_MOESM3_ESM.docx]

**Additional file 3: Table S2. Intra- or inter-genome duplications of AQP genes in *G. raimondii*, *A. thaliana* and *O. sativa*.**

| **Species-Species^*^** | **ID 1** | ***Gene 1*** | **ID 2** | ***Gene 2*** | **Ka** | **Ks** |
| --- | --- | --- | --- | --- | --- | --- |
| Os-Os | LOC_Os02g13870 | *OsNIP1;1* | LOC_Os06g35930 | *OsNIP1;4* | 0.2886 | 0.5426 |
| Os-Os | LOC_Os05g11560 | *OsNIP1;3* | LOC_Os06g35930 | *OsNIP1;4* | 0.3644 | 0.68 |
| Os-Os | LOC_Os01g10600 | *OsNIP1;2* | LOC_Os02g13870 | *OsNIP1;1* | 0.3537 | 0.8952 |
| Os-Os | LOC_Os02g51110 | *OsNIP2;1* | LOC_Os06g12310 | *OsNIP2;2* | 0.1525 | 0.3835 |
| Os-Os | LOC_Os02g44080 | *OsTIP2;1* | LOC_Os04g46490 | *OsTIP5;1* | 0.4808 | 0.7016 |
| Os-Os | LOC_Os04g44570 | *OsTIP3;2* | LOC_Os10g35050 | *OsTIP3;1* | 0.3365 | 0.58 |
| Os-Os | LOC_Os01g13120 | *OsTIP4;3* | LOC_Os05g14240 | *OsTIP4;1* | 0.2747 | 0.5722 |
| Os-Os | LOC_Os02g44630 | *OsPIP1;1* | LOC_Os04g47220 | *OsPIP1;2* | 0.0406 | 0.4147 |
| Os-Os | LOC_Os02g41860 | *OsPIP2;2* | LOC_Os10g34000 |  | 0.2614 | 0.5103 |
| Os-Os | LOC_Os04g44060 | *OsPIP2;3* | LOC_Os10g34000 |  | 0.2486 | 0.5078 |
| At-At | AT2G34390 | *AtNIP2;1* | AT4G19030 | *AtNIP1;1* | 0.3365 | 2.3243 |
| At-At | AT1G31885 | *AtNIP3;1* | AT4G19030 | *AtNIP1;1* | 0.3955 | 1.6182 |
| At-At | AT3G61430 | *AtPIP1;1* | AT4G00430 | *AtPIP1;4* | 0.0481 | 1.3132 |
| At-At | AT2G45960 | *AtPIP1;2* | AT4G00430 | *AtPIP1;4* | 0.1133 | 1.0475 |
| At-At | AT1G01620 | *AtPIP1;3* | AT4G00430 | *AtPIP1;4* | 0.0205 | 0.889 |
| At-At | AT2G37170 | *AtPIP2;2* | AT3G54820 | *AtPIP2;5* | 0.1589 | 2.07 |
| At-At | AT3G54820 | *AtPIP2;5* | AT5G60660 | *AtPIP2;4* | 0.1812 | 1.7707 |
| At-At | AT2G39010 | *AtPIP2;6* | AT3G54820 | *AtPIP2;5* | 0.1428 | 1.0269 |
| At-At | AT3G04090 | *AtSIP1;1* | AT5G18290 | *AtSIP1;2* | 0.1789 | 0.8332 |
| At-At | AT4G17340 | *AtTIP2;2* | AT5G47450 | *AtTIP2;3* | 0.0487 | 1.0119 |
| At-At | AT1G17810 | *AtTIP3;2* | AT1G73190 | *AtTIP3;1* | 0.0958 | 1.1538 |
| Gr-Gr | Gorai.010G162400 | *GrNIP1;2a* | Gorai.010G240400 | *GrNIP1;2b* | 0.1144 | 0.756 |
| Gr-Gr | Gorai.010G240400 | *GrNIP1;2b* | Gorai.011G035800 | *GrNIP1;2c* | 0.1201 | 0.7087 |
| Gr-Gr | Gorai.003G158100 | *GrPIP1;4a* | Gorai.004G212800 | *GrPIP1;4b* | 0.0163 | 0.3852 |
| Gr-Gr | Gorai.004G212800 | *GrPIP1;4b* | Gorai.007G100000 | *GrPIP1;4d* | 0.0624 | 0.9417 |
| Gr-Gr | Gorai.006G181300 | *GrPIP1;4c* | Gorai.013G019300 | *GrPIP1;4g* | 0.1133 | 2.0548 |
| Gr-Gr | Gorai.007G378400 | *GrPIP1;4e* | Gorai.013G019300 | *GrPIP1;4g* | 0.1198 | 1.5431 |
| Gr-Gr | Gorai.010G198800 | *GrPIP1;4f* | Gorai.011G283200 | *GrPIP1;4h* | 0.0256 | 0.5429 |
| Gr-Gr | Gorai.011G283200 | *GrPIP1;4h* | Gorai.013G019300 | *GrPIP1;4g* | 0.0575 | 0.5886 |
| Gr-Gr | Gorai.006G166800 | *GrPIP2;1* | Gorai.009G418100 | *GrPIP2;2c* | 0.0381 | 0.4034 |
| Gr-Gr | Gorai.002G248400 | *GrPIP2;2a* | Gorai.006G166800 | *GrPIP2;1* | 0.0351 | 0.4931 |
| Gr-Gr | Gorai.004G229000 | *GrPIP2;2b* | Gorai.009G107200 | *GrPIP2;4c* | 0.0563 | 0.5876 |
| Gr-Gr | Gorai.003G141600 | *GrPIP2;4a* | Gorai.004G229000 | *GrPIP2;2b* | 0.0564 | 0.6404 |
| Gr-Gr | Gorai.002G002500 | *GrPIP2;7a* | Gorai.002G198900 | *GrPIP2;7b* | 0.1288 | 1.1111 |
| Gr-Gr | Gorai.002G198900 | *GrPIP2;7b* | Gorai.005G254400 | *GrPIP2;8* | 0.1276 | 1.004 |
| Gr-Gr | Gorai.005G254400 | *GrPIP2;8* | Gorai.007G191600 | *GrPIP2;7c* | 0.1306 | 3.3014 |
| Gr-Gr | Gorai.011G214600 | *GrSIP1;1d* | Gorai.012G017700 | *GrSIP1;1b* | 0.2677 | 0.7479 |
| Gr-Gr | Gorai.007G268300 | *GrSIP1;2* | Gorai.013G191500 | *GrSIP1;1c* | 0.156 | 0.4263 |
| Gr-Gr | Gorai.002G245900 | *GrTIP1;1a* | Gorai.007G372700 | *GrTIP1;1b* | 0.059 | 0.7403 |
| Gr-Gr | Gorai.007G372700 | *GrTIP1;1b* | Gorai.009G413000 | *GrTIP1;1c* | 0.0505 | 0.8291 |
| Gr-Gr | Gorai.003G136600 | *GrTIP1;3a* | Gorai.011G253100 | *GrTIP1;3c* | 0.1148 | 1.327 |
| Gr-Gr | Gorai.009G101700 | *GrTIP1;3b* | Gorai.011G253100 | *GrTIP1;3c* | 0.1122 | 1.45 |
| Gr-Gr | Gorai.003G064000 | *GrTIP2;1* | Gorai.010G107900 | *GrTIP4;1* | 0.3562 | 1.7637 |
| Gr-Gr | Gorai.003G171800 | *GrTIP2;3a* | Gorai.004G197600 | *GrTIP2;3b* | 0.0526 | 0.6122 |
| Gr-Gr | Gorai.002G192500 | *GrXIP1;1* | Gorai.008G033000 | *GrXIP2;1* | 0.5437 | -1 |
| At-Gr | AT3G61430 | *AtPIP1;1* | Gorai.007G100000 | *GrPIP1;4d* | 0.0834 | 1.2062 |
| At-Gr | AT2G45960 | *AtPIP1;2* | Gorai.007G100000 | *GrPIP1;4d* | 0.1467 | 1.9336 |
| At-Gr | AT1G01620 | *AtPIP1;3* | Gorai.007G100000 | *GrPIP1;4d* | 0.0775 | 1.9436 |
| At-Gr | AT4G00430 | *AtPIP1;4* | Gorai.007G100000 | *GrPIP1;4d* | 0.0716 | 1.5914 |
| At-Gr | AT4G23400 | *AtPIP1;5* | Gorai.003G158100 | *GrPIP1;4a* | 0.0799 | 1.3285 |
| At-Gr | AT3G04090 | *AtSIP1;1* | Gorai.007G268300 | *GrSIP1;2* | 0.2833 | 1.2602 |
| At-Gr | AT5G18290 | *AtSIP1;2* | Gorai.013G191500 | *GrSIP1;1c* | 0.373 | 1.6348 |

****At, Os and Gr indicated A. thaliana, O. sativa and G. raimondii, respectively.***
