# Supplementary material for: Combining genome-wide and transcriptome-wide analyses reveal the evolutionary conservation and functional diversity of aquaporins in cotton
Source: BMC Genomics. 2019 Jul 1;20:538. doi: 10.1186/s12864-019-5928-2 (PMC6604486; doi:10.1186/s12864-019-5928-2)
Supplement: Supplementary file 5 — Table S3. Conserved motifs, selectivity filter and amino acid residues of AQPs in G. raimondii. (DOCX 20 kb) [file 12864_2019_5928_MOESM5_ESM.docx]

**Additional file 5: Table S3. Conserved motifs, selectivity filter and amino acid residues of AQPs in *G. raimondii*.**

| **Loci** | **NPA (LB)** | **NPA (LE)** | **ar/R selectivity filters** | | | | **Froger’s Residue** | | | | |
| --- | --- | --- | --- | --- | --- | --- | --- | --- | --- | --- | --- |
|  |  |  | **H2** | **H5** | **LE1** | **LE2** | **P1** | **P2** | **P3** | **P4** | **P5** |
| **Plasma membrane intrinsic proteins (PIPs)** | | | | | | | | | | | |
| GrPIP2;4a | NPA | NPA | F | H | T | R | Q | S | A | F | W |
| GrPIP2;4c | NPA | NPA | F | H | T | R | Q | S | A | F | W |
| GrPIP2;2d | NPA | NPA | F | H | T | R | Q | S | A | F | W |
| GrPIP2;2b | NPA | NPA | F | H | T | R | Q | S | A | F | W |
| GrPIP2;4b | — | NLA | — | H | T | R | Q | S | A | — | — |
| GrPIP2;4d | EPG | NPA | — | H | T | R | Q | S | A | — | — |
| GrPIP2;5a | NPA | NPA | F | H | T | R | Q | S | A | F | W |
| GrPIP2;5b | NPA | NPA | F | H | T | R | Q | S | A | F | W |
| GrPIP2;2c | NPA | NPA | F | H | T | R | Q | S | A | F | W |
| GrPIP2;1 | NPA | NPA | F | H | T | R | Q | S | A | F | W |
| GrPIP2;2a | NPA | NPA | F | H | T | R | Q | S | A | F | W |
| GrPIP2;7b | NPA | NPA | F | H | T | R | M | S | A | II | F |
| GrPIP2;7a | NPA | NPA | F | H | T | R | M | S | A | F | W |
| GrPIP2;8 | NPA | NPA | F | H | T | R | M | S | A | F | W |
| GrPIP2;7c | NPA | NPA | V | H | T | R | M | S | A | F | W |
| GrPIP2;7d | NPA | NPA | V | H | T | R | M | S | A | F | W |
| GrPIP1;4c | NPA | NPA | F | H | T | R | Q | S | A | F | W |
| GrPIP1;4e | NPA | NPA | F | H | T | R | Q | S | A | F | W |
| GrPIP1;4a | NPA | NPA | F | H | T | R | E | S | A | F | W |
| GrPIP1;4b | NPA | NPA | F | H | T | R | Q | S | A | F | W |
| GrPIP1;4d | NPA | NPA | F | H | T | R | Q | S | A | F | W |
| GrPIP1;4g | NPA | NPA | F | H | T | R | M | S | A | F | W |
| GrPIP1;4f | NPA | NPA | F | H | T | R | M | S | A | F | W |
| GrPIP1;4j | NPA | NPA | F | H | T | R | M | S | A | F | W |
| GrPIP1;4h | NPA | NPA | F | H | T | R | M | S | A | F | W |
| GrPIP1;4i | NPA | NPA | F | H | T | R | M | S | A | F | W |
| **Tonoplast intrinsic proteins (TIPs)** | | | | | | | | | | | |
| GrTIP2;3a | NPA | NPA | H | I | G | R | T | S | A | Y | W |
| GrTIP2;3b | NPA | NPA | H | I | G | R | T | S | A | Y | W |
| GrTIP2;1 | NPA | NPA | H | I | G | R | T | S | A | Y | W |
| GrTIP5;1 | NPA | NPA | N | V | G | L | T | A | A | Y | W |
| GrTIP4;1 | NPA | NPA | H | I | A | R | T | S | A | Y | W |
| GrTIP3;2 | NPA | NPA | H | I | A | R | A | S | A | Y | W |
| GrTIP1;1a | NPA | NPA | H | I | A | V | T | A | A | Y | W |
| GrTIP1;1b | NPA | NPA | H | I | A | V | T | S | A | Y | W |
| GrTIP1;1c | NPA | NPA | H | I | A | V | T | S | A | Y | W |
| GrTIP1;3c | NPA | NPA | H | I | A | V | S | S | A | Y | W |
| GrTIP1;3d | NPA | NPA | H | I | A | V | T | S | A | Y | W |
| GrTIP1;3a | NPA | NPA | H | I | A | V | T | S | A | Y | W |
| GrTIP1;3b | NPA | NPA | H | I | A | V | T | S | A | Y | W |
| **Nodulin-26 like intrinsic proteins (NIPs)** | | | | | | | | | | | |
| GrNIP7;1a | NPA | NPA | A | V | G | R | Y | S | A | Y | L |
| GrNIP7;1b | NPA | NPA | A | V | G | R | Y | S | A | Y | L |
| GrNIP1;1 | NPA | NPA | W | V | A | R | F | S | A | Y | I |
| GrNIP4;2 | NPA | NPA | G | S | G | R | L | S | A | Y | V |
| GrNIP5;1 | NPS | NPV | A | I | G | R | F | T | A | Y | L |
| GrNIP6;1 | NPA | NPV | T | I | A | R | F | T | A | Y | F |
| GrNIP4;1 | NPA | NPA | W | V | A | R | W | T | A | Y | M |
| GrNIP1;2b | NPA | NPA | W | V | A | R | F | S | A | Y | V |
| GrNIP1;2a | NPA | NPA | W | V | A | R | F | S | A | Y | L |
| GrNIP1;2c | NPA | NPA | W | V | A | R | F | S | A | Y | L |
| **Small basic intrinsic proteins (SIPs)** | | | | | | | | | | | |
| GrSIP2;1 | NPL | NPA | V | H | G | S | F | V | A | Y | W |
| GrSIP1;1c | DPA | NPA | F | V | P | F | I | A | A | Y | W |
| GrSIP1;2 | NPT | NPA | I | V | P | N | M | A | A | Y | W |
| GrSIP1;1d | NPA | NPA | V | V | A | S | I | A | A | Y | W |
| GrSIP1;1a | NPT | NPA | V | V | P | N | L | A | A | Y | W |
| GrSIP1;1b | NPT | NPA | V | V | P | N | L | A | A | Y | W |
| **Uncharacterized X intrinsic proteins (XIPs)** | | | | | | | | | | | |
| GrXIP2;1 | SPV | NPA | V | I | V | R | M | C | A | F | W |
| GrXIP2;2 | NPV | NPA | I | T | V | R | V | C | A | F | W |
| GrXIP1;1 | NPI | NPA | I | T | A | R | V | C | A | F | W |
