# Supplementary material for: Combining genome-wide and transcriptome-wide analyses reveal the evolutionary conservation and functional diversity of aquaporins in cotton
Source: BMC Genomics. 2019 Jul 1;20:538. doi: 10.1186/s12864-019-5928-2 (PMC6604486; doi:10.1186/s12864-019-5928-2)
Supplement: Supplementary file 6 — Table S4. Amount of AQP isoforms in 30 species from algae to angiosperm based on E-value < e− 10 and query coverage > 50%. (DOCX 18 kb) [file 12864_2019_5928_MOESM6_ESM.docx]

**Additional file 6: Table S4. Amount of AQP isoforms in 30 species from algae to angiosperm based on E-value < e^-10^ and query coverage > 50%.**

| **Species** | **Number of isoforms in subfamilies** | | | | | | | **Total isoforms** | **Total subfamilies** |
| --- | --- | --- | --- | --- | --- | --- | --- | --- | --- |
|  | **PIPs** | **NIPs** | **TIPs** | **SIPs** | **XIPs** | **HIPs** | **GIPs** |  |  |
| *Arabidopsis thaliana* | **13** (37.1)^*^ | **9** (25.7) | **10** (28.6) | **3** (8.6) | - | - | - | **35** | **4** |
| *Brassica rapa* | **22** (37.3) | **15** (25.4) | **16** (27.1) | **6** (10.2) | - | - | - | **59** | **4** |
| *Glycine max* | **26** (29.5) | **24** (27.3) | **26** (29.5) | **8** (9.1) | **4** (4.5) | - | - | **88** | **5** |
| *Medicago truncatula* | **10** (21.7) | **16** (34.8) | **14** (30.4) | **4** (8.7) | **2** (4.3) | - | - | **46** | **5** |
| *Malus domestica* | **15** (20.3) | **26** (35.1) | **22** (29.7) | **8** (10.8) | **3** (4.1) | - | - | **74** | **5** |
| *Theobroma cacao* | **10** (32.3) | **7** (22.6) | **8** (25.8) | **3** (9.7) | **3** (9.7) | - | - | **31** | **5** |
| *Gossypium arboreum* | **23** (42.6) | **9** (16.7) | **13** (24.1) | **6** (11.1) | **3** (5.6) | - | - | **54** | **5** |
| *Gossypium raimondii* | **26** (44.8) | **10** (17.2) | **13** (22.4) | **6** (10.3) | **3** (5.2) | - | - | **58** | **5** |
| *Gossypium hirsutum* | **50** (44.2) | **20** (17.7) | **26** (23) | **11** (9.7) | **6** (5.3) | - | - | **113** | **5** |
| *Gossypium barbadense* | **54** (48.2) | **18** (16.1) | **24** (21.4) | **10** (8.9) | **6** (5.4) | - | - | **112** | **5** |
| *Sesamum indicum* | **13** (36.1) | **8** (22.2) | **12** (33.3) | **2** (5.6) | **1** (2.8) | - | - | **36** | **5** |
| *Populus trichocarpa* | **15** (27.3) | **11** (20) | **17** (30.9) | **6** (10.9) | **6** (10.9) | - | - | **55** | **5** |
| *Vitis vinifera* | **10** (30.3) | **9** (27.3) | **10** (30.3) | **2** (6.1) | **2** (6.1) | - | - | **33** | **5** |
| *Eucalyptus grandis* | **14** (28.6) | **19** (38.8) | **14** (28.6) | **1** (2) | **1** (2) | - | - | **49** | **5** |
| *Solanum lycopersicum* | **14** (29.8) | **12** (25.5) | **11** (23.4) | **4** (8.5) | **6** (12.8) | - | - | **47** | **5** |
| *Zea mays* | **13** (41.9) | **4** (12.9) | **11** (35.5) | **3** (9.7) | **-** | - | - | **31** | **4** |
| *Sorghum bicolor* | **13** (34.2) | **11** (28.9) | **11** (28.9) | **3** (7.9) | **-** | - | - | **38** | **4** |
| *Triticum aestivum* | **57** (46.3) | **30** (24.4) | **31** (25.2) | **5** (4.1) | **-** | - | - | **123** | **4** |
| *Hordeum vulgare* | **19** (47.5) | **8** (20) | **11** (27.5) | **2** (5) | **-** | - | - | **40** | **4** |
| *Oryza sativa* | **11** (33.3) | **10** (30.3) | **10** (30.3) | **2** (6.1) | **-** | - | - | **33** | **4** |
| *Brachypodium distachyon* | **10** (35.7) | **7** (25) | **10** (35.7) | **1** (3.6) | **-** | - | - | **28** | **4** |
| *Amborella trichopoda* | **5** (22.7) | **7** (31.8) | **7** (31.8) | **3** (13.6) | **-** | - | - | **22** | **4** |
| *Selaginella moellendorffii* | **3** (15.8) | **8** (42.1) | **2** (10.5) | **1** (5.3) | **3** (15.8) | **2** (10.5) | - | **19** | **6** |
| *Physcomitrella patens* | **8** (34.8) | **5** (21.7) | **4** (17.4) | **2** (8.7) | **2** (8.7) | **1** (4.3) | **1** (4.3) | **23** | **7** |
| *Marchantia polymorpha* | **19** (51.4) | **1** (2.7) | **11** (29.7) | **3** (8.1) | **1** (2.7) | **2** (5.4) | - | **37** | **6** |
| *Klebsormidium flaccidum* | **1** (25) | **1** (25) | - | **1** (25) | - | **1** (25) | - | **4** | **4** |
| *Chlamydomonas reinhardtii* | - | - | - | **1** (33.3) | - | **2** (66.7) | - | **3** | **2** |
| *Volvox carteri* | - | - | - | **1** (33.3) | - | **2** (66.7) | - | **3** | **2** |
| *Ostreococcus lucimarinus* | - | - | - | - | - | **1** (100) | - | **1** | **1** |
| *Cyanidioschyzon merolae* | - | - | - | - | - | **1** (100) | - | **1** | **1** |

**** Relative size of subfamily in the species (%).***
