# Supplementary material for: Combining genome-wide and transcriptome-wide analyses reveal the evolutionary conservation and functional diversity of aquaporins in cotton
Source: BMC Genomics. 2019 Jul 1;20:538. doi: 10.1186/s12864-019-5928-2 (PMC6604486; doi:10.1186/s12864-019-5928-2)
Supplement: Supplementary file 7 — Table S5. Similarity analysis for the evolution of AQP isoforms in 30 species from algae to angiosperm based on E-value < e− 10 and query coverage > 50%. (DOCX 17 kb) [file 12864_2019_5928_MOESM7_ESM.docx]

**Additional file 7: Table S5. Similarity analysis for the evolution of AQP isoforms in 30 species from algae to angiosperm based on E-value < e^-10^ and query coverage > 50%.**

| **Species** | **Similarity of isoforms in subfamilies** | | | | | | |
| --- | --- | --- | --- | --- | --- | --- | --- |
|  | **PIPs** | **NIPs** | **TIPs** | **SIPs** | **XIPs** | **HIPs** | **GIPs** |
| *Arabidopsis thaliana* | 76.32^*^ | 51.01 | 50.53 | 39.88 | - | - | - |
| *Brassica rapa* | 74.37 | 53.12 | 50.58 | 39.02 | - | - | - |
| *Glycine max* | 73.51 | 51.82 | 48.49 | 43.24 | 34.12 | - | - |
| *Medicago truncatula* | 74.85 | 54.05 | 46.31 | 41.04 | 31.64 | - | - |
| *Malus domestica* | 64.71 | 52.83 | 45.67 | 40.77 | 34.01 | - | - |
| *Theobroma cacao* | 74.75 | 51.46 | 50.04 | 42.11 | 34.10 | - | - |
| *Gossypium arboreum* | 74.76 | 50.99 | 50.79 | 43.57 | 35.10 | - | - |
| *Gossypium raimondii* | 75.16 | 51.47 | 50.79 | 42.66 | 35.03 | - | - |
| *Gossypium hirsutum* | 75.28 | 51.74 | 50.48 | 42.60 | 34.43 | - | - |
| *Gossypium barbadense* | 74.90 | 49.21 | 49.75 | 42.39 | 34.76 | - | - |
| *Sesamum indicum* | 75.26 | 50.88 | 48.27 | 51.03 | 35.71 | - | - |
| *Populus trichocarpa* | 74.12 | 55.71 | 50.35 | 42.02 | 35.87 | - | - |
| *Vitis vinifera* | 79.06 | 50.48 | 47.26 | 45.78 | 32.40 | - | - |
| *Eucalyptus grandis* | 74.03 | 51.38 | 48.35 | 52.79 | 36.78 | - | - |
| *Solanum lycopersicum* | 75.04 | 48.94 | 50.01 | 41.49 | 34.41 | - | - |
| *Zea mays* | 75.60 | 53.98 | 48.20 | 46.18 | - | - | - |
| *Sorghum bicolor* | 74.65 | 50.02 | 47.95 | 45.65 | - | - | - |
| *Triticum aestivum* | 73.54 | 52.19 | 48.38 | 40.87 | - | - | - |
| *Hordeum vulgare* | 74.05 | 50.01 | 48.02 | 50.22 | - | - | - |
| *Oryza sativa* | 73.73 | 49.44 | 46.97 | 41.76 | - | - | - |
| *Brachypodium distachyon* | 72.57 | 51.31 | 47.10 | 49.58 | - | - | - |
| *Amborella trichopoda* | 76.03 | 54.55 | 53.58 | 41.60 | - | - | - |
| *Selaginella moellendorffii* | 74.66 | 55.77 | 57.68 | 50.63 | 41.04 | 59.88 | - |
| *Physcomitrella patens* | 100.00 | 100.00 | 100.00 | 100.00 | 100.00 | 100.00 | 100.00 |
| *Marchantia polymorpha* | 65.10 | 68.98 | 61.29 | 47.26 | 34.14 | 58.27 | - |
| *Klebsormidium flaccidum* | 49.57 | 51.18 | - | 37.29 | - | 42.82 | - |
| *Chlamydomonas reinhardtii* | - | - | - | 27.31 | - | 35.99 | - |
| *Volvox carteri* | - | - | - | 26.20 | - | 34.06 | - |
| *Ostreococcus lucimarinus* | - | - | - | - | - | 26.09 | - |
| *Cyanidioschyzon merolae* | - | - | - | - | - | 31.14 | - |

**** The average similarity of subfamily in the species (%).***
