# Supplementary material for: Combining genome-wide and transcriptome-wide analyses reveal the evolutionary conservation and functional diversity of aquaporins in cotton
Source: BMC Genomics. 2019 Jul 1;20:538. doi: 10.1186/s12864-019-5928-2 (PMC6604486; doi:10.1186/s12864-019-5928-2)
Supplement: Supplementary file 8 — Table S6. Overview of AQP tandem repeat genes in green plants. (DOCX 26 kb) [file 12864_2019_5928_MOESM8_ESM.docx]

**Additional file 8: Table S6. Overview of AQP tandem repeat genes in green plants.**

| ***Taxa name*** | **Organism** | **Phytozome ID** | **Location** | **Start** | **End** |
| --- | --- | --- | --- | --- | --- |
| *AmTIP2a* | *Amborella trichopoda* | evm_27.model.AmTr_v1.0  _scaffold00106.125 | scaffold_00106 | 2258434 | 2260052 |
| *AmTIP2b* |  | evm_27.model.AmTr_v1.0_  scaffold00106.126 | scaffold_00106 | 2271995 | 2272383 |
| *AmTIP2c* |  | evm_27.model.AmTr_v1.0_  scaffold00106.127 | scaffold_00106 | 2273282 | 2273602 |
| *AmTIP2d* |  | evm_27.model.AmTr_v1.0_  scaffold00109.157 | scaffold_00109 | 2124432 | 2126997 |
| *AmTIP4* |  | evm_27.model.AmTr_v1.0_  scaffold00109.156 | scaffold_00109 | 2107136 | 2110068 |
| *AtNIP4a* | *Arabidopsis thaliana* | AT5G37810.1 | Chr05 | 15045232 | 15047807 |
| *AtNIP4b* |  | AT5G37820.1 | Chr05 | 15050261 | 15051542 |
| *AtPIP2a* |  | AT2G37170.1 | Chr02 | 15613402 | 15614857 |
| *AtPIP2b* |  | AT2G37180.1 | Chr02 | 15617708 | 15619155 |
| *BdPIP2a* | *Brachypodium distachyon* | Bradi1g28760.1 | Chr01 | 24115585 | 24118617 |
| *BdPIP2b* |  | Bradi1g28780.1 | Chr01 | 24143877 | 24145345 |
| *BdPIP2c* |  | Bradi4g36601.1 | Chr04 | 41709704 | 41711325 |
| *BdPIP2d* |  | Bradi4g36610.1 | Chr04 | 41713192 | 41714682 |
| *BdTIP2* |  | Bradi5g17690.1 | Chr05 | 21052804 | 21053722 |
| *BdTIP4a* |  | Bradi2g07810.1 | Chr02 | 6166394 | 6167158 |
| *BdTIP4b* |  | Bradi2g07830.1 | Chr02 | 6185272 | 6187133 |
| *BdTIP5* |  | Bradi5g17680.1 | Chr05 | 21051238 | 21052610 |
| *BrNIP1a* | *Brassica rapa* | Brara.E00984.1 | Chr05 | 5738734 | 5740318 |
| *BrNIP1b* |  | Brara.E00986.1 | Chr05 | 5746585 | 5747547 |
| *BrPIP2a* |  | Brara.C01861.1 | Chr03 | 9183943 | 9185318 |
| *BrPIP2b* |  | Brara.C01862.1 | Chr03 | 9188805 | 9190225 |
| *BrPIP2c* |  | Brara.E00787.1 | Chr05 | 4556433 | 4557926 |
| *BrPIP2d* |  | Brara.E00788.1 | Chr05 | 4561021 | 4562385 |
| *EgNIP1a* | *Eucalyptus grandis* | Eucgr.D00421.1 | Chr04 | 7907741 | 7909857 |
| *EgNIP1b* |  | Eucgr.D00422.1 | Chr04 | 7970775 | 7973178 |
| *EgNIP1c* |  | Eucgr.D00423.1 | Chr04 | 7996850 | 7999822 |
| *EgNIP1d* |  | Eucgr.E02324.1 | Chr05 | 35924878 | 35927744 |
| *EgNIP1e* |  | Eucgr.E02325.1 | Chr05 | 35899998 | 35903001 |
| *EgNIP1f* |  | Eucgr.E02326.1 | Chr05 | 35887332 | 35890323 |
| *EgNIP1g* |  | Eucgr.E02327.1 | Chr05 | 35864700 | 35867725 |
| *EgNIP1h* |  | Eucgr.E02836.1 | Chr05 | 47182593 | 47185382 |
| *EgNIP1i* |  | Eucgr.E02837.1 | Chr05 | 47164956 | 47165900 |
| *EgNIP1j* |  | Eucgr.E02838.1 | Chr05 | 47148225 | 47149417 |
| *EgPIP2a* |  | Eucgr.B02499.1 | Chr02 | 45012546 | 45014038 |
| *EgPIP2b* |  | Eucgr.B02500.1 | Chr02 | 45017345 | 45018891 |
| *GhPIP1a* | *Gossypium hirsutum* | Gohir.A10G233200.1 | Chr10 | 110716149 | 110717666 |
| *GhPIP1b* |  | Gohir.A10G233300.1 | Chr10 | 110724152 | 110725599 |
| *GhPIP1c* |  | Gohir.A10G233400.1 | Chr10 | 110734899 | 110736309 |
| *GhPIP1d* |  | Gohir.D10G245500.1 | Chr23 | 66873650 | 66875288 |
| *GhPIP1e* |  | Gohir.D10G245600.1 | Chr23 | 66877736 | 66879141 |
| *GhPIP1f* |  | Gohir.D10G245700.1 | Chr23 | 66884503 | 66885942 |
| *GhPIP2a* |  | Gohir.A01G215200.1 | Chr01 | 102352149 | 102353643 |
| *GhPIP2b* |  | Gohir.A01G215300.1 | Chr01 | 102357017 | 102358511 |
| *GhPIP2c* |  | Gohir.D01G203800.1 | Chr13 | 62763931 | 62765117 |
| *GhPIP2d* |  | Gohir.D01G203900.1 | Chr13 | 62772529 | 62773659 |
| *GhXIP2a* |  | Gohir.A12G039900.1 | Chr12 | 7450708 | 7452049 |
| *GhXIP2b* |  | Gohir.A12G040000.1 | Chr12 | 7462223 | 7463302 |
| *GmNIP1a* | *Glycine max* | Glyma.05G162500.1 | Chr05 | 35371190 | 35375992 |
| *GmNIP1b* |  | Glyma.05G162600.1 | Chr05 | 35376373 | 35378991 |
| *GmNIP1c* |  | Glyma.08G120100.1 | Chr08 | 9255018 | 9258625 |
| *GmNIP1d* |  | Glyma.08G120200.1 | Chr08 | 9260965 | 9263699 |
| *GmNIP6a* |  | Glyma.07G024400.1 | Chr07 | 1891263 | 1894097 |
| *GmNIP6b* |  | Glyma.07G024700.1 | Chr07 | 1928773 | 1931659 |
| *GmPIP2a* |  | Glyma.02G073600.1 | Chr02 | 6421649 | 6424849 |
| *GmPIP2b* |  | Glyma.02G073700.1 | Chr02 | 6434383 | 6437873 |
| *GmPIP2c* |  | Glyma.16G155000.1 | Chr16 | 31513389 | 31517035 |
| *GmPIP2d* |  | Glyma.16G155100.1 | Chr16 | 31522994 | 31524889 |
| *GmXIP2a* |  | Glyma.11G097700.1 | Chr11 | 7446833 | 7448518 |
| *GmXIP2b* |  | Glyma.11G097800.1 | Chr11 | 7449914 | 7450765 |
| *GmXIP2c* |  | Glyma.12G023600.1 | Chr12 | 1733920 | 1735494 |
| *GmXIP2d* |  | Glyma.12G023700.1 | Chr12 | 1736339 | 1736998 |
| *GrPIP1a* | *Gossypium raimondii* | Gorai.011G283200.1 | Chr11 | 61584423 | 61586095 |
| *GrPIP1b* |  | Gorai.011G283300.1 | Chr11 | 61588606 | 61590085 |
| *GrPIP1c* |  | Gorai.011G283400.1 | Chr11 | 61597649 | 61599166 |
| *GrXIP2a* |  | Gorai.008G033000.1 | Chr08 | 3972376 | 3973212 |
| *GrXIP2b* |  | Gorai.008G033100.1 | Chr08 | 3995102 | 3996763 |
| *HvNIP1a* | *Hordeum vulgare* | HORVU7Hr1G043590.2 | Chr07 | 133179343 | 133182208 |
| *HvNIP1b* |  | HORVU7Hr1G043600.1 | Chr07 | 133182157 | 133218711 |
| *HvNIP2a* |  | HORVU7Hr1G038220.1 | Chr07 | 95164287 | 95168362 |
| *HvNIP2b* |  | HORVU7Hr1G038270.1 | Chr07 | 95635702 | 95639466 |
| *HvPIP1a* |  | HORVU6Hr1G092960.5 | Chr06 | 578690600 | 578692405 |
| *HvPIP1b* |  | HORVU6Hr1G092970.3 | Chr06 | 578621093 | 578622182 |
| *MdNIP2a* | *Malus domestica* | MDP0000147323 | Chr17 | 3541874 | 3545200 |
| *MdNIP2b* |  | MDP0000147325 | Chr17 | 3551162 | 3554359 |
| *MdPIP1a* |  | MDP0000120792 | MDC003164.335 | 232 | 582 |
| *MdPIP1b* |  | MDP0000120794 | MDC003164.555 | 232 | 582 |
| *MpPIP1a* | *Marchantia polymorpha* | Mapoly0044s0027.1 | scaffold_44 | 325635 | 328491 |
| *MpPIP1b* |  | Mapoly0044s0029.1 | scaffold_44 | 367021 | 369773 |
| *MpSIP1a* |  | Mapoly0059s0002.1 | scaffold_59 | 13735 | 17899 |
| *MpSIP1b* |  | Mapoly0059s0003.1 | scaffold_59 | 14795 | 16433 |
| *MpTIP1a* |  | Mapoly0005s0187.1 | scaffold_5 | 1986661 | 1988382 |
| *MpTIP1b* |  | Mapoly0005s0188.1 | scaffold_5 | 1994467 | 1996038 |
| *MpTIP2a* |  | Mapoly0135s0053.1 | scaffold_135 | 446912 | 448954 |
| *MpTIP2b* |  | Mapoly0135s0054.1 | scaffold_135 | 452760 | 454675 |
| *MpTIP2c* |  | Mapoly0135s0055.1 | scaffold_135 | 456525 | 458630 |
| *MpTIP2d* |  | Mapoly0135s0056.1 | scaffold_135 | 462757 | 464651 |
| *MpTIP2e* |  | Mapoly0223s0004.1 | scaffold_223 | 45196 | 46799 |
| *MpTIP2f* |  | Mapoly0223s0005.1 | scaffold_223 | 50362 | 52231 |
| *MpTIP2g* |  | Mapoly0223s0006.1 | scaffold_223 | 55773 | 57104 |
| *MpTIP2h* |  | Mapoly0223s0007.1 | scaffold_223 | 63925 | 65783 |
| *MtNIP1a* | *Medicago truncatula* | Medtr2g017570.1 | Chr02 | 5388011 | 5391282 |
| *MtNIP1b* |  | Medtr2g017610.1 | Chr02 | 5401931 | 5404382 |
| *MtNIP1c* |  | Medtr2g017590.1 | Chr02 | 5396335 | 5398792 |
| *MtNIP1d* |  | Medtr2g017620.1 | Chr02 | 5406235 | 5409004 |
| *MtNIP1e* |  | Medtr8g087710.1 | Chr08 | 36261986 | 36264406 |
| *MtNIP1f* |  | Medtr8g087720.1 | Chr08 | 36267093 | 36269691 |
| *MtXIP2a* |  | Medtr4g075570.1 | Chr04 | 28881013 | 28882292 |
| *MtXIP2b* |  | Medtr4g075590.1 | Chr04 | 28885867 | 28887300 |
| *OsNIP3a* | *Oryza sativa* | LOC_Os08g05590.1 | Chr08 | 2989266 | 2991016 |
| *OsNIP3b* |  | LOC_Os08g05600.1 | Chr08 | 2997031 | 2998457 |
| *OsPIP2a* |  | LOC_Os07g26690.1 | Chr07 | 15406442 | 15409801 |
| *OsPIP2b* |  | LOC_Os07g26630.1 | Chr07 | 15358960 | 15360495 |
| *OsPIP2c* |  | LOC_Os07g26660.1 | Chr07 | 15376916 | 15378292 |
| *OsTIP4a* |  | LOC_Os01g13130.1 | Chr01 | 7302368 | 7303788 |
| *OsTIP4b* |  | LOC_Os01g13120.1 | Chr01 | 7297672 | 7298971 |
| *PpPIP2a* | *Physcomitrella patens* | Pp3c12_22530V3.1 | Chr12 | 14933191 | 14936021 |
| *PpPIP2b* |  | Pp3c12_22590V3.1 | Chr12 | 14962777 | 14965608 |
| *PtPIP1a* | *Populus trichocarpa* | Potri.005G109200.1 | Chr05 | 8388392 | 8390181 |
| *PtPIP1b* |  | Potri.005G109300.1 | Chr05 | 8392291 | 8395636 |
| *PtPIP2a* |  | Potri.006G128000.1 | Chr06 | 10443014 | 10444501 |
| *PtPIP2b* |  | Potri.006G128200.1 | Chr06 | 10448694 | 10450596 |
| *PtXIP1a* |  | Potri.009G127900.1 | Chr09 | 10510764 | 10511944 |
| *PtXIP1b* |  | Potri.009G128000.1 | Chr09 | 10518958 | 10520221 |
| *PtXIP1c* |  | Potri.009G128300.1 | Chr09 | 10526177 | 10527610 |
| *PtXIP2a* |  | Potri.009G128100.1 | Chr09 | 10521412 | 10522134 |
| *PtXIP2b* |  | Potri.009G128400.1 | Chr09 | 10528057 | 10528795 |
| *SbPIP2a* | *Sorghum bicolor* | Sobic.002G125000.1 | Chr02 | 16883369 | 16884816 |
| *SbPIP2b* |  | Sobic.002G125200.1 | Chr02 | 16897836 | 16899264 |
| *SbPIP2c* |  | Sobic.002G125300.1 | Chr02 | 16906986 | 16908387 |
| *SbPIP2d* |  | Sobic.002G125700.2 | Chr02 | 16980280 | 16982926 |
| *SiPIP1a* | *Sesamum indicum* | SIN_1012172^*^ | Chr04 | 13061085 | 13062763 |
| *SiPIP1b* |  | SIN_1012173^*^ | Chr04 | 13068798 | 13070724 |
| *SiPIP1c* |  | SIN_1012174^*^ | Chr04 | 13076533 | 13078784 |
| *SlPIP2a* | *Solanum lycopersicum* | Solyc09g007760.2.1 | Chr09 | 1295368 | 1298246 |
| *SlPIP2b* |  | Solyc09g007770.2.1 | Chr09 | 1300967 | 1302708 |
| *SlXIP1a* |  | Solyc10g054840.1.1 | Chr10 | 55996851 | 55998029 |
| *SlXIP1b* |  | Solyc10g054820.1.1 | Chr10 | 55972910 | 55974198 |
| *SlXIP1c* |  | Solyc10g054810.1.1 | Chr10 | 55955820 | 55957374 |
| *SlXIP1d* |  | Solyc10g054800.1.1 | Chr10 | 55952072 | 55953225 |
| *SlXIP1e* |  | Solyc10g054790.1.1 | Chr10 | 55950041 | 55951202 |
| *SmTIP1a* | *Selaginella moellendorffii* | 165030 | scaffold_1 | 4466973 | 4468294 |
| *SmTIP1b* |  | 165035 | scaffold_1 | 4468466 | 4469607 |
| *SpNIP5a* | *Spirodela polyrhiza* | Spipo5G0048900 | Chr05 | 4491897 | 4494335 |
| *SpNIP5b* |  | Spipo5G0049000 | Chr05 | 4505108 | 4508274 |
| *SpSIP1a* |  | Spipo29G0007000 | Chr29 | 492389 | 494348 |
| *SpSIP1b* |  | Spipo29G0007100 | Chr29 | 497295 | 499172 |
| *TaPIP2a* | *Triticum aestivum* | TraesCS2D01G376000.1 | 2D | 480179550 | 480181671 |
| *TaPIP2b* |  | TraesCS2D01G376100.1 | 2D | 480323643 | 480325533 |
| *TcXIP1a* | *Theobroma cacao* | Thecc1EG006705t1 | Chr02 | 3405256 | 3406887 |
| *TcXIP1b* |  | Thecc1EG006706t1 | Chr02 | 3407031 | 3409434 |
| *VvNIP1a* | *Vitis vinifera* | GSVIVT01004850001 | Chr07 | 49937 | 53027 |
| *VvNIP1b* |  | GSVIVT01004855001 | Chr07 | 110283 | 113460 |
| *VvPIP1a* |  | GSVIVT01026942001 | Chr15 | 19199215 | 19206926 |
| *VvPIP1b* |  | GSVIVT01026944001 | Chr15 | 19192581 | 19196103 |
| *ZmPIP2a* | *Zea mays PH207* | Zm00008a008655_T01 | Chr02 | 174430480 | 174433404 |
| *ZmPIP2b* |  | Zm00008a027384_T01 | Chr07 | 39320673 | 39325254 |
| *ZmPIP2c* |  | Zm00008a027385_T01 | Chr07 | 39457619 | 39459140 |
| *ZmPIP2d* |  | Zm00008a008651_T01 | Chr02 | 174181492 | 174182571 |

*** *Adopted from Sinbase (Sesamum indicum genome database, http://ocri-genomics.org/Sinbase/index.html).***
