# Supplementary material for: Combining genome-wide and transcriptome-wide analyses reveal the evolutionary conservation and functional diversity of aquaporins in cotton
Source: BMC Genomics. 2019 Jul 1;20:538. doi: 10.1186/s12864-019-5928-2 (PMC6604486; doi:10.1186/s12864-019-5928-2)
Supplement: Supplementary file 11 — Figure S5. Sequences alignment of GhPIP1;4h_At, GhPIP1;4i_At and GhPIP1;4j_At in three genome databases of G. hirsutum acc. TM-1. The DNA sequences of GhPIP1;4h_At, GhPIP1;4i_At and GhPIP1;4j_At were extracted from three released genome databases of G. hirsutum acc. TM-1, that from Zhang et al. (2015) named as NAU, Wang et al. (2019) as HAU and Hu et al. (2019) as ZJU, respectively (B). The location of genes on chromosomes was shown on the left (A) and the corresponding gene IDs were listed on the right with unknown or undivided genes marked in red (C). (PDF 71 kb) [file 12864_2019_5928_MOESM11_ESM.pdf]

Additional file 11: Figure S5. Sequences alignment of *GhPIP1;4h_At*, *GhPIP1;4i_At* and *GhPIP1;4j_At* in three genome databases of *G. hirsutum* L. acc. TM-1.

A **B** **C**

```
NAU|A10_99656268-99657211|+      ATGGAGGGGAAAGAAGAAGATGTTAGATTGGGAGCGAACAAGTTCTCAGAGAGGCAACCTATTGGGACAGCAGCCCAGTCCCAAGATGATAAGGATTACA  100  Gh_A10G2089
NAU|A10_99664641-99665584|-      ATGGAAGGGAAAGAAGAAGATGTTAGATTGGGAGCTAACAAGTTCTCAGAAAGGCAGCCTATTGGGACAGCAGCTCAGTCCCAAGATGATAAGGATTACA  100  Gh_A10G2090
NAU|A10_99675191-99676314|-      ATGGAAGGGAAAGAAGAAGATGTTAGATTGGGAGCTAACAAGTTCTCAGAAAGGCAACCTATTGGGACAGCAGCTCAGTCCCAAGATGATAAGGATTACA  100  Gh_A10G2091
ZJU|A10_113928852-113929978|+    ATGGAGGGGAAAGAAGAAGATGTTAGATTGGGAGCGAACAAGTTCTCAGAGAGGCAACCTATTGGGACAGCAGCCCAGTCCCAAGATGATAAGGATTACA  100  GH_A10G2583
ZJU|A10_113936978-113938102|-    ATGGAAGGGAAAGAAGAAGATGTTAGATTGGGAGCTAACAAGTTCTCAGAAAGGCAGCCTATTGGGACAGCAGCTCAGTCCCAAGATGATAAGGATTACA  100  GH_A10G2584
ZJU|A10_113947127-113948250|-    ATGGAAGGGAAAGAAGAAGATGTTAGATTGGGAGCTAACAAGTTCTCAGAAAGGCAACCTATTGGGACAGCAGCTCAGTCCCAAGATGATAAGGATTACA  100  GH_A10G2584
HAU|A10_113550255-113551380|+    ATGGAGGGGAAAGAAGAAGATGTTAGATTGGGAGCGAACAAGTTCTCAGAGAGGCAACCTATTGGGACAGCAGCCCAGTCCCAAGATGATAAGGATTACA  100  Ghir_A10G023690.1
HAU|A10_113558379-113559503|-    ATGGAAGGGAAAGAAGAAGATGTTAGATTGGGAGCTAACAAGTTCTCAGAAAGGCAGCCTATTGGGACAGCAGCTCAGTCCCAAGATGATAAGGATTACA  100  Ghir_A10G023700.1
HAU|A10_113569139-113570262|-    ATGGAAGGGAAAGAAGAAGATGTTAGATTGGGAGCTAACAAGTTCTCAGAAAGGCAACCTATTGGGACAGCAGCTCAGTCCCAAGATGATAAGGATTACA  100  Ghir_A10G023700.1

NAU|A10_99656268-99657211|+      CTGAGCCACCCCCAGCACCCTTGTTTGAGCCCAGTGAGTTGACTTCATGGTCGTTTTACCGAGCTGGAATCGCTGAGTTCGTTGCTACTTTCCTATTCTT  200
NAU|A10_99664641-99665584|-      CTGAGCCACCACCAGCACCCTTGTTTGAGCCTAGTGAGTTGACCTCATGGTCTTTTTACCGAGCTGGAATCGCTGAGTTCGTTGCTACTTTCCTTTTCTT  200
NAU|A10_99675191-99676314|-      CTGAGCCACCCCCAGCACCCTTGTTTGAGCCTAGTGAGTTGACTTCATGGTCGTTTTACCGAGCTGGAATCGCTGAGTTCGTTGCTACTTTCCTATTCTT  200
ZJU|A10_113928852-113929978|+    CTGAGCCACCCCCAGCACCCTTGTTTGAGCCCAGTGAGTTGACTTCATGGTCGTTTTACCGAGCTGGAATCGCTGAGTTCGTTGCTACTTTCCTATTCTT  200
ZJU|A10_113936978-113938102|-    CTGAGCCACCACCAGCACCCTTGTTTGAGCCTAGTGAGTTGACCTCATGGTCTTTTTACCGAGCTGGAATCGCTGAGTTCGTTGCTACTTTCCTTTTCTT  200
ZJU|A10_113947127-113948250|-    CTGAGCCACCCCCAGCACCCTTGTTTGAGCCTAGTGAGTTGACTTCATGGTCGTTTTACCGAGCTGGAATCGCTGAGTTCGTTGCTACTTTCCTATTCTT  200
HAU|A10_113550255-113551380|+    CTGAGCCACCCCCAGCACCCTTGTTTGAGCCCAGTGAGTTGACTTCATGGTCGTTTTACCGAGCTGGAATCGCTGAGTTCGTTGCTACTTTCCTATTCTT  200
HAU|A10_113558379-113559503|-    CTGAGCCACCACCAGCACCCTTGTTTGAGCCTAGTGAGTTGACCTCATGGTCTTTTTACCGAGCTGGAATCGCTGAGTTCGTTGCTACTTTCCTTTTCTT  200
HAU|A10_113569139-113570262|-    CTGAGCCACCCCCAGCACCCTTGTTTGAGCCTAGTGAGTTGACTTCATGGTCGTTTTACCGAGCTGGAATCGCTGAGTTCGTTGCTACTTTCCTATTCTT  200

NAU|A10_99656268-99657211|+      GTACATCTCGGTGTTGACTGTGATGGGAGTCCTTAAGGACAAAACCAAGTGTACAACTGTTGGGATTCAAGGGATTGCTTGGGCTTTTGGTGGAATGATC  300
NAU|A10_99664641-99665584|-      GTATATCTCGGTATTGACTGTGATGGGAGTCCTTAAGGACAAAACCAAGTGTACAACTGTTGGGATTCAAGGGATTGCTTGGGCTTTTGGTGGTATGATC  300
NAU|A10_99675191-99676314|-      GTACATCTCGGTGTTGACTGTGATGGGAGTCCTTAAGGACAAAACCAAGTGTACAACTGTTGGGATTCAAGGGATTGCTTGGGCTTTTGGTGGTATGATC  300
ZJU|A10_113928852-113929978|+    GTACATCTCGGTGTTGACTGTGATGGGAGTCCTTAAGGACAAAACCAAGTGTACAACTGTTGGGATTCAAGGGATTGCTTGGGCTTTTGGTGGAATGATC  300
ZJU|A10_113936978-113938102|-    GTATATCTCGGTATTGACTGTGATGGGAGTCCTTAAGGACAAAACCAAGTGTACAACTGTTGGGATTCAAGGGATTGCTTGGGCTTTTGGTGGTATGATC  300
ZJU|A10_113947127-113948250|-    GTACATCTCGGTGTTGACTGTGATGGGAGTCCTTAAGGACAAAACCAAGTGTACAACTGTTGGGATTCAAGGGATTGCTTGGGCTTTTGGTGGTATGATC  300
HAU|A10_113550255-113551380|+    GTACATCTCGGTGTTGACTGTGATGGGAGTCCTTAAGGACAAAACCAAGTGTACAACTGTTGGGATTCAAGGGATTGCTTGGGCTTTTGGTGGAATGATC  300
HAU|A10_113558379-113559503|-    GTATATCTCGGTATTGACTGTGATGGGAGTCCTTAAGGACAAAACCAAGTGTACAACTGTTGGGATTCAAGGGATTGCTTGGGCTTTTGGTGGTATGATC  300
HAU|A10_113569139-113570262|-    GTACATCTCGGTGTTGACTGTGATGGGAGTCCTTAAGGACAAAACCAAGTGTACAACTGTTGGGATTCAAGGGATTGCTTGGGCTTTTGGTGGTATGATC  300

NAU|A10_99656268-99657211|+      TTTGCTCTTGTTTACTGCACTGCTGGGATCTCAGGTAA-CTATATGATCTTCTTTATCCCTTGTTTGTTTCTCCGGAAAATGATAGAAAAGTTTAGTATT  399
NAU|A10_99664641-99665584|-      TTTGCTCTTGTTTACTGCACTGCTGGTATCTCAGGTAA-CTATATGATCTTCTTTATCCCTTGTTTGTTTCTCCGGAAAATGAAAGAAAAGTTTAGTATT  399
NAU|A10_99675191-99676314|-      TTTGCTCTTGTTTACTGCACTGCTGGTATCTCAGGTAAACTATATGATCAGCTTTATTCCTTGTTTGTTTCTCAGGAAAATGATAGAAAAGTATAGTATT  400
ZJU|A10_113928852-113929978|+    TTTGCTCTTGTTTACTGCACTGCTGGGATCTCAGGTAA-CTATATGATCTTCTTTATCACTCGTTTGTTTCTCCGGAAAATGATAGAAAAGTTTAGTATT  399
ZJU|A10_113936978-113938102|-    TTTGCTCTTGTTTACTGCACTGCTGGTATCTCAGGTAA-CTATATGATCTTCTTTATCCCTTGTTTGTTTCTCCGGAAAATGAAAGAAAAGTTTAGTATT  399
ZJU|A10_113947127-113948250|-    TTTGCTCTTGTTTACTGCACTGCTGGTATCTCAGGTAAACTATATGATCAGCTTTATTCCTTGTTTGTTTCTCAGGAAAATGATAGAAAAGTATAGTATT  400
HAU|A10_113550255-113551380|+    TTTGCTCTTGTTTACTGCACTGCTGGGATCTCAGGTAA-CTATATGATCTTCTTTATCACTCGTTTGTTTCTCCGGAAAATGATAGAAAAGTTTAGTATT  399
HAU|A10_113558379-113559503|-    TTTGCTCTTGTTTACTGCACTGCTGGTATCTCAGGTAA-CTATATGATCTTCTTTATCCCTTGTTTGTTTCTCCGGAAAATGAAAGAAAAGTTTAGTATT  399
HAU|A10_113569139-113570262|-    TTTGCTCTTGTTTACTGCACTGCTGGTATCTCAGGTAAACTATATGATCAGCTTTATTCCTTGTTTGTTTCTCAGGAAAATGATAGAAAAGTATAGTATT  400

NAU|A10_99656268-99657211|+      GATGAGTTATGGTGGTGTTGTAGGTGGTCATATCAATCCAGCGGTGACATTTGGGCTGTTCTTGGGAAGGAAATTGTCGTTGACAAGGGCAATATACTAC  499
NAU|A10_99664641-99665584|-      GATGAGTTATGGTGGTGTTGTAGGTGGTCATATCAATCCAGCGGTGACATTTGGGCTGTTCTTGGGAAGGAAATTGTCGTTGACAAGGGCAATATACTAC  499
NAU|A10_99675191-99676314|-      GATGAGTTATGGTGATGTTATAGGTGGTCATATAAATCCGGCGGTGACATTTGGGCTGTTCTTGGGAAGGAAATTGTCTTTGACAAGGGCAATATTCTAC  500
ZJU|A10_113928852-113929978|+    GATGAGTTATGGTGGTGTTGTAGGTGGTCATATCAATCCAGCGGTGACATTTGGGCTGTTCTTGGGAAGGAAATTGTCGTTGACAAGGGCAATATACTAC  499
ZJU|A10_113936978-113938102|-    GATGAGTTATGGTGGTGTTGTAGGTGGTCATATCAATCCAGCGGTGACATTTGGGCTGTTCTTGGGAAGGAAATTGTCGTTGACAAGGGCAATATACTAC  499
ZJU|A10_113947127-113948250|-    GATGAGTTATGGTGATGTTATAGGTGGTCATATAAATCCGGCGGTGACATTTGGGCTGTTCTTGGGAAGGAAATTGTCTTTGACAAGGGCAATATTCTAC  500
HAU|A10_113550255-113551380|+    GATGAGTTATGGTGGTGTTGTAGGTGGTCATATCAATCCAGCGGTGACATTTGGGCTGTTCTTGGGAAGGAAATTGTCGTTGACAAGGGCAATATACTAC  499
HAU|A10_113558379-113559503|-    GATGAGTTATGGTGGTGTTGTAGGTGGTCATATCAATCCAGCGGTGACATTTGGGCTGTTCTTGGGAAGGAAATTGTCGTTGACAAGGGCAATATACTAC  499
HAU|A10_113569139-113570262|-    GATGAGTTATGGTGATGTTATAGGTGGTCATATAAATCCGGCGGTGACATTTGGGCTGTTCTTGGGAAGGAAATTGTCTTTGACAAGGGCAATATTCTAC  500

NAU|A10_99656268-99657211|+      ATGGTGATGCAGTGCTTGGGAGCCATATGTGGGGCTGGTGTGGTTAAAGGGTTCATGGGGAAGACAAGGTATGGTGCTTTGGGTGGTGGAGCTAACTCTG  599
NAU|A10_99664641-99665584|-      ATGGTGATGCAGTGCTTGGGAGCCATATGTGGGGCTGGTGTGGTTAAAGGGTTCATGGGGAAGACAAGGTATGGTGCTTTGGGTGGTGGAGCTAACTCTG  599
NAU|A10_99675191-99676314|-      ATGGTGATGCAGTGCTTGGGAGCTATATGTGGGGCTGGTGTGGTTAAAGGGTTCATGGGGAAGACAAGGTATGGTGCTTTGGGTGGTGGAGCTAACTCTG  600
ZJU|A10_113928852-113929978|+    ATGGTGATGCAGTGCTTGGGAGCCATATGTGGGGCTGGTGTGGTTAAAGGGTTCATGGGGAAGACAAGGTATGGTGCTTTGGGTGGTGGAGCTAACTCTG  599
ZJU|A10_113936978-113938102|-    ATGGTGATGCAGTGCTTGGGAGCCATATGTGGGGCTGGTGTGGTTAAAGGGTTCATGGGGAAGACAAGGTATGGTGCTTTGGGTGGTGGAGCTAACTCTG  599
ZJU|A10_113947127-113948250|-    ATGGTGATGCAGTGCTTGGGAGCTATATGTGGGGCTGGTGTGGTTAAAGGGTTCATGGGGAAGACAAGGTATGGTGCTTTGGGTGGTGGAGCTAACTCTG  600
HAU|A10_113550255-113551380|+    ATGGTGATGCAGTGCTTGGGAGCCATATGTGGGGCTGGTGTGGTTAAAGGGTTCATGGGGAAGACAAGGTATGGTGCTTTGGGTGGTGGAGCTAACTCTG  599
HAU|A10_113558379-113559503|-    ATGGTGATGCAGTGCTTGGGAGCCATATGTGGGGCTGGTGTGGTTAAAGGGTTCATGGGGAAGACAAGGTATGGTGCTTTGGGTGGTGGAGCTAACTCTG  599
HAU|A10_113569139-113570262|-    ATGGTGATGCAGTGCTTGGGAGCTATATGTGGGGCTGGTGTGGTTAAAGGGTTCATGGGGAAGACAAGGTATGGTGCTTTGGGTGGTGGAGCTAACTCTG  600

NAU|A10_99656268-99657211|+      TGAATCATGGCTACACCAAGGGAGATGGGCTTGGTGCTGAAATTGTTGGCACCTTTGTGCTTGTTTACACTGTGTTCTCAGCCACTGATGCCAAGCGCAG  699
NAU|A10_99664641-99665584|-      TGAATCATGGCTACACCAAGGGAGATGGGCTTGGTGCTGAAATTGTTGGCACCTTTGTGCTTGTTTACACTGTGTTCTCAGCCACTGATGCCAAGCGCAG  699
NAU|A10_99675191-99676314|-      TGAATCATGGCTACACCAAGGGAGATGGGCTTGGTGCTGAAATTGTTGGCACCTTTGTGCTTGTTTACACTGTGTTCTCAGCCACTGACGCCAAGCGCAG  700
ZJU|A10_113928852-113929978|+    TGAATCATGGCTACACCAAGGGAGATGGGCTTGGTGCTGAAATTGTTGGCACCTTTGTGCTTGTTTACACTGTGTTCTCAGCCACTGATGCCAAGCGCAG  699
ZJU|A10_113936978-113938102|-    TGAATCATGGCTACACCAAGGGAGACGGGCTTGGTGCTGAAATTGTTGGCACCTTTGTGCTTGTTTACACTGTGTTCTCAGCCACTGATGCCAAGCGCAG  699
ZJU|A10_113947127-113948250|-    TGAATCATGGCTACACCAAGGGAGATGGGCTTGGTGCTGAAATTGTTGGTACCTTTGTTCTTGTTTACACTGTGTTCTCAGCCACTGACGCCAAGCGCAG  700
HAU|A10_113550255-113551380|+    TGAATCATGGCTACACCAAGGGAGATGGGCTTGGTGCTGAAATTGTTGGCACCTTTGTGCTTGTTTACACTGTGTTCTCAGCCACTGATGCCAAGCGCAG  699
HAU|A10_113558379-113559503|-    TGAATCATGGCTACACCAAGGGAGACGGGCTTGGTGCTGAAATTGTTGGCACCTTTGTGCTTGTTTACACTGTGTTCTCAGCCACTGATGCCAAGCGCAG  699
HAU|A10_113569139-113570262|-    TGAATCATGGCTACACCAAGGGAGATGGGCTTGGTGCTGAAATTGTTGGTACCTTTGTTCTTGTTTACACTGTGTTCTCAGCCACTGACGCCAAGCGCAG  700

NAU|A10_99656268-99657211|+      TGCCAGAGACTCTCACGTCCCTGTAAGTTCAATTTTCTTCAATTGAATCATTCGATTGTCTAAACCATATATGA----GATTTTTAACCATAACCATGTG  795
NAU|A10_99664641-99665584|-      TGCCAGAGACTCTCACGTCCCTGTAAGTTCAATTTTCTTCAATTGAATCATTCGATTGTCTAAACCATATATGA----GATTTTTAACCATAACCATGTG  795
NAU|A10_99675191-99676314|-      TGCCAGAGACTCTCACGTCCCTGTAAGTT------TCATCAAATGAATGATTGAATTGTTTAAACCATTAAAACCCAGATTTTTAACCATGACCATGTG  794
ZJU|A10_113928852-113929978|+    TGCCAGAGACTCTCACGTCCCTGTAAGTTCAATTTTCTTCAATTGAATCATTCGATTGTCTAAACCATATATGA----GATTTTTAACCATAACCATGTG  795
ZJU|A10_113936978-113938102|-    TGCCAGAGACTCTCACGTCCCTGTAAGTTCAATTTTCTTCAATTGAATCATTCGATTGTCTAAACCATATATGA----GATTTTTAACCATAACCATGTG  795
```

```
ZJU|A10_113947127-113948250|-   TGCCAGAGACTCTCACGTCCCTGTAAGTT------TCATCAAATGAATGATTGAATTGTTTAAACCATTAAAAACCCAGATTTTTAACCATGACCATGTG   794
HAU|A10_113550255-113551380|+   TGCCAGAGACTCTCACGTCCCTGTAAGTTCAATTTTCTTCAATTGAATCATTCGATTGTCTAAACCATATATGA----GATTTTTAACCATAACCATGTG   795
HAU|A10_113558379-113559503|-   TGCCAGAGACTCTCACGTCCCTGTAAGTTCAATTTTCTTCAATTGAATCATTCGATTGTCTAAACCATATATGA----GATTTTTAACCATAACCATGTG   795
HAU|A10_113569139-113570262|-   TGCCAGAGACTCTCACGTCCCTGTAAGTT------TCATCAAATGAATGATTGAATTGTTTAAACCATTAAAAACCCAGATTTTTAACCATGACCATGTG   794

NAU|A10_99656268-99657211|+     TTTGGTAGATTCTGGCACCATTGCCAATTGGGTTCGCAGTGTTCTTGGTGCACTTAGCCACCATCCCAATCACAGGAACCGGTATTAACCCAGCTCGTAG   895
NAU|A10_99664641-99665584|-     TTTGGTAGATTCTGGCACCATTGCCAATTGGGTTCGCAGTGTTCTTGGTGCACTTAGCCACCATCCCAATCACAGGAACCGGTATTAACCCAGCTCGTAG   895
NAU|A10_99675191-99676314|-     TTTGGTAGATTTTGGCACCATTGCCAATTGGGTTCGCAGTGTTCTTGGTACACTTAGCCACCATCCCAATCACAGGAACTGGTATTAACCCAGCTCGTAG   894
ZJU|A10_113928852-113929978|+   TTTGGTAGATTCTGGCACCATTGCCAATTGGGTTCGCAGTGTTCTTGGTGCACTTAGCCACCATCCCAATCACAGGAACCGGTATTAACCCAGCTCGTAG   895
ZJU|A10_113936978-113938102|-   TTTGGTAGATTCTGGCACCATTGCCAATTGGGTTCGCAGTGTTCTTGGTGCACTTAGCCACCATCCCAATCACAGGAACCGGTATTAACCCAGCTCGTAG   895
ZJU|A10_113947127-113948250|-   TTTGGTAGATTTTGGCACCATTGCCAATTGGGTTCGCAGTGTTCTTGGTACACTTAGCCACCATCCCAATCACAGGAACTGGTATTAACCCAGCTCGTAG   894
HAU|A10_113550255-113551380|+   TTTGGTAGATTCTGGCACCATTGCCAATTGGGTTCGCAGTGTTCTTGGTGCACTTAGCCACCATCCCAATCACAGGAACCGGTATTAACCCAGCTCGTAG   895
HAU|A10_113558379-113559503|-   TTTGGTAGATTCTGGCACCATTGCCAATTGGGTTCGCAGTGTTCTTGGTGCACTTAGCCACCATCCCAATCACAGGAACCGGTATTAACCCAGCTCGTAG   895
HAU|A10_113569139-113570262|-   TTTGGTAGATTTTGGCACCATTGCCAATTGGGTTCGCAGTGTTCTTGGTACACTTAGCCACCATCCCAATCACAGGAACTGGTATTAACCCAGCTCGTAG   894

NAU|A10_99656268-99657211|+     TCTTGGTGCAGCCATCATTTTCAACAAGGACAAGGGCTGGGATGACCAT---------------------------------------------------   944
NAU|A10_99664641-99665584|-     TCTTGGTGCAGCCATCATTTTCAACAAGGACAAGGGCTGGGATGACCAT---------------------------------------------------   944
NAU|A10_99675191-99676314|-     CCTTGGTGCGGCCATCATTTTCAACAAGGACAAGGGATGGGATGACCATGTAAGT--CAATTGTTTCACCATTTTTCACTATTATTATTTT-TAATCATG   991
ZJU|A10_113928852-113929978|+   TCTTGGTGCAGCCATCATTTTCAACAAGGACAAGGGCTGGGATGACCATGTAAGCCCCAATTCTTTCGCCATCTT-CACTATTATTTTTTTCTAACTATG   994
ZJU|A10_113936978-113938102|-   TCTTGGTGCAGCCATCATTTTCAACAAGGACAAGGGCTGGGATGACCATGTAAGCCCCAATTCTTTCGCCATCTT-CACTATTATTTTTTTCTAACTATG   994
ZJU|A10_113947127-113948250|-   CCTTGGTGCGGCCATCATTTTCAACAAGGACAAGGGCTGGGATGACCATGTAAGT--CAATTGTTTCACCATTTTTCACTATTATTATTTT-TAATCATG   991
HAU|A10_113550255-113551380|+   TCTTGGTGCAGCCATCATTTTCAACAAGGACAAGGGCTGGGATGACCATGTAAGCCCCAATTCTTTCGCCATCT--CACTATTATTTTTTTCTAACTATG   993
HAU|A10_113558379-113559503|-   TCTTGGTGCAGCCATCATTTTCAACAAGGACAAGGGCTGGGATGACCATGTAAGCCCCAATTCTTTCGCCATCTT-CACTATTATTTTTTTCTAACTATG   994
HAU|A10_113569139-113570262|-   CCTTGGTGCGGCCATCATTTTCAACAAGGACAAGGGCTGGGATGACCATGTAAGT--CAATTGTTTCACCATTTTTCACTATTATTATTTT-TAATCATG   991

NAU|A10_99656268-99657211|+     ----------------------------------------------------------------------------------------------------   944
NAU|A10_99664641-99665584|-     ----------------------------------------------------------------------------------------------------   944
NAU|A10_99675191-99676314|-     AATAATATACTTATCTACTTTGGGTCTGGTTTGATTGCAGTGGATTTTCTGGGTGGGTCCATTCATTGGTGCAGCACTAGCAGCACTTTACCACGTTGTT   1091
ZJU|A10_113928852-113929978|+   AATGATATACTTATCTACTTTGGGTCATGTTTTGCTGCAGTGGATTTTCTGGGTGGGTCCATTCATTGGAGCAGCACTAGCAGCACTCTACCACGTTGTT   1094
ZJU|A10_113936978-113938102|-   AATGATAT--TTATCTACTTTGGGTCATGTTTTACTGCAGTGGATCTTCTGGGTGGGTCCATTCATTGGAGCAGCACTAGCAGCACTCTACCATGTTGTT   1092
ZJU|A10_113947127-113948250|-   AATAATATACTTATCTACTTTGGGTCTGGTTTGATTGCAGTGGATTTTCTGGGTGGGTCCATTCATTGGTGCAGCACTAGCAGCACTTTACCACGTTGTT   1091
HAU|A10_113550255-113551380|+   AATGATATACTTATCTACTTTGGGTCATGTTTTGCTGCAGTGGATTTTCTGGGTGGGTCCATTCATTGGAGCAGCACTAGCAGCACTCTACCACGTTGTT   1093
HAU|A10_113558379-113559503|-   AATGATAT--TTATCTACTTTGGGTCATGTTTTACTGCAGTGGATCTTCTGGGTGGGTCCATTCATTGGAGCAGCACTAGCAGCACTCTACCATGTTGTT   1092
HAU|A10_113569139-113570262|-   AATAATATACTTATCTACTTTGGGTCTGGTTTGATTGCAGTGGATTTTCTGGGTGGGTCCATTCATTGGTGCAGCACTAGCAGCACTTTACCACGTTGTT   1091

NAU|A10_99656268-99657211|+     ---------------------------------   944
NAU|A10_99664641-99665584|-     ---------------------------------   944
NAU|A10_99675191-99676314|-     GTGATCAGAGCCATTCCTTTCAAATCAAAATGA   1124
ZJU|A10_113928852-113929978|+   GTTATCAGGGCCATTCCTTTCAAGTCAAAATGA   1127
ZJU|A10_113936978-113938102|-   GTGATCAGAGCCATTCCTTTCAAATCAAAATGA   1125
ZJU|A10_113947127-113948250|-   GTGATCAGAGCCATTCCTTTCAAATCAAAATGA   1124
HAU|A10_113550255-113551380|+   GTTATCAGGGCCATTCCTTTCAAGTCAAAATGA   1126
HAU|A10_113558379-113559503|-   GTGATCAGAGCCATTCCTTTCAAATCAAAATGA   1125
HAU|A10_113569139-113570262|-   GTGATCAGAGCCATTCCTTTCAAATCAAAATGA   1124
```
