# Supplementary material for: Combining genome-wide and transcriptome-wide analyses reveal the evolutionary conservation and functional diversity of aquaporins in cotton
Source: BMC Genomics. 2019 Jul 1;20:538. doi: 10.1186/s12864-019-5928-2 (PMC6604486; doi:10.1186/s12864-019-5928-2)
Supplement: Supplementary file 12 — Figure S6. Sequences alignment of GhPIP2;2a_At, GhPIP2;2e_At, GhPIP2;2a_Dt and GhPIP2;2e_Dt in three genome databases of G. hirsutum acc. TM-1. The DNA sequences of GhPIP2;2a_At, GhPIP2;2e_At, GhPIP2;2a_Dt and GhPIP2;2e_Dt were extracted from three released genome databases of G. hirsutum acc. TM-1, that from Zhang et al. (2015) named as NAU, Wang et al. (2019) as HAU and Hu et al. (2019) as ZJU, respectively (B). The location of genes on chromosomes was shown on the left (A) and the corresponding gene IDs were listed on the right with unknown or undivided genes marked in red (C). (PDF 71 kb) [file 12864_2019_5928_MOESM12_ESM.pdf]

Additional file 12: Figure S6. Sequences alignment of *GhPIP2;2a_At, GhPIP2;2e_At, GhPIP2;2a_Dt and GhPIP2;2e_Dt* in three genome databases of *G. hirsutum* L. acc. TM-1.

A | **B** | **C**

```
NAU|A01_98366726-98367857|+     ATGGCTAAGGATGTTGAGGTTGGTGGTGAGTTCCAAGCCAAGGACTACCATGATCCTCCACCAGCTCCATTGGTAGATGCCCAAGAGTTAACTCAGTGGT 100 Gh_A01G1844
NAU|A01_98361882-98363012|+     ATGGCTAAGGATGTTGAGGTTGGTGGTGAGTTCCAAGCCAAGGACTACCATGATCCTCCACCAGCTCCATTGGTAGATGCCCAAGAGTTAACTCAGTGGT 100 Gh_A01G1843
NAU|D01_59898611-59899741|+     ATGGCTAATGATGTTGAGGTTGGTAGTGAGTTCCAAGCCAAGGACTACCATGATCCTCCACCAGCTCCATTGGTAGATGCCCAAGAGTTAACTCAGTGGT 100 Gh_D01G2086
NAU|D01_59890054-59891184|+     ATGGCCAAGGATGTTGAGGTTGGTGGTGAGTTCCAACCCAAGGACTACCATGATCCTCCACCAGCTCCATTTGTAGATGCCTAAGAGTTAACTCAGTGGT 100 Gh_D01G2085
ZJU|A01_116473274-116474405|+   ATGGCTAAGGATGTTGAGGTTGGTGGTGAGTTCCAAGCCAAGGACTACCATGATCCTCCACCAGCTCCATTGGTAGATGCCCAAGAGTTAACTCAGTGGT 100 GH_A01G2321
ZJU|A01_116468430-116469560|+   ATGGCTAAGGATGTTGAGGTTGGTGGTGAGTTCCAAGCCAAGGACTACCATGATCCTCCACCAGCTCCATTGGTAGATGCCCAAGAGTTAACTCAGTGGT 100 GH_A01G2320
ZJU|D01_63074368-63075498|+     ATGGCCAAGGATGTTGAGGTTGGTGGTGAGTTCCAACCCAAGGACTACCATGATCCTCCACCAGCTCCATTTGTAGATGCCTAAGAGTTAACTCAGTGGT 100 GH_D01G2400
ZJU|scaffold_D01_84-385/46-787  ATGGCTAATGATGTTGAGGTTGGTAGTGAGTTCCAAGCCAAGGACTACCATGATCCTCCACCAGCTCCATTGGTAGATGCCCAAGAGTTAACTCAGTGGT 100 Unknown
HAU|A01_116031840-116032971|+   ATGGCTAAGGATGTTGAGGTTGGTGGTGAGTTCCAAGCCAAGGACTACCATGATCCTCCACCAGCTCCATTGGTAGATGCCCAAGAGTTAACTCAGTGGT 100 Ghir_A01G020720.1
HAU|A01_116027001-116028131|+   ATGGCTAAGGATGTTGAGGTTGGTGGTGAGTTCCAAGCCAAGGACTACCATGATCCTCCACCAGCTCCATTGGTAGATGCCCAAGAGTTAACTCAGTGGT 100 Ghir_A01G020720.1
HAU|D01_61617884-61619012|+     ATGGCTAATGATGTTGAGGTTGGTAGTGAGTTCCAAGCCAAGGACTACCATGATCCTCCACCAGCTCCATTGGTAGATGCCCAAGAGTTAACTCAGTGGT 100 Ghir_D01G022260.1
HAU|D01_61609329-61610459|+     ATGGCCAAGGATGTTGAGGTTGGTGGTGAGTTCCAACCCAAGGACTACCATGATCCTCCACCAGCTCCATTTGTAGATGCCTAAGAGTTAACTCAGTGGT 100 Ghir_D01G022270.1

NAU|A01_98366726-98367857|+     CTTTTTATAGGGCTATAATAGCTGAATTCATTGCCACCCTTTTGTTTTTATACATCACTGTGTTAACAGTGATTGGATACAAGAGCCAAGTTGATCCAGA 200
NAU|A01_98361882-98363012|+     CCTTTTACAGGGCTATAATAGCTGAGTTCATTGCCACCCTTTTGTTTTTATACATCACTGTGTTAACAGTGATCGGATACAAGAGCCAAGTTGATCCAGA 200
NAU|D01_59898611-59899741|+     CTTTTTATAGGGCTATAATAGCTGAGTTCATTGCCACCCTTTTGTTTTTATACATCACTGTGTTAACAGTGATTGGATACAAGAGCCAAGTTGATCCAGA 200
NAU|D01_59890054-59891184|+     CTTTTTATAGGGCTATAATAGCTGAGTTCATTGCCACCCTTTTGTTTTTATACATCACTGTGTTAACAGTGATTGGATACAAGAGCCAAGTTGATCCAGA 200
ZJU|A01_116473274-116474405|+   CTTTTTATAGGGCTATAATAGCTGAATTCATTGCCACCCTTTTGTTTTTATACATCACTGTGTTAACAGTGATTGGATACAAGAGCCAAGTTGATCCAGA 200
ZJU|A01_116468430-116469560|+   CCTTTTACAGGGCTATAATAGCTGAGTTCATTGCCACCCTTTTGTTTTTATACATCACTGTGTTAACAGTGATCGGATACAAGAGCCAAGTTGATCCAGA 200
ZJU|D01_63074368-63075498|+     CTTTTTATAGGGCTATAATAGCTGAGTTCATTGCCAGCCTTTTGTTTTTATACATCACTGTGTTAACAGTGATTGGATACAAGAGCCAAGTTGATCCAGA 200
ZJU|scaffold_D01_84-385/46-787  CTTTTTATAGGGCTATAATAGCTGAGTTCATTGCCACCCTTTTGTTTTTATACATCACTGTGTTAACAGTGATCGGATACAAGAGCCAAGTTGATCCAGA 200
HAU|A01_116031840-116032971|+   CTTTTTATAGGGCTATAATAGCTGAATTCATTGCCACCCTTTTGTTTTTATACATCACTGTGTTAACAGTGATTGGATACAAGAGCCAAGTTGATCCAGA 200
HAU|A01_116027001-116028131|+   CCTTTTACAGGGCTATAATAGCTGAGTTCATTGCCACCCTTTTGTTTTTATACATCACTGTGTTAACAGTGATCGGATACAAGAGCCAAGTTGATCCAGA 200
HAU|D01_61617884-61619012|+     CTTTTTATAGGGCTATAATAGCTGAGTTCATTGCCACCCTTTTGTTTTTATACATCACTGTGTTAACAGTGATCGGATACAAGAGCCAAGTTGATCCAGA 200
HAU|D01_61609329-61610459|+     CTTTTTATAGGGCTATAATAGCTGAGTTCATTGCCAGCCTTTTGTTTTTATACATCACTGTGTTAACAGTGATTGGATACAAGAGCCAAGTTGATCCAGA 200

NAU|A01_98366726-98367857|+     TAAGGGTGGTCAAGATTGTGATGGTGTTGGCATTCTTGGTATTGCTTGGGCCTTTGGTGGCATGATTTTCATCCTTGTTTACTGCACTGCTGGTATCTCA 300
NAU|A01_98361882-98363012|+     TAAGGGTGGTCAAGATTGTGATGGTGTTGGCATTCTTGGTATTGCTTGGGCCTTTGGTGGCATGATTTTCATCCTTGTTTACTGCACTGCTGGTATCTCA 300
NAU|D01_59898611-59899741|+     TAAGGGTGGTCAAGATTGTGATGGTGTTGGCATTCTTGGTATTGCTTGGGCCTTTGGTGGCATGATTTTCATCCTTGTTTACTGCACTGCTGGTATCTCA 300
NAU|D01_59890054-59891184|+     TAAGGGTGGTCAAGATTGTGATGGTGTTGGCATTCTTGGTATTGCTTGGGCCTTTGGTGGCATGATTTTCATCCTTGTTTACTGCACTGCTGGTATCTCA 300
ZJU|A01_116473274-116474405|+   TAAGGGTGGTCAAGATTGTGATGGTGTTGGCATTCTTGGTATTGCTTGGGCCTTTGGTGGCATGATTTTCATCCTTGTTTACTGCACTGCTGGTATCTCA 300
ZJU|A01_116468430-116469560|+   TAAGGGTGGTCAAGATTGTGATGGTGTTGGCATTCTTGGTATTGCTTGGGCCTTTGGTGGCATGATTTTCATCCTTGTTTACTGCACTGCTGGTATCTCA 300
ZJU|D01_63074368-63075498|+     TAAGGGTGGTCAAGATTGTGATGGTGTTGGCATTCTTGGTATTGCTTGGGCCTTTGGTGGCATGATTTTCATCCTTGTTTACTGCACTGCTGGTATCTCA 300
ZJU|scaffold_D01_84-385/46-787  TAAGGGTGGTCAAGATTGTGATGGTGTTGGCATTCTTGGTATTGCTTGGGCCTTTGGTGGCATGATTTTCATCCTTGTTTACTGCACTGCTGGTATCTCA 300
HAU|A01_116031840-116032971|+   TAAGGGTGGTCAAGATTGTGATGGTGTTGGCATTCTTGGTATTGCTTGGGCCTTTGGTGGCATGATTTTCATCCTTGTTTACTGCACTGCTGGTATCTCA 300
HAU|A01_116027001-116028131|+   TAAGGGTGGTCAAGATTGTGATGGTGTTGGCATTCTTGGTATTGCTTGGGCCTTTGGTGGCATGATTTTCATCCTTGTTTACTGCACTGCTGGTATCTCA 300
HAU|D01_61617884-61619012|+     TAAGGGTGGTCAAGATTGTGATGGTGTTGGCATTCTTGGTATTGCTTGGGCCTTTGGTGGCATGATTTTCATCCTTGTTTACTGCACTGCTGGTATCTCA 300
HAU|D01_61609329-61610459|+     TAAGGGTGGTCAAGATTGTGATGGTGTTGGCATTCTTGGTATTGCTTGGGCCTTTGGTGGCATGATTTTCATCCTTGTTTACTGCACTGCTGGTATCTCA 300

NAU|A01_98366726-98367857|+     GGTAATAATATCTTATACACACTCGTTGTTGAACGATATGGTTAAAACTTAAAAGGGTGTTTTCTTAGCATGTTTGTTTGGTGATATGTAGGGGGACATA 400
NAU|A01_98361882-98363012|+     GGTAATAATATCTTATACACACTCCTTGTTGAACGATATGGTTAAAACTTAAAAGGGTGTTTTCTTAGCATGTTTGTTTGGTGATATGTAGGGGGACATA 400
NAU|D01_59898611-59899741|+     GGTAATAATATCTTATACACACTCCTTGTTGAACGATATGGTTAAAACTTAAAAGGGTGTTTTCTTAGCATGTTTGTTTGGTGATATGTAGGGGGACATA 400
NAU|D01_59890054-59891184|+     GGTAATAATATCTTATACACACTCCTTGTTGAACGATATGGTTAAAACTTAAAAGGGTGTTTTCTTAGCATGTTTGTTTGGTGATATGTAGGGGGACATA 400
ZJU|A01_116473274-116474405|+   GGTAATAATATCTTATACACACTCGTTTTTGAACGATATGGTTAAAACTTAAAAGGGTGTTTTCTTAGCATGTTTGTTTGGTGATGTGTAGGGGGACATA 400
ZJU|A01_116468430-116469560|+   GGTAATAATATCTTATACACACTCGTTTTTGAACGATATGGTTAAAACTTTAAAGGGTGTTTTCTTAGCATGTTTATTTGGTGATATGTAGGGGGACATA 400
ZJU|D01_63074368-63075498|+     GGTAATAATATCTTATACACACTCCTTGTTGAACGATATGGTTAAAACTTAAAAGGGTGTTTTCTTAGCATGTTTGTTTGGTGATGTGTAGGGGGACATA 400
ZJU|scaffold_D01_84-385/46-787  GG---------------------------------------------------------------------------------------AGGGGGACATA 313
HAU|A01_116031840-116032971|+   GGTAATAATATCTTATACACACTCGTTTTTGAACGATATGGTTAAAACTTAAAAGGGTGTTTTCTTAGCATGTTTGTTTGGTGATGTGTAGGGGGACATA 400
HAU|A01_116027001-116028131|+   GGTAATAATATCTTATACACACTCGTTTTTGAACGATATGGTTAAAACTTTAAAGGGTGTTTTCTTAGCATGTTTATTTGGTGATATGTAGGGGGACATA 400
HAU|D01_61617884-61619012|+     GGTAATAATATCTTATACACACTCCTTGTTGAACGATATGGTTAAAACTTAAAAGGGTGTTTTCTTAGCATGTTTGTTTGGTGATATGTAGGGGGACATA 400
HAU|D01_61609329-61610459|+     GGTAATAATATCTTATACACACTCCTTGTTGAACGATATGGTTAAAACTTAAAAGGGTGTTTTCTTAGCATGTTTGTTTGGTGATGTGTAGGGGGACATA 400

NAU|A01_98366726-98367857|+     TCAACCCAGCAGTGACCTTTGGGCTTTTCTTGGCTCGTAAGGTATCATTGGTTCGAGCCATACTTTATATGGCTGCTCAATGTTTAGGAGCCATTTGTGG 500
NAU|A01_98361882-98363012|+     TCAACCCAGCAGTGACCTTTGGGCTTTTCTTGGCTCGTAAGGTATCATTGGTTCGAGCCATACTTTATATGGCTGCTCAATGTTTAGGAGCCATTTGTGG 500
NAU|D01_59898611-59899741|+     TCAACCCAGCAGTGACCTTTGGGCTTTTCTTGGCTCGTAAGGTATCATTGGTTCGAGCCATACTTTATATGGCTGCTCAATGTTTAGGAGCCATTTGTGG 500
NAU|D01_59890054-59891184|+     TCAACCCAGCAGTGACCTTTGGGCTTTTCTTGGCTCGTAAGGTATCATTGGTTCGAGCCATACTTTATATGGCTGCTCAATGTTTAGGAGCCATTTGTGG 500
ZJU|A01_116473274-116474405|+   TCAACCCAGCAGTGACCTTTGGGCTTTTCTTGGCTCGTAAGGTATCATTGGTTCGAGCCATACTTTATATGGCTGCTCAATGTTTAGGAGCCATTTGTGG 500
ZJU|A01_116468430-116469560|+   TCAACCCAGCAGTGACCTTTGGGCTTTTCTTGGCTCGTAAGGTATCATTGGTTCGAGCCATACTTTATATGGCTGCTCAATGTTTAGGAGCCATTTGTGG 500
ZJU|D01_63074368-63075498|+     TCAACCCAGCAGTGACCTTTGGGCTTTTCTTGGCTCGTAAGGTATCATTGGTTCGAGCCATACTTTATATGGCTGCTCAATGTTTAGGAGCCATTTGTGG 500
ZJU|scaffold_D01_84-385/46-787  TCAACCCAGCAGTGACCTTTGGGCTTTTCTTGGCTCGTAAGGTATCATTGGTTCGAGCCATACTTTATATGGCTGCTCAATGTTTAGGAGCCATTTGTGG 413
HAU|A01_116031840-116032971|+   TCAACCCAGCAGTGACCTTTGGGCTTTTCTTGGCTCGTAAGGTATCATTGGTTCGAGCCATACTTTATATGGCTGCTCAATGTTTAGGAGCCATTTGTGG 500
HAU|A01_116027001-116028131|+   TCAACCCAGCAGTGACCTTTGGGCTTTTCTTGGCTCGTAAGGTATCATTGGTTCGAGCCATACTTTATATGGCTGCTCAATGTTTAGGAGCCATTTGTGG 500
HAU|D01_61617884-61619012|+     TCAACCCAGCAGTGACCTTTGGGCTTTTCTTGGCTCGTAAGGTATCATTGGTTCGAGCCATACTTTATATGGCTGCTCAATGTTTAGGAGCCATTTGTGG 500
HAU|D01_61609329-61610459|+     TCAACCCAGCAGTGACCTTTGGGCTTTTCTTGGCTCGTAAGGTATCATTGGTTCGAGCCATACTTTATATGGCTGCTCAATGTTTAGGAGCCATTTGTGG 500

NAU|A01_98366726-98367857|+     GTGTGGGTTAGTCAAGGCATTCCAAAAGAGTTATTACAACAAGTATGGTGGAGGAGCTAATAGCTTGGCTGATGGATACAGCACTGGCACTGGTTTGGCT 600
NAU|A01_98361882-98363012|+     GTGTGGGTTAGTCAAGGCATTCCAAAAGAGTTATTACAACAAGTATGGTGGAGGAGCTAATAGCTTGGCTGATGGATACAGCACTGGCACTGGTTTGGCT 600
NAU|D01_59898611-59899741|+     GTGTGGGTTAGTCAAGGCATTCCAAAAGAGTTATTACAACAAGTATGGTGGAGGAGCTAATAGCTTGGCTGATGGATACAGCACTGGCACTGGTTTGGCT 600
NAU|D01_59890054-59891184|+     GTGTGGGTTAGTCAAGGCATTCCAAAAGAGTTATTACAACAAGTATGGTGGAGGAGCTAATAGCTTGGCTGATGGATACAGCACTGGCACTGGTTTGGCT 600
ZJU|A01_116473274-116474405|+   GTGTGGGTTAGTCAAGGCATTCCAAAAGAGTTATTACAACAAGTATGGTGGAGGAGCTAATAGCTTGGCTGATGGATACAGCACTGGCACTGGTTTGGCT 600
ZJU|A01_116468430-116469560|+   GTGTGGGTTAGTCAAGGCTTTCCAAAAGAGTTATTACAACAAGTATGGTGGAGGAGCTAATAGCTTGGCTGATGGATACAGCACTGGCACTGGTTTGGCT 600
ZJU|D01_63074368-63075498|+     GTGTGGGTTAGTCAAGGCATTCCAAAAGAGTTATTACAACAAGTATGGTGGAGGAGCTAATAGCTTGGCTGATGGATACAGCACTGGCACTGGTTTGGCT 600
ZJU|scaffold_D01_84-385/46-787  GTGTGGGTTAGTCAAGGCATTCCAAAAGAGTTATTACAACAAGTATGGTGGAGGAGCTAATAGCTTGGCTGATGGATACAGCACTGGCACTGGTTTGGCT 513
HAU|A01_116031840-116032971|+   GTGTGGGTTAGTCAAGGCATTCCAAAAGAGTTATTACAACAAGTATGGTGGAGGAGCTAATAGCTTGGCTGATGGATACAGCACTGGCACTGGTTTGGCT 600
HAU|A01_116027001-116028131|+   GTGTGGGTTAGTCAAGGCTTTCCAAAAGAGTTATTACAACAAGTATGGTGGAGGAGCTAATAGCTTGGCTGATGGATACAGCACTGGCACTGGTTTGGCT 600
HAU|D01_61617884-61619012|+     GTGTGGGTTAGTCAAGGCATTCCAAAAGAGTTATTACAACAAGTATGGTGGAGGAGCTAATAGCTTGGCTGATGGATACAGCACTGGCACTGGTTTGGCT 600
HAU|D01_61609329-61610459|+     GTGTGGGTTAGTCAAGGCATTCCAAAAGAGTTATTACAACAAGTATGGTGGAGGAGCTAATAGCTTGGCTGATGGATACAGCACTGGCACTGGTTTGGCT 600

NAU|A01_98366726-98367857|+     GCTGAAATTATAGGCACGTTTGTGCTTGCTTACACTGTATTATCTGCAACTGATCCCAAAAGAAACGCAAGAGATTCTCATGTTCCTGTATGTCTCTTAA 700
NAU|A01_98361882-98363012|+     GCTGAAATTATTGGCACATTTGTGCTTGTTTACACTGTATTCTCTGCAACTGATCCCAAAAGAAACGCAAGAGATTCTCATGTTCCTGTATGTCTCTTAA 700
```

```
NAU|D01_59898611-59899741|+    GCTGAAATTATTGGCACATTTGTGCTTGTTTACACTGTATTCTCTGCAACTGATCCCAAAAGAAACGCAAGAGATTCTCATGTTCCTGTATGTCTCTTAA  700
NAU|D01_59890054-59891184|+    GCTGAAATTATTGGCACATTTGTGCTTGTTTACACTGTATTCTCTGCAACTGATCCCAAAAGAAACGCAAGAGATTCTCATGTTCCTGTATGTCTCTTAA  700
ZJU|A01_116473274-116474405|+  GCTGAAATTATAGGCACGTTTGTGCTTGCTTACACTGTATTATCTGCAACTGATCCCAAAAGAAACGCAAGAGATTCTCATGTTCCTGTATGTCTCTTAA  700
ZJU|A01_116468430-116469560|+  GCTGAAATTATTGGCACATTTGTGCTTGTTTACACTGTTTTCTCTGCAACTGATCCCAAAAGAAACGCAAGAGATTCTCATGTTCCTGTATGTCTCTTAA  700
ZJU|D01_63074368-63075498|+    GCTGAAATTATTGGCACATTTGTGCTTGTTTACACTGTATTCTCTGCAACTGATCCCAAAAGAAACGCAAGAGATTCTCATGTTCCTGTATGTCTCTTAA  700
ZJU|scaffold_D01_84-385/46-787 GCTGAAATTATTGGCACATTTGTGCTTGTTTACACTGTATTCTCTGCAACTGATCCCAAAAGAAACGCAAGAGATTCTCATGTTCCTGTATGTCTCTTAA  613
HAU|A01_116031840-116032971|+  GCTGAAATTATAGGCACGTTTGTGCTTGCTTACACTGTATTATCTGCAACTGATCCCAAAAGAAACGCAAGAGATTCTCATGTTCCTGTATGTCTCTTAA  700
HAU|A01_116027001-116028131|+  GCTGAAATTATTGGCACATTTGTGCTTGTTTACACTGTTTTCTCTGCAACTGATCCCAAAAGAAACGCAAGAGATTCTCATGTTCCTGTATGTCTCTTAA  700
HAU|D01_61617884-61619012|+    GCTGAAATTATTGGCACATTTGTGCTTGTTTACACTGTATTCTCTGCAACTGATCCCAAAAGAAACGCAAGAGATTCTCATGTTCCTGTATGTCTCTTAA  700
HAU|D01_61609329-61610459|+    GCTGAAATTATTGGCACATTTGTGCTTGTTTACACTGTATTCTCTGCAACTGATCCCAAAAGAAACGCAAGAGATTCTCATGTTCCTGTATGTCTCTTAA  700

NAU|A01_98366726-98367857|+    TTCAATCCAACACTACACACAAGTTCATTGACAAGTATGTTTTTAGGTTCTAATTTTTTCTACATTTTGAAGGTGTTGGCACCACTTCCCATTGGGTTTA  800
NAU|A01_98361882-98363012|+    TTCAATCCAACACCACACACAAGTTCATTGACAAGTATGTTTTTAGGGACTAATTTTTTCTACATCTTGAAGGTGTTGGCACCACTTCCCATTGGGTTTG  800
NAU|D01_59898611-59899741|+    TTCAATCCAACACCACACACAAGTTCATTGACAAGTATGTTTTTAGGGACTAATTTTTTCTACATTTTGAAGGTGTTGGCACCACTTCCCATTGGGTTTG  800
NAU|D01_59890054-59891184|+    TTCAATCCAACACCACACACAAGTTCATTGACAAGTATGTTTTTAGGGACTAATTTTTTCTACATTTTGAAGGTGTTGGCACCACTTCCCATTGGGTTTG  800
ZJU|A01_116473274-116474405|+  TTCAATCCAACACTACACACAAGTTCATTGACAAGTATGTTTTTAGGTTCTAATTTTTTCTACATTTTGAAGGTGTTGGCACCACTTCCCATTGGGTTTA  800
ZJU|A01_116468430-116469560|+  TTCAATCCAACACCACACACAAGTTCATTGACAAGTATGTTTTTAGGGACTAATTTTTTCTACATCTTGAAGGTGTTGGCACCACTTCCCATTGGGTTTG  800
ZJU|D01_63074368-63075498|+    TTCAATCCAACACCACACACAAGTTCATTGACAAGTATGTTTTTAGGGACTAATTTTTTCTACATTTTGAAGGTGTTGGCACCACTTCCCATTGGGTTTG  800
ZJU|scaffold_D01_84-385/46-787 TTCAATCCAACACCACACACAAGTTCATTGACAAGTATGTTTTTAGGGACTAATTTTTTCTACATTTTGAAGGTGTTGGCACCACTTCCCATTGGGTTTG  713
HAU|A01_116031840-116032971|+  TTCAATCCAACACTACACACAAGTTCATTGACAAGTATGTTTTTAGGTTCTAATTTTTTCTACATTTTGAAGGTGTTGGCACCACTTCCCATTGGGTTTA  800
HAU|A01_116027001-116028131|+  TTCAATCCAACACCACACACAAGTTCATTGACAAGTATGTTTTTAGGGACTAATTTTTTCTACATCTTGAAGGTGTTGGCACCACTTCCCATTGGGTTTG  800
HAU|D01_61617884-61619012|+    TTCAATCCAACACCACACACAAGTTCATTGACAAGTATGTTTTTAGGGACTAATTTTTTCTACATTTTGAAGGTGTTGGCACCACTTCC-ATTGGGTTTG  799
HAU|D01_61609329-61610459|+    TTCAATCCAACACCACACACAAGTTCATTGACAAGTATGTTTTTAGGGACTAATTTTTTCTACATTTTGAAGGTGTTGGCACCACTTCCCATTGGGTTTG  800

NAU|A01_98366726-98367857|+    CTGTGTTTATGGTTCATTTGGCAACTATTCCGATCACCGGAACCGGAATCAACCCTGCTCGTAGTTTCGGAGCTGCTGTTATGTTCAACCAGGACAAGCC  900
NAU|A01_98361882-98363012|+    CTGTGTTTATGGTTCATTTGGCAACTATTCCGATCACCGGAACCGGAATCAACCCTGCTCGTAGTTTCGGAGCTGCTGTTATGTTCAACCAGGACAAGCC  900
NAU|D01_59898611-59899741|+    CTGTGTTTATGGTTCATTTGGCAACTATTCCGATCACCGGAACCGGAATCAACCCTGCTCGTAGTTTCGGAGCTGTTGTTATGTTCAACCAGGACAAGCC  900
NAU|D01_59890054-59891184|+    CTGTGTTTATGGTTCATTTGGCAACTATTCCGATCACCGGAACCGGAATCAACCCTGCTCGTAGTTTCGGAGCTGTTGTTATGTTCAACCAGGACAAGCC  900
ZJU|A01_116473274-116474405|+  CTGTGTTTATGGTTCATTTGGCAACTATTCCGATCACCGGAACCGGAATCAACCCTGCTCGTAGTTTCGGAGCTGCTGTTATGTTCAACCAGGACAAGCC  900
ZJU|A01_116468430-116469560|+  CTGTGTTTATGGTTCATTTGGCAACTATTCCGATCACCGGAACCGGAATCAACCCTGCTCGTAGTTTCGGAGCTGCTGTTATGTTCAACCAGGACAAGCC  900
ZJU|D01_63074368-63075498|+    CTGTGTTTATGGTTCATTTGGCAACTATTCCGATCACCGGAACCGGAATCAACCCTGCTCGTAGTTTCGGAGCTGTTGTTATGTTCAACCAGGACAAGCC  900
ZJU|scaffold_D01_84-385/46-787 CTGTGTTTATGGTTCATTTGGCAACTATTCCGATCACCGGAACCGGAATCAACCCTGCTCGTAGTTTCGGAGCTGTTGTTATGTTCAACCAGGACAAGCC  813
HAU|A01_116031840-116032971|+  CTGTGTTTATGGTTCATTTGGCAACTATTCCGATCACCGGAACCGGAATCAACCCTGCTCGTAGTTTCGGAGCTGCTGTTATGTTCAACCAGGACAAGCC  900
HAU|A01_116027001-116028131|+  CTGTGTTTATGGTTCATTTGGCAACTATTCCGATCACCGGAACCGGAATCAACCCTGCTCGTAGTTTCGGAGCTGCTGTTATGTTCAACCAGGACAAGCC  900
HAU|D01_61617884-61619012|+    CTGTGTT-ATGGTTCATTTGGCAACTATTCCGATCACCGGAACCGGAATCAACCCTGCTCGTAGTTTCGGAGCTGTTGTTATGTTCAACCAGGACAAGCC  898
HAU|D01_61609329-61610459|+    CTGTGTTTATGGTTCATTTGGCAACTATTCCGATCACCGGAACCGGAATCAACCCTGCTCGTAGTTTCGGAGCTGTTGTTATGTTCAACCAGGACAAGCC  900

NAU|A01_98366726-98367857|+    ATGGGATGACCATGTATGAACCTTCATTTATACAATTGATTTCTATTTTTTTTCTTTTGTTTTATACGGCTCCTATTAAACATATTTTTATATCTGTTT  1000
NAU|A01_98361882-98363012|+    ATGGGATGACCATGTATGAACCTTCATTTATACAATTGATTTCTATTTTTTTGCTTT-GTTTTATACGGCTCCTATTAAACATATTTTTATATCTGTTT  999
NAU|D01_59898611-59899741|+    ATGGGATGACCATGTATGAAACTTCATTTATACAATTGATTTCTATTGTTTTCCTTG-GTTTAATACGGCTCCTATTAAACATTTTTTTATATCTGTTT  999
NAU|D01_59890054-59891184|+    ATGGGATGATCATGTATGAACCATCATTTATACAATTGATTTCTATTGTTTTCCTTT-GTTTAATACGGCTCCTATTAAACATATTTTTATATCTGTTT  999
ZJU|A01_116473274-116474405|+  ATGGGATGACCATGTATGAACCTTCATTTATACAATTGATTTCTATTTTTTTTCTTTTGTTTTATACGGCTCCTATTAAACATATTTTTATATCTGTTT  1000
ZJU|A01_116468430-116469560|+  ATGGGATGACCATGTATGAACCTTCATTTATACAATTGATTTCTATTTTTTTGCTTT-GTTTTATACGGCTCCTATTAAACATATTTTTATATCTGTTT  999
ZJU|D01_63074368-63075498|+    ATGGGATGACCATGTATGAAACTTCATTTATACAATTGATTTCTATTGTTTTCCTTG-GTTTAATACGGCTCCTATTAAACATTTTTTTATATCTGTTT  999
ZJU|scaffold_D01_84-385/46-787 ATGGGATGATCATGTATGAACCATCATTTATACAATTGATTTCTATTGTTTTCCTTT-GTTTAATACGGCTCCTATTAAACATATTTTTATATCTGTTT  912
HAU|A01_116031840-116032971|+  ATGGGATGACCATGTATGAACCTTCATTTATACAATTGATTTCTATTTTTTTTCTTTTGTTTTATACGGCTCCTATTAAACATATTTTTATATCTGTTT  1000
HAU|A01_116027001-116028131|+  ATGGGATGACCATGTATGAACCTTCATTTATACAATTGATTTCTATTTTTTTGCTTT-GTTTTATACGGCTCCTATTAAACATATTTTTATATCTGTTT  999
HAU|D01_61617884-61619012|+    ATGGGATGACCATGTATGAAACTTCATTTATACAATTGATTTCTATTGTTTTCCTTG-GTTTAATACGGCTCCTATTAAACATTTTTTTATATCTGTTT  997
HAU|D01_61609329-61610459|+    ATGGGATGATCATGTATGAACCATCATTTATACAATTGATTTCTATTGTTTTCCTTT-GTTTAATACGGCTCCTATTAAACATATTTTTATATCTGTTT  999

NAU|A01_98366726-98367857|+    GGAATATTGCAGTGGATATTCTGGGTTGGACCATTCATTGGAGCTGCCATTGCTGCAACTTATCACCAGTATATCCTCAGAGCAGGAGCTGCTAAAGCTC  1100
NAU|A01_98361882-98363012|+    GGAATATTGCAGTGGATATTCTGGGTTGGACCATTCATTGGAGCTGCCATTGCTGCAATTTATCACCAGTATATCCTCAGAGCAGGAGCTGCTAAAGCTC  1099
NAU|D01_59898611-59899741|+    GGAATATTGCAGTGGATATTCTGGGTTGGACCATTCATTGGAGCTGCCATTGCTGCAATTTATCACCAGTATATCCTCAGAGCAGGAGCTGCTAAAGCTC  1099
NAU|D01_59890054-59891184|+    GGAGTATTGCAGTGGGTATTCTGGGTTGGACCATTCATTGGAGCTGCCATTGCTGCAATTTATCACCAGTATATCCTCAGAGCAGGAGCTGCTAAAGCTC  1099
ZJU|A01_116473274-116474405|+  GGAATATTGCAGTGGATATTCTGGGTTGGACCATTCATTGGAGCTGCCATTGCTGCAACTTATCACCAGTATATCCTCAGAGCAGGAGCTGCTAAAGCTC  1100
ZJU|A01_116468430-116469560|+  GGAATATTGCAGTGGATATTCTGGGTTGGACCATTCATTGGAGCTGCCATTGCTGCAATTTATCACCAGTATATCCTCAGAGCAGGAGCTGCTAAAGCTC  1099
ZJU|D01_63074368-63075498|+    GGAATATTGCAGTGGATATTCTGGGTTGGACCATTCATTGGAGCTGCCATTGCTGCAATTTATCACCAGTATATCCTCAGAGCAGGAGCTGCTAAAGCTC  1099
ZJU|scaffold_D01_84-385/46-787 GGAGTATTGCAGTGGGTATTCTGGGTTGGACCATTCATTGGAGCTGCCATTGCTGCAATTTATCACCAGTATATCCTCAGAGCAGGAGCTGCTAAAGCTC  1012
HAU|A01_116031840-116032971|+  GGAATATTGCAGTGGATATTCTGGGTTGGACCATTCATTGGAGCTGCCATTGCTGCAACTTATCACCAGTATATCCTCAGAGCAGGAGCTGCTAAAGCTC  1100
HAU|A01_116027001-116028131|+  GGAATATTGCAGTGGATATTCTGGGTTGGACCATTCATTGGAGCTGCCATTGCTGCAATTTATCACCAGTATATCCTCAGAGCAGGAGCTGCTAAAGCTC  1099
HAU|D01_61617884-61619012|+    GGAATATTGCAGTGGATATTCTGGGTTGGACCATTCATTGGAGCTGCCATTGCTGCAATTTATCACCAGTATATCCTCAGAGCAGGAGCTGCTAAAGCTC  1097
HAU|D01_61609329-61610459|+    GGAGTATTGCAGTGGGTATTCTGGGTTGGACCATTCATTGGAGCTGCCATTGCTGCAATTTATCACCAGTATATCCTCAGAGCAGGAGCTGCTAAAGCTC  1099

NAU|A01_98366726-98367857|+    TTGGATCCTTCAGAAGCAGCTCAGCCATGTAA  1132
NAU|A01_98361882-98363012|+    TTGGATCCTTCAGAAGCAGCTCAGCCATGTAA  1131
NAU|D01_59898611-59899741|+    TTGGATCCTTCAGAAGCAGCTCAGCCATGTAA  1131
NAU|D01_59890054-59891184|+    TTGGATCCTTCAGAAGCAGCTCAGCCATGTAA  1131
ZJU|A01_116473274-116474405|+  TTGGATCCTTCAGAAGCAGCTCAGCCATGTAA  1132
ZJU|A01_116468430-116469560|+  TTGGATCCTTCAGAAGCAGCTCAGCCATGTAA  1131
ZJU|D01_63074368-63075498|+    TTGGATCCTTCAGAAGCAGCTCAGCCATGTAA  1131
ZJU|scaffold_D01_84-385/46-787 TTGGATCCTTCAGAAGCAGCTCAGCCATGTAA  1044
HAU|A01_116031840-116032971|+  TTGGATCCTTCAGAAGCAGCTCAGCCATGTAA  1132
HAU|A01_116027001-116028131|+  TTGGATCCTTCAGAAGCAGCTCAGCCATGTAA  1131
HAU|D01_61617884-61619012|+    TTGGATCCTTCAGAAGCAGCTCAGCCATGTAA  1129
HAU|D01_61609329-61610459|+    TTGGATCCTTCAGAAGCAGCTCAGCCATGTAA  1131
```
