# Supplementary material for: Combining genome-wide and transcriptome-wide analyses reveal the evolutionary conservation and functional diversity of aquaporins in cotton
Source: BMC Genomics. 2019 Jul 1;20:538. doi: 10.1186/s12864-019-5928-2 (PMC6604486; doi:10.1186/s12864-019-5928-2)
Supplement: Supplementary file 13 — Table S7. Primers information for quantitative real-time PCR analysis. (DOCX 14 kb) [file 12864_2019_5928_MOESM13_ESM.docx]

| **Additional file 13: Table S7. Primers information for quantitative real-time PCR analysis.** | | |
| --- | --- | --- |
| Gene name* | Forward primer (5'-3') | Reverse primer (5'-3') |
| *GrPIP2;5a* | ATGGTTACAGCACAGGAA | TGGGAGTCTCTAGCCTTC |
| *GrTIP2;1* | GATCACTCGGGACCATTG | GATGAGACCAGCCAATCC |
| *GrTIP1;1c* | GATGGAGCCTCAATGAAC | TGAACTCGTAGATGAGACC |
| *GrSIP1;2* | CTTAAAGGTCCAAAGAGTGAAA | CCATGTGTTGTGCCAATT |
| *GrPIP2;4a* | ATTGGATTTGCCGTGTTC | TCTTAGGATGTATTGGTGGTAA |
| *GrPIP2;2c* | GTTCACTTAGCCACTATTCC | CGATGCCCTCAATATAAACTG |
| *GrPIP1;4c* | GTCCACTTGGCAACAATT | AGGTCCTAACCAGAATATCC |
| *GrNIP1;2c* | TATATCTGATGTCACCAACACT | TACGAGGACTCTTGAGGAA |
| *GhPIP2;5* | GGCTCGTAAGGTCTCATT | TGCTGTAACCATCTGCTA |
| *GhTIP2;1* | CAAGGAGTGGTGATGGAG | CAACAATGAAGCCGATGG |
| *GhTIP1;1c* | GTGTCTAACGCATTGGTT | GGTTCATTGAGGCTCCAT |
| *GhSIP1;2* | GGTGATGCCAGAACAGTA | CCAGACATAACTATTGCTACAG |
| *GhPIP2;4a* | GTTCTCAGCGAAGGACTA | CAACACCGTGACATACAAG |
| *GhPIP2;2c* | GTTCACTTAGCCACTATTCC | ATGCCCTCAATACAAACTG |
| *GhPIP1;4c* | CCAAGATACCAAGGACTACA | TAGCGACACCCATTACAG |
| *GhNIP1;2c* | GCGGTGGTAGTGAATGTA | CCAGAGATGTGACCTAAGG |
| *GhHis3* | CGGTGGTGTGAAGAAGCCTCAT | AATTTCACGAACAAGCCTCTGGAA |

****Gr and Gh indicated G. raimondii and G. hirsutum, respectively.***
